# Non-Cytotoxic Benzyl Triphenyl Phosphonium Bromide Is Bactericidal on MRSA and Fully Inhibits Biofilm Formation by MRSA and MRSE

**DOI:** 10.3390/ph19060829

**Published:** 2026-05-26

**Authors:** Silvana Alfei, Gabriella Piatti, Guendalina Zuccari, Caterina Reggio, Anna Maria Schito

**Affiliations:** 1 Department of Pharmacy (DIFAR), University of Genoa, Viale Cembrano, 4, 16148 Genoa, Italy; guendalina.zuccari@unige.it; 2 Department of Surgical Sciences and Integrated Diagnostics (DISC), University of Genoa, Viale Benedetto XV, 6, 16132 Genova, Italy; gabriella.piatti@unige.it; 3 Laboratory of Experimental Therapies in Oncology, IRCCS Istituto Giannina Gaslini, Via G. Gaslini 5, 16147 Genoa, Italy; caterinareggio@gaslini.org

**Keywords:** multidrug-resistant bacteria, triphenyl phosphonium (TPP) group, minimum inhibitory concentration (MIC), bacteriostatic and bactericidal effects, time-killing experiments, biofilm (BF) formation inhibiting properties

## Abstract

**Background**. Positively charged quaternary phosphonium salts (QPSs) represent new potent weapons to selectively counteract critical superbugs, regardless of their profile of resistance, acting as membrane disruptors. QPSs **1**, **3** and **4**, and not cationic phosphine **2**, recently synthesized, characterized and evaluated for anticancer and cytotoxic effects, were morphologically and microbiologically evaluated. **Methods**. DLS analysis, minimum inhibitory concentrations (MICs) measurements, time-kill experiments and tests to evaluate biofilm (BF) formation inhibition, on Gram-positive and Gram-negative clinical superbugs, were carried out. **Results and Discussion**. All compounds demonstrated positive ζ-p = +4.2–+38.1 mV, while **2**, **3** and **4** showed nanovesicles of 140, 157, and 605 nm, in water solution. Interesting microbiologic results were obtained for compound **1**. Despite not being active against Gram-negative MDR isolates, **1** displayed MICs = 16–32 µg/mL and 16–64 µg/mL against methicillin-susceptible (MSSA) *Staphylococcus aureus* ATCC 29213, methicillin-resistant *S. aureus* (MRSA) and *S. epidermidis* (MRSE) respectively, while MICs = 32–64 µg/mL were observed against teicoplanin- and vancomycin-resistant (VRE) *Enterococcus faecalis* and *E. faecium*, thus overturning MICs previously reported for **1**. Novel time-kill experiments established the bactericidal effects of **1** against MRSA within 11 h, without no regrowth in the subsequent 24 h. Further, **1** inhibits up to 100% BF formation by the strongest BF-producers, *S. epidermidis* and *S. aureus* isolates, of our collection. **Conclusions**. All these antibacterial properties and low cytotoxicity on both fibroblasts (3T3) and human keratinocytes (HaCaT) cells make **1** appear as a potential new weapon to treat infections no longer affordable with current antibiotics; it is also thinkable for future use in vivo experiments and clinical development.

## 1. Introduction

The overprescription and misuse of antibiotics in both humans and animals, their use without proper medical indication, poor drug quality, and patients’ failure to complete the full course of treatment strongly promote genetic mutations in microorganisms, leading to the development of resistance to multiple antibiotics [1,2,3,4,5,6,7]. In this context, multidrug-resistant (MDR) superbugs are pathogens that have developed resistance to multiple classes of antibiotics, sometimes to all available treatments. This newly acquired resistance to several antibiotics at once is leading to difficult-to-treat infections, which are associated with prolonged hospitalization, increased morbidity and mortality, and significant economic burden [8,9,10,11,12]. Poor hygiene and scarce sanitation in certain geographic areas can further worsen an already critical scenario [13].

MDR strains primarily emerge in hospital settings, where their infections often progress to severe clinical conditions requiring aggressive and prolonged therapeutic regimens [4]. Pneumonia, bloodstream infections, and urinary tract infections (UTI) can turn into otherwise treatable conditions and life-threatening diseases. They are particularly dangerous for vulnerable patients, including individuals with diabetes, severe burn injuries, organ transplants, and other compromised health conditions [5]. Great concern is associated with methicillin-resistant *Staphylococcus aureus* (MRSA), methicillin-resistant *Staphylococcus epidermidis* (MRSE) and vancomycin-resistant *Enterococcus faecium* and *E. faecalis* (VRE), all classified among high-priority pathogens and also endowed with strong biofilm (BF)-forming capability [5].

MRSA is one of the most clinically significant MDR pathogens worldwide. Initially restricted to healthcare environments (HA-MRSA), it has now become widely established within the community (CA-MRSA), where it causes a broad spectrum of diseases ranging from skin and soft tissue infections to severe invasive conditions such as bacteremia, osteoarticular infections, and endocarditis [14].

The pathogen’s ability to form BFs and to acquire additional resistance determinants further complicates treatment, limiting therapeutic options to agents such as vancomycin, linezolid, daptomycin, and newer anti-MRSA compounds [15,16].

*S. epidermidis* is the most prevalent coagulase-negative *Staphylococcus* species colonizing human skin and mucosal surfaces. In its natural ecological niche, it generally behaves as a harmless commensal microorganism. However, it can act as an opportunistic pathogen when introduced into normally sterile sites, particularly through medical devices such as intravascular catheters, prosthetic heart valves, cardiac devices, orthopedic implants, and cerebrospinal fluid shunts [17,18], thus causing both localized and systemic infections, often associated with significant and even lethal clinical complications, requiring high hospitalization times and costs, with negative economic impact. Owing to the increasing prevalence of MRSE strains, they are also capable of forming BF on abiotic surfaces, like MRSA [19,20], augmenting the virulence factors of this species. BF enhances bacterial persistence by protecting cells from host immune responses and antimicrobial therapy, thus limiting therapeutic options and complicating clinical management, thereby rendering infections particularly difficult to eradicate [21].

*E. faecalis* and *E. faecium* strains represent other major opportunistic pathogens in healthcare settings. Although they are part of the normal human gastrointestinal microbiota, they have emerged as leading causes of healthcare-associated infections, including UTI, bacteremia, endocarditis, intra-abdominal infections, and device-associated infections [22,23]. Their clinical importance stems not only from intrinsic resistance to several antibiotics, such as cephalosporins and aminoglycosides, but also from their remarkable capacity to acquire additional resistance determinants. Of particular concern is the global dissemination of vancomycin-resistant enterococci (VRE), which severely limits treatment options and is linked to increased morbidity, mortality, and healthcare burdens [24]. Furthermore, enterococci can form BFs on medical devices, contributing to chronic, difficult-to-treat infections [25]. Their adaptability and resistance profile make them a significant public health challenge.

The global spread of MRSA, MRSE, and VRE highlights their clinical relevance and underscores the urgent need for improved surveillance, infection control strategies, and the development of novel antimicrobial therapies.

The currently available therapeutic armamentarium against MRSA, MRSE, and VRE pathogens is nowadays limited. The reduced effectiveness of cornerstone agents, such as vancomycin, linezolid, and daptomycin, increasingly promotes persistent BF-associated and device-related infections, often necessitating combination therapies or the use of newer agents supported by variable clinical evidence. These challenges highlight the urgent need for optimized antimicrobial stewardship and the development of innovative therapeutic strategies targeting both resistance mechanisms and BF formation.

**Scheme 1:**
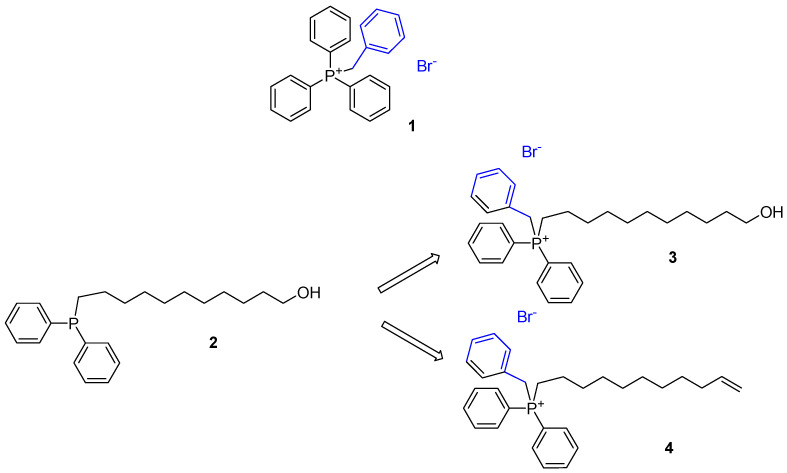
Chemical structures of compounds **1**–**4**. Not quaternized compound **2** was prepared since a precursor was needed for achieving compounds **3** and **4**. Arrows indicate the compounds which were synthesized using **2**. In blue colors are indicated the benzyl groups which were used to quaternize phosphine precursors to transform them in bromide salts.

To meet these needs, we considered positively charged QPSs **1**, **3** and **4**, already found selectively active against cancer cells, and therefore promising as antibacterial agents [26] (Scheme 1).

Not cationic phosphine compound **2**, but anyway positively charged (ζp = +4.19 mV), available since synthesized as precursor for achieving compounds **3** and **4**, was also investigated (Scheme 1). Despite incorrect results, here verified and confuted, the antibacterial effects of compound **1** were already reported [27], while compound **2** was never biologically tested and compounds **3** and **4** were never microbiologically evaluated.

### 1.1. Roadmap Followed to Assess the Antibacterial Effects of Compounds ***1**–**4***

**Figure 1:**
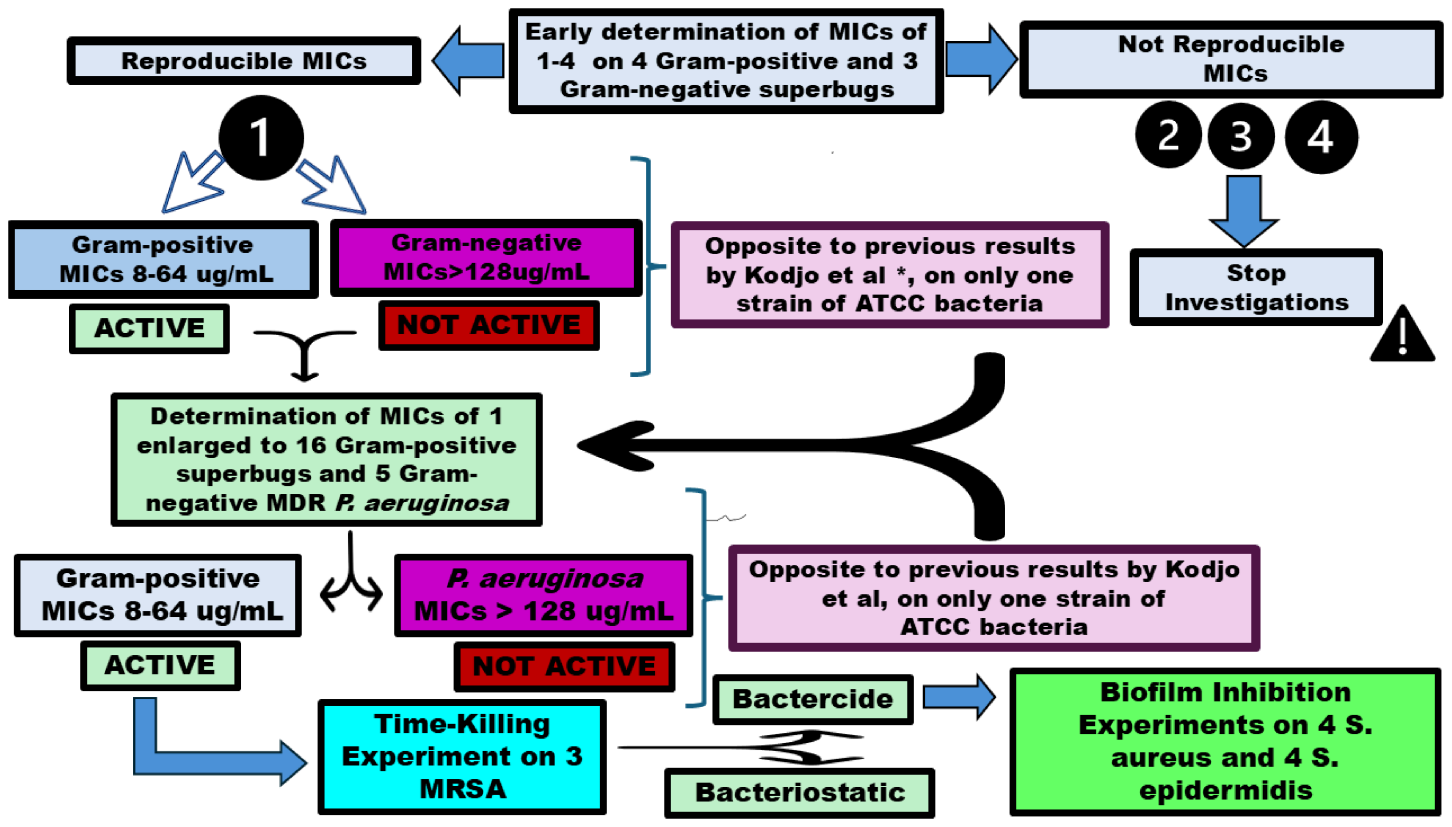
Roadmap followed to microbiologically evaluate QPSs **1**–**4**, recently synthesized [26]. * Kodjo et al. [27].

Experiments were conducted to evaluate the antibacterial behavior of the synthesized compounds **1**–**4** (Scheme 1), starting from determining their minimum inhibitory concentrations (MICs) on a narrow selection of multidrug-resistant (MDR) Gram-positive and Gram-negative clinical isolates and following the roadmap schematized in Figure 1.

Compounds **1**, **2**, **3** and **4** were assayed against selections of Gram-positive and Gram-negative clinical superbugs, having a very complex profile of cross-resistance and one methicillin-susceptible *S. aureus* ATCC 29213 (MSSA), for comparisons. All bacteria used in this study, except for the ATCC strain, were clinical isolates that had developed resistance to at least one or two antibiotics, including ESKAPE species.

Despite several repeated determinations, no modal reliable MIC values (MICs) were observed for **2**, **3** and **4**, due to their poor solubility in the medium. This issue forced us to not further consider them in this study and postpone further attempts after proper chemical modifications to enhance their solubility, which is not in the scope of the present study. Conversely, promising MICs for **1** were observed on MDR Gram-positive clinical isolates. In fact, despite MICs > 128 µg/mL being observed against all Gram-negative MDR isolates tested in this study (7 strains), including MDR *E. coli*, *K. pneumoniae* and *P. aeruginosa*, thus establishing inactivity, **1** demonstrated moderate to good activity against all Gram-positive *Staphylococci* and *Enterococci*, regardless of the hard resistance profile of 15 out of 16 strains. Compound **1** was therefore retained as worthy of immediate deeper investigations, on which our attention and this study were focused. Our choice of MIC > 128 µg/mL as the cut-off limit to define new compounds as active depended on the practical MIC threshold range (<2 up to 64 µg/mL), under which new antibacterial compounds can be considered potent to weak antibacterial agents against MDR clinical bacteria (ESKAPE, *E. coli* ESBL, *K. pneumoniae KPC*, *A. baumannii, P. aeruginosa*, MRSA, MRSE and VRE), according to thresholds commonly used in drug discovery and medicinal chemistry [28,29]. Since compounds with MIC over 64 µg/mL are considered not competitive, rationally, those displaying MICs > 128 µg/mL should be considered inactive. Collectively, findings on compound **1** were opposite to previous reports by Kodjo et al. [27]. Investigations were extended to more strains of the same Gram-positive species tested in early experiments. Additionally, five MDR *P. aeruginosa* clinical isolates were tested to confirm or confute the negative results obtained by us in the early screening, thus definitely establishing the actual antibacterial effects of **1**. Upon confirmation of early results, as relevant novelty regarding **1**, time-kill curves were determined at 24 h from inoculum using different MRSA isolates to establish its bacteriostatic or bactericidal behavior. Additionally, experiments assessing its capacity to inhibit BF formation were carried out using four *plus* four clinical MRSA and MRSE isolates among the strongest BF-producers of our collection (OD_570_ up to >>> 3).

Structural considerations and the possibility of π-resonance and π-acceptor effects, leading to the stabilization by delocalization on *ortho*, *para* and *ortho* positions of cationic charges with large ionic radii, also on phenyl rings of **2**, as reported for QPSs [30], justified the positive ζp (mV) measured for **2**, although originally not cationic.

### 1.2. Why Just Phosphonium Salts ***1***, ***3*** and ***4***, as Well as Phosphine ***2***?

Cationic compounds frequently display both antibacterial and anticancer activities, due to their ability to selectively interact with negatively charged cell membranes. Multiple studies have demonstrated that the positive charge promotes electrostatic attraction toward the negatively charged internal membrane of bacteria and surface of tumor cells, which are enriched in anionic groups, more than the membrane of eukaryotic normal human cells, thus allowing strong selective adhesion. These events induce membrane potential and lipid bilayer destabilization, irreversible membrane damage, pore formation, progressive permeabilization, and, ultimately, cell death. Recent reviews highlight that many cationic antimicrobial peptides, originally developed for antibacterial purposes, can be repurposed as anticancer agents (and vice versa), because they share the same membrane-disruptive mechanism of action [31,32,33]. It has also been reported that surface charge neutralization and pore formation driven by cationic peptides or nanoparticles (NPs) represent a common mechanism explaining their efficacy against drug-resistant bacteria and highly aggressive tumor cells [33,34,35]. This mechanistic convergence makes cationic compounds particularly attractive as dual-action therapeutic platforms.

In this context, QPSs **1**, **3**, and **4** were recently shown to possess significant anticancer activity [26], suggesting that they could also exert antibacterial effects. Their selection is consistent with a rational, literature-supported approach. Numerous polymeric and non-polymeric quaternized phosphonium salts (PQPSs and QPSs) have been reported to display narrow- or broad-spectrum antibacterial and/or bactericidal activities, also against MDR phenotypes. These effects are generally mainly attributed to nonspecific membrane-disruptive mechanisms, like those mentioned above, in the case of phosphonium compounds, due to the lipophilic phosphorus cations and structural-dependent capability of these cations to enter bacterial cells, enabling the circumvention of classical bacterial resistance pathways [36,37,38,39,40,41,42,43,44]. Both poorly packed and sterically hindered tetra alkyl phosphonium salts (TAPILs), containing [PR_4_]^+^ (R = alkyl chains) cations, as well as triphenyl phosphonium salts containing [PPh_3_R]^+^ (R = alkyl chain) cationic heads, have shown antibacterial activity dependent on the anion identity (bromide, chloride, etc.), alkyl chain length, and steric hindrance. Depending on their structural features, such compounds display moderate to strong antibacterial activity against *S. aureus*, *E. coli*, *S. epidermidis*, *E. faecium*, MRSA, and *Acinetobacter baumannii*, in some cases comparable to standard commercial biocides such as benzalkonium chloride or the surfactant Triton-X-100 [42,44,45,46,47,48]. Notably, *bis*-phosphonium ionic liquids have demonstrated antimicrobial activity against clinically important ocular pathogens, including MDR *P. aeruginosa* and fungal isolates, although their high corneal cytotoxicity remains a limiting factor [49]. Furthermore, C3–C7 trialkyl phosphonium derivatives, carrying complex fourth substituents on the phosphorus atom, have exhibited significant antibacterial activity against *Bacillus subtilis*, alongside marked uncoupling activity in isolated rat liver mitochondria [50]. Reviews on the antimicrobial properties of other ionic liquid classes, including those active against BF formation, have also provided valuable insights [51,52]. Ermolaev et al. favored tri-tert-butyl substitution over the triphenyl phosphonium (TPP) group [42], but several TPP-, benzyl-, and alkyl benzyl-diphenyl phosphonium (DPP) derivatives have been shown to possess antibacterial activity, particularly against Gram-positive strains [27,53,54]. The strong antibacterial activity of an 11-hydroxyalkyl mono triphenyl phosphonium salt (TPPOH) against MDR Gram-positive species, and the even more potent bacteriostatic and anticancer properties of a bola-amphiphilic 1,12-*bis*-triphenylphosphonium dodecane bromide (BPPB) against both MDR Gram-positive and Gram-negative bacteria [37,38], combined with mild cytotoxicity toward eukaryotic cells and RBCs, have been recently reported [38,55,56,57,58]. Moreover, the strategic incorporation of a TPP-containing hexanoate moiety to chemically modify previously inactive triterpenoids has been shown to confer potent antibacterial activity against MRSA, MRSE, and vancomycin-resistant enterococci (VRE), including strains also resistant to teicoplanin [39]. All this literature data evidence that the spectrum of action of QPSs, and more specifically of TPP^+^- or DP^+^-containing QPSs, could strongly depend on their structure. Studies underscore that mono cationic phosphorous structures having alkyl-TPP^+^, alkyl-DP^+^-benzyl or TPP^+^-benzyl architectures, despite displaying even potent antibacterial/bactericidal effect, are active preferably against Gram-positive species. Conversely, for a broader activity against Gram-negative superbugs, the presence of more than one cationic head, preferably structured as alkyl-TPP^+^ groups, not containing benzyl moieties and having a balanced hindering on phosphorous cation, is required to allow easy penetration of the outer membrane to reach the cytoplasmic one. In this regard and according to our experience with QPSs, the structures of **1**–**4** forecast inactivity against Gram-negative species. However, the increasing emergence of MDR and biofilm (BF)-producing Gram-positive phenotypes, among simpler and more vulnerable wild bacteria, is selecting very strong strains, difficult-to-treat strains, such as Gram-negative ones, invalidating the effectiveness of most available antibiotics, making the need for new options to treat their lethal infections urgent.

Compound **1** is usually used in organic synthesis as a phase transfer catalyst, a Wittig reagent, and a Michael acceptor, but it is also applied in the production of pharmaceuticals, agrochemicals, and polymers, and has also been evaluated as a possible antibacterial [27]. Compounds **3** and **4**, never tested as possible antibacterials, despite not possessing the TPP group, shared anticancer properties and alkyl chains encompassing both ten carbon atoms, thus respecting the length usually reported to confer antibacterial activity to QPSs. Additionally, phenyl benzyl structures could confer them a correct lipophilicity and enlarged cationic radii [30], promoting detrimental interactions with bacteria surfaces. Collectively, **1**–**4** were different from already proposed compounds, since none of them contained the TPP^+^-group and the alkyl chain simultaneously, as seen in the most active and sometimes broader-spectrum compounds previously reported [37,38,44]. Moreover, they did not contain the hindering well-functioning tetra alkyl groups reported by Ermolaev et al. [42]. This strategy would have revealed if the simultaneous presence of the TPP^+^ group and of the alkyl chain, present in **3** and **4** but not in **1**, which conversely contained TPP^+^ but not the chain, is strictly necessary for having consistent antibacterial effects or if TPP alone could be sufficient. Compound **2**, which was primarily synthesized because of the need to achieve **3** and **4**, despite not being quaternized and not cationic, was also tested, since it demonstrated being nanosized and positively charged (ζ-p = +4.2 mV), and possibly capable of interacting with bacteria, thus impairing bacterial membrane functions and causing pathogen inhibition/death.

## 2. Results

### 2.1. Synthesis of Compounds ***1**–**4***

Chemical structures of phosphonium bromides **1**, **3** and **4**, as well as that of phosphine **2**, synthesized since a precursor was needed to achieve **3** and **4**, are shown in Scheme 1.

Quaternized phosphonium salts **1**, **3**, **4** and tertiary phosphine derivative **2** were synthesized according to Scheme S1A–C (Section S1, Supplementary Materials), performing procedures recently described [26].

As observable in Scheme 1, compounds **3** and **4** were different because **3** encompasses a hydroxyl on carbon atom C11, while **4** contains a C10–C11 double bond. The synthesis and full characterization of **1**–**4** have been described in our recent work [26]. Chemometric-assisted ATR-FTIR, ^1^H, ^13^C, ^31^P NMR and UV-Vis spectroscopies were used to confirm their structure and evaluate their level of purity [26]. The interpretation of NMR spectra and peak assignation was carried out with the help of the literature data [59] and of a ChemNMR H-1 Estimation tool associated with ChemDraw Ultra 7.0 software [26]. Copies of NMR spectra are available in Section S2 of the Supplementary Materials file associated with this study (Figures S1–S16), while those of ATR-FTIR ones are consultable in Section S3 (Figures S17–S20). Conversely, Figure S21 in Section S4 shows the copy of UV-Vis spectra of **1**–**4**.

### 2.2. Morphology of Compounds ***1**–**4*** in Methanol and Water by Optical Microscopy

Optical microscopy analyses were used to investigate the morphology of **1**–**4** in water suspension [26]. Optical images are available in Section S5 (Supplementary Materials) as Figure S22A–H.

### 2.3. Dynamic Light Scattering (DLS) Analyses of Compounds ***1**–**4***

Previous morphological experiments by optical microscopy in water dispersion evidenced the tendency of **1** to form microcrystalline aggregates and that of compounds **2**, **3** and **4** to form micro-vesicles (Section S5, Figure S22A–H, Supplementary Materials) [26]. Here, DLS analysis was carried out to precisely determine the hydrodynamic size (diameter) (Z_AVE_, nm) and polydispersity index (PDI) of vesicles of **2**, **3** and **4**, and to determine the Z-potential (ζ-p, mV) of all samples (**1**–**4**) to assess their surface charge. Particularly, ζ-p is connected to the stability of samples in solutions/dispersions [60,61] and to their possible capability to interact with the negatively charged surface of bacteria and their consequent antibacterial activity by membrane disruption [35,62]. Generally, higher values of ±ζ-p forecast higher stability [61], while higher values of +ζ-p correspond to higher possibility of interaction with bacterial membrane, of impairing it and of antibacterial activity [63]. Three measurements, made of ten runs (called records in DLS images) for each one, were carried out, and results of single or two records acquired by number (%) were reported in Figure S23A,C,E in Section S6 (Supplementary Materials). Specifically, reported Z_AVE_ (nm) in Table 1 is the mean ± S.D. of the records acquired at the highest kcps value. Similarly, PDI related to reported record was provided (Table 1). Additionally, zeta-potential (ζ-p) measurements for all samples (**1**–**4**) were carried out on their water dispersions to determine their surface charge. Provided ζ-p values (Table 1) were obtained by three measurements, made of 12 runs for each one, and were reported as mean ζ-p ± S.D. Three representative runs out of 12 were reported for their ζ-p (mV) distribution in Figure S23B,D,E, for compounds **2**–**4** and Figure S24, for compound **1**.

### 2.4. Antibacterial Properties of QPSs ***1**–**4***

#### 2.4.1. MICs Determinations

The results from the early screening experiments have been summarized in Table S1, available in Section S7 of Supplementary Materials.

As anticipated, **2**, **3** and **4** provided not legible MICs, due to their poor solubility, and were considered not suitable for further investigations, at least in this study, if not properly modified. For the moment, the actual activity of **2**, **3** and **4** remains a mystery.

Conversely, compound **1**, despite not being active against Gram-negative species, including *E. coli*, * K. pneumoniae* and *P. aeruginosa* (MIC > 128 µg/mL), was active against *S. aureus* ATCC 29213, MRSA and VRE *E. faecalis* (MICs = 16–32 and 64 µg/mL). Additionally, compound **1** was active against MRSE and VRE *E. faecium* (MICs = 16 and 64 µg/mL).

MICs using compound **1** were measured against an enlarged population of Gram-positive clinical isolates and additional Gram-negative *P. aeruginosa* superbugs.

Specifically, compound **1** was tested against Gram-positive isolates of *Staphylococcus* and *Enterococcus* species for a total of four different MDR clinical isolates for each species (16 strains), and against other MDR *P. aeruginosa* strains for a total of five clinical isolates (Table 2).

Results in Table 2, including reference antibiotics, confirmed the early finding in Table S1.

#### 2.4.2. Time-Kill Curves

**Figure 2:**
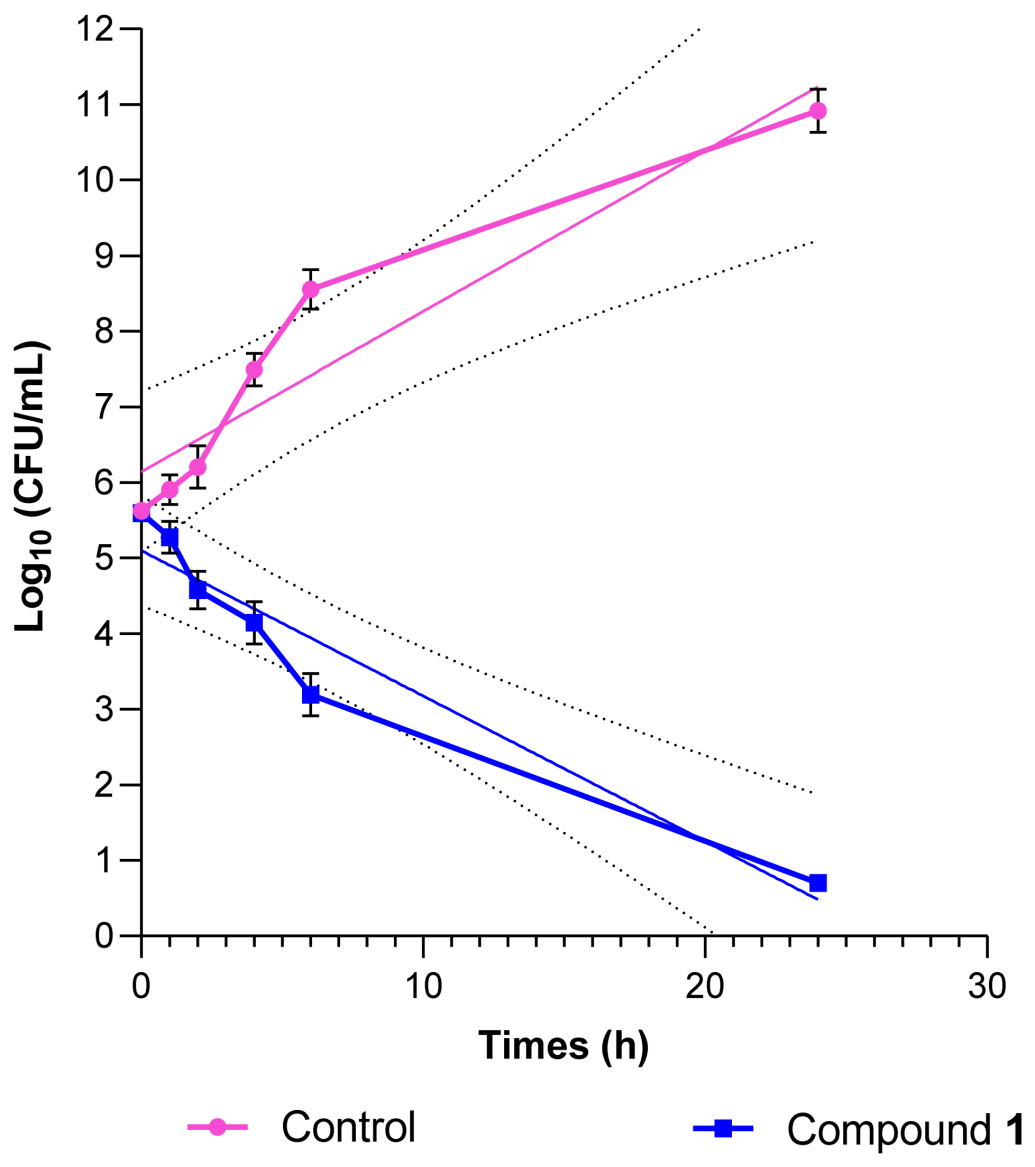
Time-kill curves obtained for control (pink line with round indicators) and for *S. aureus* 18 (blue line with square indicators) under treatment with **1** at concentrations 4 × MIC. Thinner lines in same colors are the associated linear tendency lines. Equation of thinner blue line was used to calculate the time at which the >3 log_10_ reduction in initial inoculum was reached. Punctuated curves indicate the confidence intervals (95%) around the tendency lines.

Here, compound **1** was used for the first time in time-kill experiments, at 4 × MICs for three selected strains (*S. aureus* ATCC 29213, MRSA 18 and MRSA 189). As depicted in Figure 2, reporting the most representative curve obtained for MRSA strain 18, **1** demonstrated bactericidal effects, as a decrease of >3 log_10_ occurred, within 11 h from inoculum (equivalent to 99.9% killing of the inoculum), while no regrowth was observed up to 24 h, when a 5 log_10_ reduction was reached.

Similar trends were observed for MSSA ATCC 29213 (sensitive to oxacillin) and MRSA 189, despite their different pattern of resistance and concentrations (MRSA 189). Anyway, to better evaluate the killing performance of **1**, a kinetic study was carried out, as detailed in the following section, to compare the rate of **1** killing with that of bacterial growth.

##### Kinetic Studies

To obtain valuable information on the pathways and mechanisms of bacterial growth in the control and bacterial death under treatment with **1** over time, we carried out a kinetic study, fitting the time-kill curves in Figure 2 with two of the mainly used mathematical models previously reported [64,65,66]. Specifically, kinetic models of pseudo-first order (PFO) [Equation (1)] and pseudo-second order (PSO) [Equation (2)] were studied.

(1) $$\ln\left(GKe-GKt\right)=lnGKe-k1\times t$$

(2) $$\frac{t}{GKt}=\frac{1}{k2\times GKe^{2}}+\frac{1}{GKe}t$$

where *GKe* (Log_10_ CFU/mL) and *GKt* (Log_10_ CFU/mL) are the bacteria colonies at the end and at time *t* respectively; *k*1 (*k_PFO_*) is the growth/killing kinetic constant of the PFO kinetic model (1/min), while *k*2 (*k_PSO_*) is that of the PSO kinetic model. Values of ln (*GKe* − *GKt*) and *t*/*GKt* were plotted vs. times. Dispersion graphs were obtained by using GraphPad software 8.0.1, which also provided their linear regression lines using the Ordinary Least Squares (OLS) method, together with their equations and related R^2^ values (Figure S25, Section S9, Supplementary Materials). The coefficients of determination (R^2^) of all the equations of the linear regressions obtained have been reported in Table S2 (Section S9, Supplementary Materials). They were used as the parameters for determining the kinetic models that best fit the data of time-kill (Figure 2). The results in Table S2 showed that in both cases (control and **1**), the highest R^2^ value was obtained for the PSO model. Consequently, the kinetic behavior of regrowth in the control and of MRSA killing by **1** followed the PSO kinetic model. The values of *GKe* and *K_PSO_* were computed using the values of the slopes and intercepts of the equations in Figure S25 and included in Table S3 (Section S9, Supplementary Materials).

The values of *K_PSO_* established that killing of bacteria under treatment with **1** occurred much more rapidly (by 7.2-times) than their regrowth in the control. Additionally, the values of *Ge* and *Ke* perfectly agreed with experimental ones, as the minimal differences of 0.43 (3.8%) and 0.0322 (4.6%) confirmed.

#### 2.4.3. Capacity of **1** to Inhibit BF Formation

##### BF Formation by the Strongest *Staphylococci* BF Producers of Our Collection Was Inhibited by **1**

**Figure 3:**
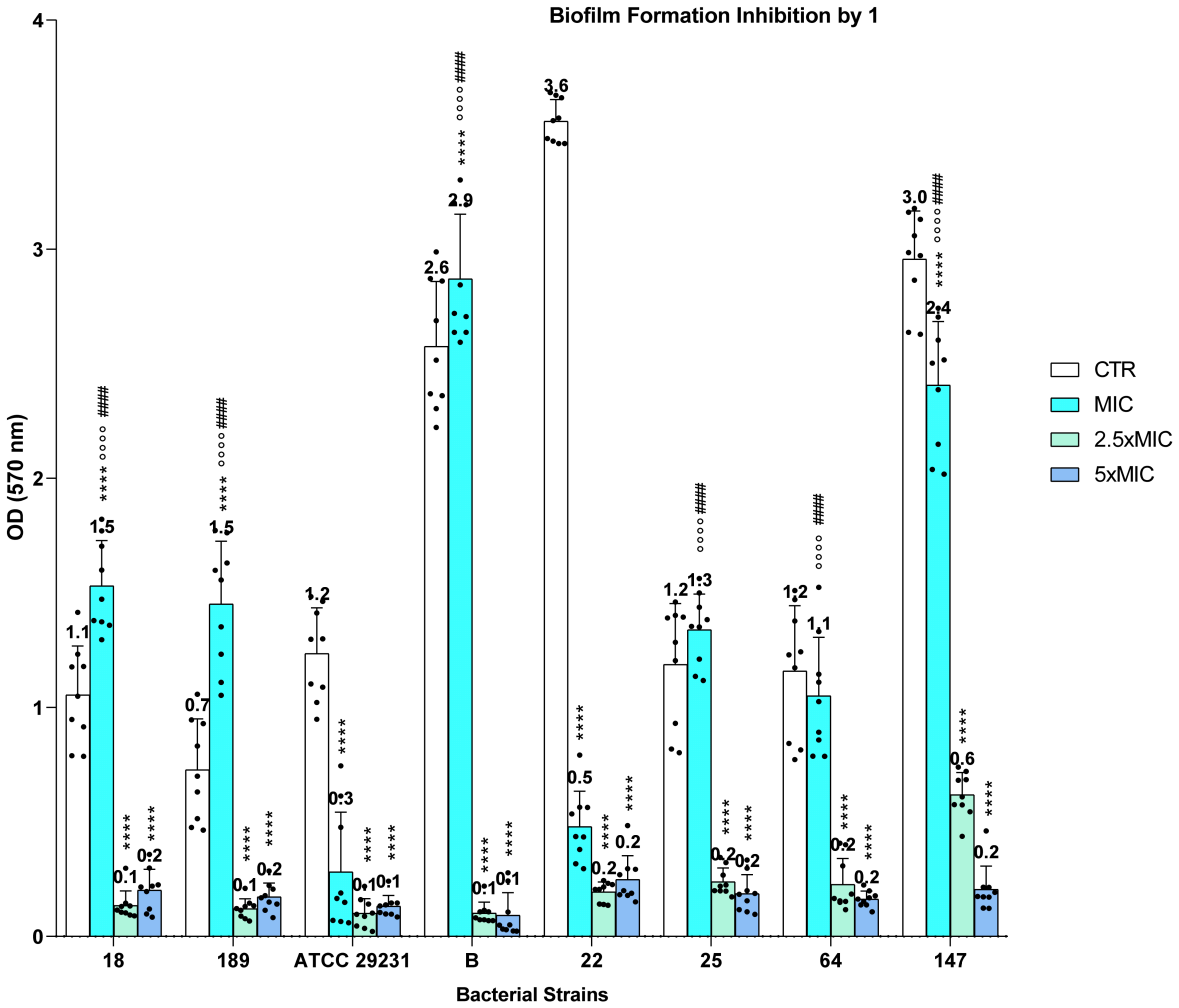
Inhibition of BF formation (OD 570 nm, OD_570_) by four *S. aureus* (18, 189, ATCC 29213 and B) and four *S. epidermidis* (22, 25, 64 and 147) species induced by compound **1**, administered to bacteria at MIC (sky-blue bars), 2.5 × MIC (light-blue bars) and 5 × MIC (blue bars). Statistical significance was obtained using GraphPad PRISM software 8.0.1 by the analysis of variance (Two-way ANOVA) corrected for multiple comparisons using statistical Tukey hypothesis testing. The statistical difference was reported for each isolate and for each concentration against control (CTR, white bars) using * symbol. Specifically, adjusted *p* values for multiplicity comparisons were reported for each comparison. Specifically, *p* > 0.05 no symbols; *p* < 0.0001 ****. Analogously, significant difference in MIC versus 2.5 and 5 × MIC was indicated using ° and # symbols, while no statistical difference was found in 2.5 × MIC versus 5 × MIC. Specifically, *p* > 0.05 no symbols; *p* < 0.0001 °°°° and *p* < 0.0001 ####. Numbers above bars at bottom indicate OD_570_ values.

**Figure 4:**
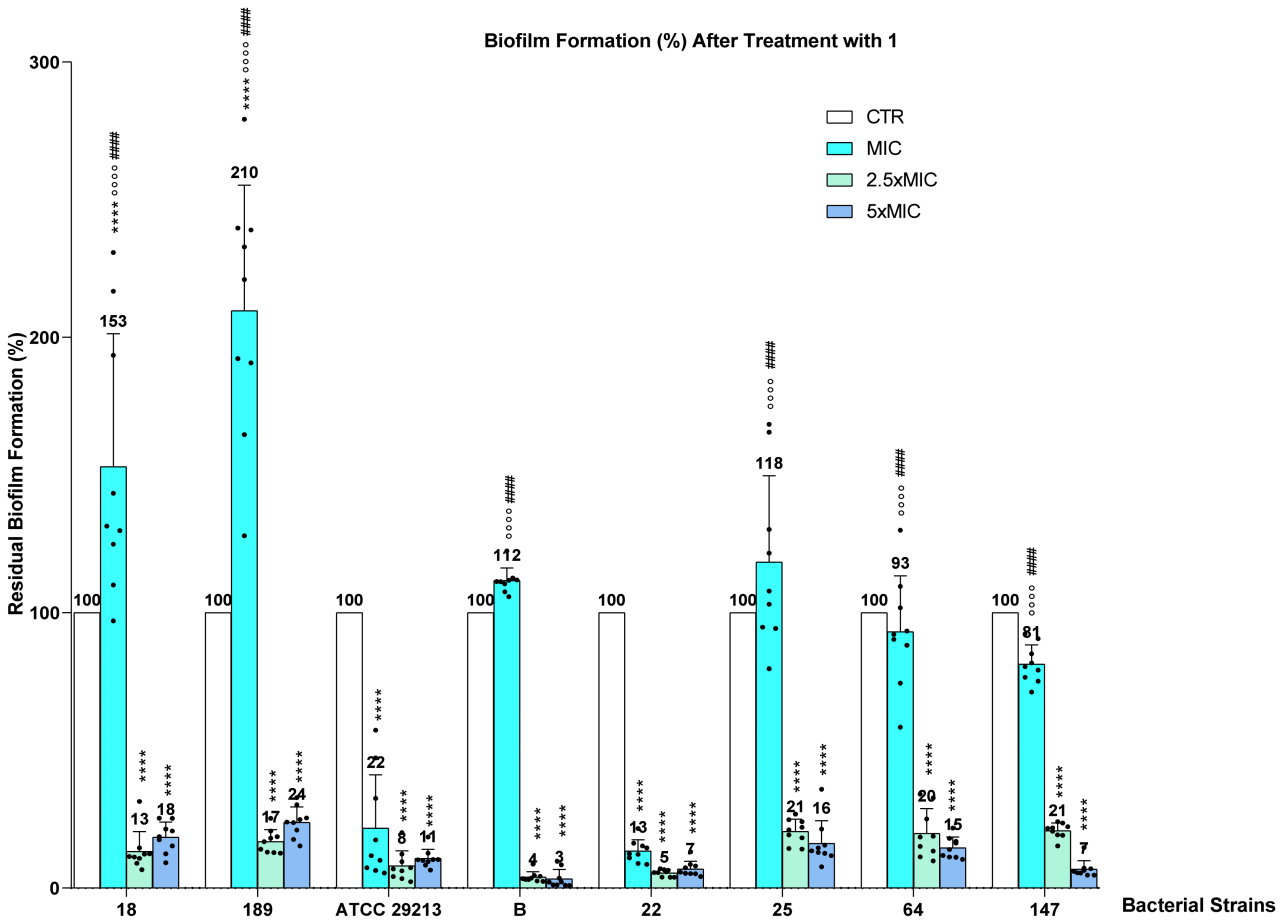
Residual BF formation (%) by *S. aureus* (18, 189, ATCC 29213 and B) and by *S. epidermidis* (22, 25, 64 and 147) species after treatments with compound **1** at MIC (sky-blue bars), 2.5 × MIC (light-blue bars) and 5 × MIC (blue bars). Statistical significance was obtained using GraphPad PRISM software 8.0.1 by the analysis of variance (Two-way ANOVA) corrected for multiple comparisons using statistical Tukey hypothesis testing. The statistical difference was reported for each isolate and for each concentration against control fixed to 100% (CTR, white bars) using * symbol. Adjusted *p* values for multiplicity comparisons were reported for each comparison. Specifically, *p* > 0.05 no symbols; *p* < 0.0001 ****. Analogously, significant difference in MIC versus 2.5 and 5 × MIC was indicated using ° and # symbols, while no statistical difference was found in 2.5 × MIC versus 5 × MIC. Specifically, *p* > 0.05 no symbols; *p* < 0.0001 °°°° and *p* < 0.0001 ####. Numbers above bars at bottom indicate OD_570_ values.

**Figure 5:**
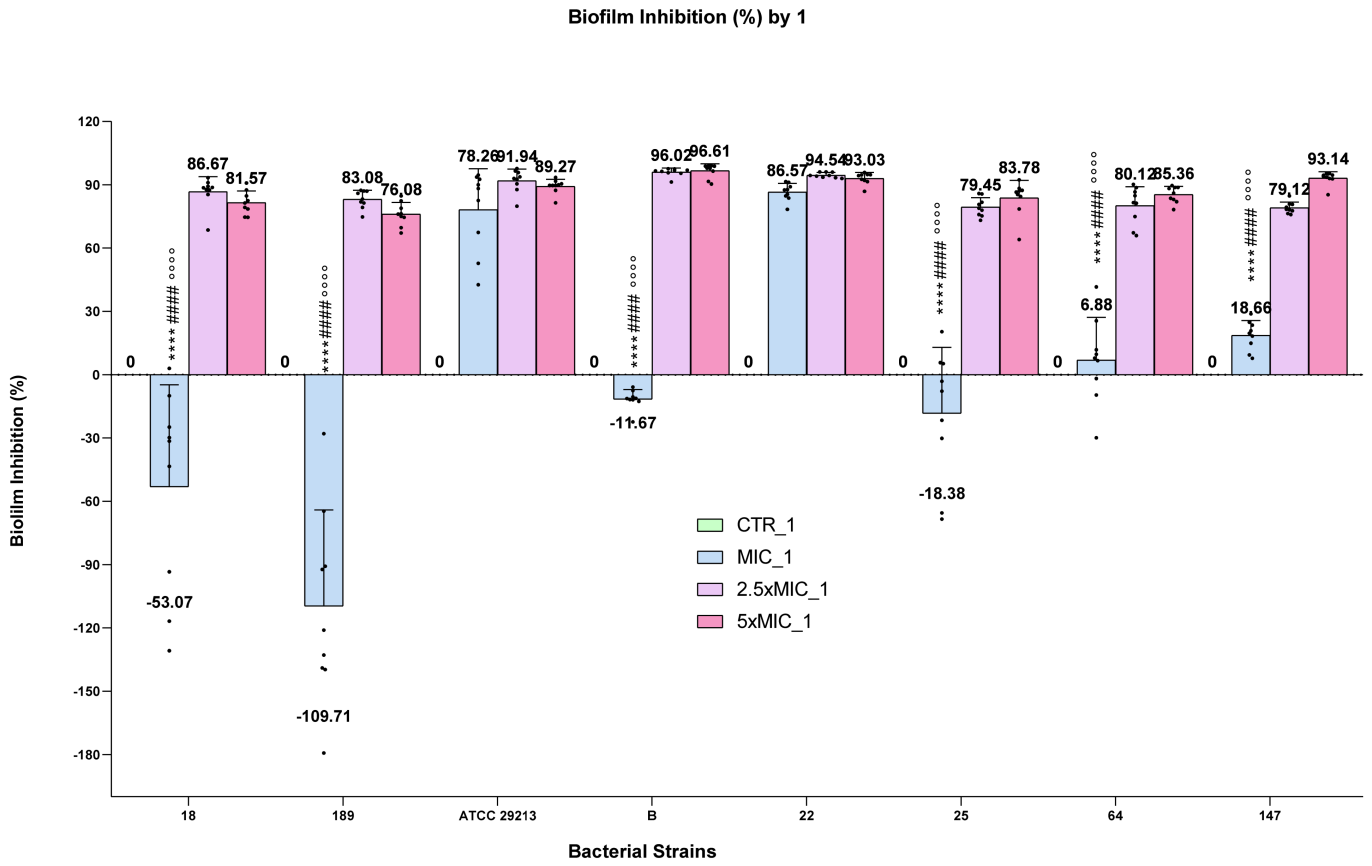
BF inhibition percentage (%) of *S. aureus* (18, 189, ATCC 29213 and B) and *S. epidermidis* (22, 25, 64 and 147) species after no treatment (0.1%) and after treatment with compound **1**, administered to bacteria at MIC (light blue bars), 2.5 × MIC (violet bars) and 5 × MIC (pink bars). Statistical significance was obtained using GraphPad PRISM software 8.0.1 by the analysis of variance (Two-ways ANOVA), corrected for multiple comparisons using statistical Tukey hypothesis testing. The statistical difference was reported for each isolate and for each concentration tested against control (CTR, green bars) using * symbol. Adjusted *p* values for multiplicity comparisons were reported for each comparison. Specifically, *p* > 0.05 no symbols; *p* < 0.0001 ****. Analogously, significant difference in MIC versus 2.5 and 5 × MIC was indicated using ° and # symbols, while no statistical difference was found in 2.5 × MIC versus 5 × MIC. Specifically, *p* > 0.05 no symbols; *p* < 0.0001 °°°° and *p* < 0.0001 ####. Numbers above bars at bottom indicate OD_570_ values.

To develop innovative ABF strategies specifically targeting the early adhesion and maturation processes of *S. aureus* and *S. epidermidis*, compound **1** was herein tested to assess its possible capacity to inhibit BF formation by eight strong BF-producers *Staphylococci*, at MIC, 2.5 × MIC and 5 × MIC (µg/mL). Vancomycin, to which all selected clinical isolates were sensitive, was used as the reference antibiotic under the same conditions of **1**. The following Figure 3, Figure 4 and Figure 5 show the BF formation inhibition (OD_570_), the residual BF (%) and the BF inhibition (%) observed when three MRSA, one MSSA ATCC 29213 strain (sensitive to oxacillin) and four MRSE BF producers were not treated, or treated for 24 h with **1** at MIC, 2.5 × MIC and 5 × MIC. Conversely, Figures S26 and S27 in Section S10 (Supplementary Materials) show the results obtained with vancomycin and **1** in single graphs, where the residual BF (%) and the BF inhibition (%) by same strains are shown.

### 2.5. Cytotoxicity of Compound ***1***

Cytotoxic effects of **1** were previously investigated against eukaryotic cells, such as HaCaT and 3T3 cells, for 24, 48 and 72 h, as reported in our recent article [26]. Figure S28 (Section S11, Supplementary Materials) shows the results of cytotoxicity experiments carried out on HaCaT (A) and those obtained on 3T3 cells (B), where the original concentrations of **1** (1–100 µM) were expressed as µg/mL.

Table S6 (Section S11, Supplementary Materials) reports the IC_50_ values of **1** when tested on HaCaT and 3T3 cells in 24 h treatments.

#### Selectivity of **1** for Bacteria

Selectivity of **1** for bacteria in relation to its cytotoxicity against HaCaT and 3T3 cells was assessed by calculating its selectivity index values (SIs). Generally, the SIs against bacteria cells (BAs) in relation to eukaryotic cells (ECs) is calculated using Equation (3), where BCs mean bacterial cells while ECs eukaryotic cells.

SI = MICs_(BCs)_/IC_50(ECs)_ (3)

**Figure 6:**
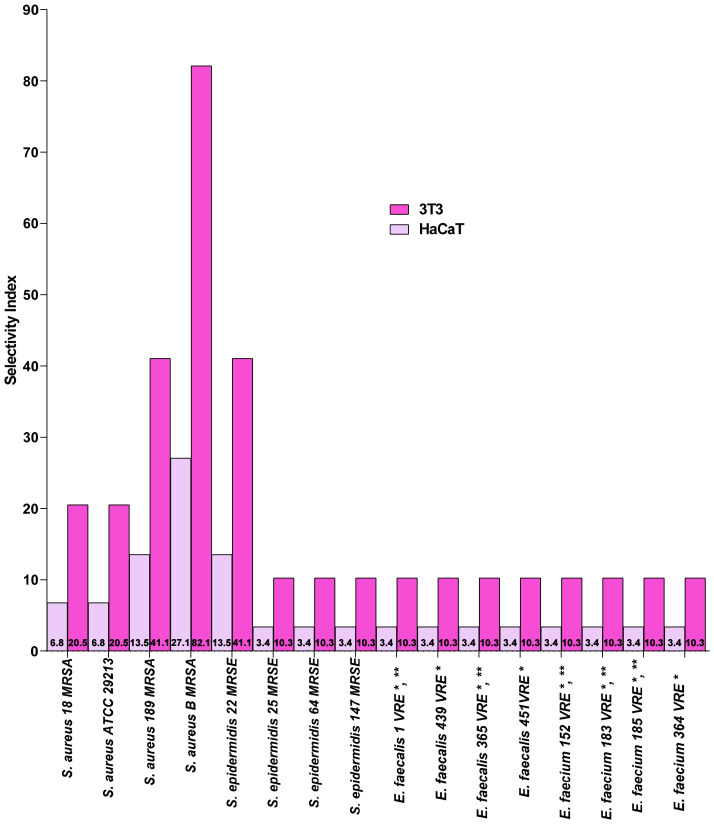
Selectivity index values (SIs) of **1** in relation to its cytotoxicity in HaCaT (purple bars) and 3T3 cells (light violet bars). VRE = vancomycin-resistant enterococci; MRSA = methicillin-resistant *S. aureus*; MRSE = methicillin-resistant *S. epidermidis*; * denotes resistance also to teicoplanin; ** denotes resistance also to linezolid. ATCC 29213 was a standard MSSA (sensitive to oxacillin). Numbers within bar at bottom indicate SIs specific for each bacterial isolate of this study.

The IC_50_ values reported in Table S6 were used to calculate the SIs of **1** against all Gram-positive clinical isolates used in this study according to Equation (3). Results have been reported in Table S7 (Section S12, Supplementary Materials), while Figure 6 reports selectivity of **1** for all strains in relation to its toxicity versus HaCaT and 3T3 cells.

## 3. Discussion

### 3.1. DLS Analyses Results

Size-related images evidenced the presence of more than one-dimensional family for all samples **2**–**4**. Two-dimensional families were observed for samples **2** and **3**, where the less representative (<1% by number) was made of micrometer aggregates. Three-dimensional ones were observed for sample **4**, where the less representative (4.5% by intensity) was made of very large micrometer particles. Anyway, considering only the acquisition by number (%) recorded at the highest kcps and the most represented dimension families (>99% for **2**, **3** and >91% for **4**), Z_AVE_ of particles of **2**, **3** and **4** were 604.9 ± 218.2, 156.9 ± 54.4 and 140.4 ± 22.9 nm, while their PDI were 0.370, 0.438 and 0.438 (Table 1).

The phenomenon of multi-dimensional families has been previously reported for a bola-amphiphilic QPS molecule [38,67], which could be a sign of a phenomenon like the well-known “bolalipid’s polymorphism” [67]. It is reported that the detected micrometer particles probably consisted of less soluble larger aggregates [67]. Nonetheless, filtration to remove larger aggregates was not possible, since such an operation would have excessively lowered the number of kcps, invalidating the analysis. All compounds demonstrated a positive ζ-p, but not particularly high (ζ-p = +4.2, +15.7 and +13.4 mV), for **2**, **3** and **4**, thus confirming their low solubility and tendency to aggregate in suspension. Conversely, compound **1** showed a ζ-p value decisively higher (+38.1 mV, Figure S24, Section S5, Supplementary Materials), thus confirming higher solubility, stability in suspension and higher possibility to possess membrane disruption effects. In fact, bacterial surfaces, like the surface of cancer cells, are generally negatively charged, due to the presence of lipopolysaccharides, teichoic acids, and anionic phospholipids. Hence, systems with a positive surface charge can exhibit a strong electrostatic attraction toward the bacterial membrane as well as that of cancer cells, which increases contact and adhesion [32,34,35]. This interaction can lead to surface charge neutralization, destabilization of the lipid bilayer, and increased membrane permeability, resulting in loss of cell integrity and death. It has been reported that a markedly positive ζ-p is associated with strong anticancer properties and broader-spectrum antibacterial efficacy, even at lower concentrations [68,69,70]. Nanovesicles with significantly high positive ζ-p (+18.33 mV) displayed high anticancer effect and a broad bacteriostatic activity. Conversely, other compounds with positive ζ-p up to +49.8 and +57.6 mV [38,68,69,70] also demonstrated a bactericidal behavior. Additionally, high positive values of ζ-p can help reduce BF formation [71], making these systems particularly attractive also for antimicrobial coatings and targeted drug delivery [32,34,35].

On these considerations, due to its high positive ζ-p = +38.1 mV, compound **1** could possess broad-spectrum, bactericidal and ABF properties.

### 3.2. Antibacterial Profiles of Compounds ***1**–**4***

#### 3.2.1. The Issue of Compounds **2**–**4** Solubility: Future Strategies to Solve the Mystery of Their Antibacterial Effects

As future perspective for reevaluating compounds **2**–**4**, it was thought to ameliorate their poor solubility, hampering the MIC determination, by inserting hydroxyl groups in their original structures, or doubling the cationic heads. Terekhova et al. demonstrated, in fact, the significant antibacterial activity against Gram-positive MDR *S. aureus* (SA1 and SA2) of ten diphenyl alkyl hydroxybenzyl phosphonium chlorides, having structures like those of **3** and **4**, but possessing hydroxyl groups, augmenting the solubility of compounds [54]. Reevaluating compounds **2**, **3** and **4** before their definitive rejection should be imperative, being that they are nano-dimensioned (605, 157 and 140 nm), morphologically suitable for clinical uses and positively charged, for promising antibacterial effects, by membrane disruption.

#### 3.2.2. Antibacterial Profile About Compound **1**: From Confuting Old Antibacterial Effects (MICs) to Further New Experiments

##### MICs Determinations

The behavior of **1** against *S. aureus*, *E. faecium* and *P. aeruginosa* was completely different and opposite to that reported by Kodjo et al. against ATCC strains of same species [27], while confirming that reported by other authors [50,53,54,72,73]. MICs = 8–32 µg/mL against four strains of *S. aureus* (3 MRSA), MIC = 64 µg/mL against four VRE *E. faecalis*, also resistant to teicoplanin and in some cases to linezolid, and MIC > 128 µg/mL against MDR *P. aeruginosa* were observed. These findings aligned with those previously reported by other authors for similar benzyl compounds [53,54,72,74] or compounds containing only one TPP group [37,75,76]. Usually, similar mono-cationic compounds were found active on Gram-positive species and not on Gram-negative ones, as in this case. At least, it has been reported that two TPP-cationic heads are necessary to have activity against Gram-negative species [38,75].

Anyway, Galkina et al. synthesized benzyl QPSs having two diphenyl benzyl cationic heads linked by a C2, C3, or C6-alkyl, which, despite possessing two phosphonium cations, were only poorly active against *E. coli* and *P. aeruginosa* [77]. On these considerations, we are confident that our double-checked results are correct and represent the actual antibacterial behavior of **1**. Collectively, despite inactivity of **1** against Gram-negative superbugs being confirmed, its antibacterial effects against all Gram-positive MDR clinical isolates used here are of paramount importance, since most belong to ESKAPE bacteria group [78], currently considered a major therapeutic challenge, despite the introduction of several new antibiotics and antibiotic adjuvants, such as novel β-lactamase inhibitors [78].

##### About Antibacterial Properties of TPP-Salts Previously Reported

The antibacterial activity of TPP-salts conjugated to ciprofloxacin by ester-linkages was reported by Kang et al. against VRE, MRSA and vancomycin intermediate *S. aureus* (VISA) [79]. In this regard, the antibacterial potency of **1** against VRE (also resistant to linezolid) and MRSA (also resistant to teicoplanin) was higher by 4 and 2–16-fold [79]. Furthermore, the antibacterial activity of **1** against MRSE, MRSA and *E. faecium* VRE (also resistant to teicoplanin and linezolid) was comparable to that of same TAPILs bromides and chlorides when tested against standard strains from the National Collection of Type Cultures (NCTC), London, and ATCC, including *S. epidermidis*, *S. aureus* and *E. faecium* [80]. Some TAPILs with chlorine counterions developed by Cieniecka-Rosłonkiewicz et al. showed MICs significantly higher than those of **1** (up to 125 µg/mL, compound **2g**) against NCTC *S. aureus* and ATCC *E. faecium* [80]. BAC is a commercially available mixture of quaternary ammonium salt (QAS), having an average molecular weight of 368.

##### The Case of BAC

BAC is known to be a potent biocide, which acts mainly in a not specific mode, as assumed for QPSs, as **1**, disrupting the bacterial cell membranes, denaturing proteins, and damaging the essential metabolic enzymes, leading to bacterial death [81,82]. Anyway, frequently, bacteria demonstrate heterogeneous susceptibility to BAC, due to both genetic factors (presence of qac efflux genes, 80%) and physiological status of bacteria (planktonic vs. BF cells, stationary vs. exponential phase), as well as several other factors [83]. BAC has been reported to have MICs = 4 µg/mL against NCTC 13143 *S. aureus* [84] and MICs = 2–10 or 5/5.1 µg/mL against MRSA, depending on different studies [85,86,87]. Rahami et al. reported that different gene mutations between different HA-MRSA strains affected the resistance of HA-MRSA to BAC [88]. Previous tests against hospital-acquired (HA)-MRSA, isolated from various hospitals in Rome (Italy), resulted in BAC having different MICs on the various HA-MRSA strains tested (2 µg/mL, 4 µg/mL, 8 µg/mL, and 16 µg/mL) [88]. In the research by Rahami et al., conducted to establish MICs of BAC against HA-MRSA by the colony counting method, MIC observed was 5 μg/mL [88]. Also, Akimitsu et al. and Rahmi et al. reported that MICs of BAC against parent MRSA and those of MRSA strains muted under administration of 5 µg/mL BAC were higher by 7.1–14.2-times with respect to MICs against MSSA [89]. Also, reported MBCs for BAC against *S. aureus* are different depending on the different studies, different conditions for determinations and the *S. aureus* type. Worthing et al. reported MBC values against MRSA in the range 33.7–135 µg/mL in the presence of bovine serum albumin (BSA), while they ranged from 2.1 to 16.9 µg/mL in the absence of BSA [85]. Cieniecka-Rosłonkiewicz et al. reported MBCs = 8 µg/mL against *S. aureus* NCTC 4163 [80]. On all these reports, BAC is anyway more potent than **1** [38], but also toxic for the environment and genotoxic at 1 µg/mL towards rats’ hepatocytes and human lymphocytes [90]. BAC selectivity index values (SIs) are in the range 0.06–0.5, which also limits its use as topical or environmental detergent or disinfectant. On the contrary, as better detailed later, selectivity of **1** for MRSA with respect to eukaryotic cell lines (human keratinocytes HaCaT and murine fibroblasts 3T3 cells), as well as red blood cells (RBCs), was extremely higher than that of BAC (SIs = 15.7–62.6, 47.4–189.5 and 1.7–6.6, respectively), thus establishing its potential suitability for clinical applications. Additionally, the emergence of bacteria resistance upon the use of BAC has been widely reported in vitro and is perceived to present as a high risk [83].

##### Structural Considerations

The structure of **1** presents four phenyl rings, three of which are directly linked to a phosphor atom and the fourth linked to a phosphor atom by a unique methylene, thus being strongly hindered (Scheme 1). Conversely, **3** and **4**, having only three rings, of which only two are directly linked to a phosphorous atom and an alkyl chain, are less hindered. Therefore, the cationic phosphor atoms in compounds **3** and **4** should have been more exposed than in **1** and possibly more likely to attract detrimentally, bacteria negatively charged envelop and inhibit them (Scheme 1). Anyway, the reported possibility to have inductive effects (major contributor) and resonance effects (minor contributor) in triphenyl phosphonium compounds as **1**, as well as in compounds as **3** and **4**, stabilize the phosphorous cationic charge for delocalization on phenyl rings (three in **1** and two in **3** and **4**) in three possible positions for each ring (*ortho*, *para* and *ortho* again) [30,67,91]. Specifically, phenyl groups can withdraw electron density through the σ-framework (–I), stabilizing the positive charge localized on phosphorus, while a small amount of “π-acceptor stabilization” could also occur, augmenting the overall effect [30,67,91]. The major result, important for antibacterial activity, is a significantly and progressively enlarged cationic radius [30]. In molecular orbital terms, delocalization of cationic charge on a phosphorous atom to the rings (one, two or three) spreads the positive charge over a larger volume.

Such phenomenon enables phenyl, *bis*-phenyl and at best TPP^+^ phosphonium cations to better electrostatically interact with bacteria surface and destroy it, thus contrasting their progressive steric hinders having opposite effects. This major cationic radius and stabilized cationic charge for delocalization on all phenyl rings can also justify why, usually, phenyl phosphonium derivatives show higher antibacterial potency [37,38,40,42,43,44] than those not containing phenyls, tetra alkyl QPSs [45] (Scheme S2A–C, Section S8, Supplementary Materials).

The possible delocalization of phosphorous cationic charge on three, rather than two phenyl rings (compounds **3** and **4**), existing for compound **1**, could justify the major zeta-potential value (>+38 mV) found for **1** with respect to those of **3** and **4**. On the other hand, the possible weak π-resonance also existing for **2**, where σ-inductive effects are inexistent because the phosphorus atom is not cationic, can justify the unexpectedly positive Z-potential observed for **2**, despite being uncharged (+4.2 mV). The delocalization of π-electrons from the rings to the phosphorous atom, while localizing an anionic charge on phosphorous, created positive charges delocalized in three positions (*o*, *p* and *o* again) on two phenyl rings, hence presenting large cationic radii, despite not being cationic (Scheme S3, Section S8, Supplementary Materials).

Anyway, if, in TPP-compounds, three phenyl groups linked directly to a phosphorous atom are important to give the highest possibility of having structures with enlarged cationic radii and stabilized for delocalization [30], the presence of four rings is too much, due to the extremely high steric hindrance on cationic active charge. In fact, it has been reported that extremely hindered tetra phenyl phosphonium salts have been found completely inactive against Gram-negative *E. coli* [92].

##### Time-Kill Experiments

The time-kill kinetics assay is used to study the activity of an antimicrobial agent against a bacterial strain and is mainly used to determine the bactericidal or bacteriostatic activity of an agent over time [93]. Additionally, time-kill data suggest a relationship between concentration, exposure time, and surviving fraction, which can be modeled probabilistically for editing infection control protocols [94]. As for Clinical & Laboratory Standards Institute (CLSI) guidelines and other laboratory-approved indications [93], bactericidal activity is then defined as greater than 3 log_10_-fold decrease in colony-forming units (surviving bacteria), which is equivalent to 99.9% killing of the inoculum. A growth control is included in the experiment as negative control [93]. The log CFU/mL for all tested samples should be determined at time 0 and at subsequent time points up to 24 h [70,95,96] since, after a rapid decrease in the initial inoculum, frequently, a rapid regrowth occurs in the following hours, due to an incomplete extermination of pathogens [97,98]. Anyway, it is difficult to find papers in which this indication is respected and, frequently, the shown analysis is stopped when >Log_10_ reduction is achieved (4–6 h) without showing if any regrowth occurs in the subsequent hours to 24 h [79]. In this regard, quaternary phosphonium (QP)-*N*-chloramine (compound **6** in the study) by Zhou et al., having a C12 alkyl chain, demonstrated the highest biocidal efficacy after 10 min of contact, causing a 6.22 and 7.30 CFU log reduction in the initial inoculum of *E. coli* and *S. aureus*, respectively [99]. Anyway, what could occur for longer contact times, up to 24 h, was not investigated or reported [99]. Conversely, **1** demonstrated bactericidal effects against *S. aureus* strains, as a decrease of >3 log_10_ occurred, within 11 h from inoculum (equivalent to 99.9% killing of the inoculum), while no regrowth was observed up to 24 h, when a 5 log_10_ reduction was reached. Collectively, **1** was capable of completely exterminating MRSA and MSSA ATCC *S. aureus* without difference in the killing profile. The interesting aspect of the behavior of **1** consists just in the absence of regrowth up to 24 h, instead observed for several other short-time biocide cationic compounds [97,98], thus establishing a complete extermination of pathogens.

A kinetic study was carried out by fitting the time-kill curves in Figure 2 with PFO and PSO mathematical models.

Despite PFO and PSO models being typically applied to data of adsorption over time in water decontamination studies [65,66], we have recently demonstrated and already published their successful applicability to data of removal efficiency loss (%) of biochar-based systems over time, which produce graphical profiles like those of time-kill curves [100,101]. Therefore, such simple mathematical models were herein fit to data of bacterial killing over time, to describe their kinetics, following the procedure described in Section 2.4.2. Evaluation of kinetic parameters by a model with experimental data confirmed the goodness of method, while K_PSO_ constants established that the killing rate was higher than the growth rate of untreated bacteria by 7.2-times.

The potent biocide BAC was considered again for comparison, together with other TPP-salts, QASs and ammonium polymers. Available time-kill experiments for BAC report different BAC concentrations. When 100 µg/mL (an intermediate concentration between that used for **1** against MRSA 189 and 18) BAC were administered, Blondeau et al. observed that BAC produced a >5-log kill reduction in MRSA colonies within 5 min and no regrowth up to 3 h exposure, but what occurred after 3 h was not investigated [102]. In this regard, BAC was several times more rapid in killing MRSA than **1**, which reached >5-log_10_ more slowly at 24 h treatment, without observing any regrowth. On the other hand, it was also reported that BAC at concentrations <5–8 µg/mL, anyway, at a toxic level for humans and environment (SI < 0.13–0.20), produces slow or incomplete killing. At concentrations substantially above the MIC/MBC values, the killing rate increases, but persister subpopulations can cause bimodal or multimodal time-kill curves [103], which were not observed with **1**. Rapid killing is achievable only at ≥10 × MIC, typically 150–300 µg/mL (SI = 0.006–0.003) for MRSA isolates. Sublethal exposure to BAC or inadequate contact time is very risky, since it can select for tolerant subpopulations, potentially facilitating cross-resistance to antibiotics used in humans. Therefore, despite MRSA being rapidly killed by BAC, with a 6-log_10_ reduction within ~1 h, but at genotoxic concentrations ≥150 μg/mL (SI < 0.006), **1** exterminated MRSA 18 and 189 (with >5-log_10_ reduction) significantly less rapidly, but at lower non-cytotoxic concentrations (128 and 64 µg/mL). Concerning reported synthetic molecules [43,79,104,105,106], the kinetic killing constant (K_PSO_) calculated for reported TPP-compounds and QASs, as well as two quaternary ammonium polymers (P5 and P7), whose time-kill data best fitted the PSO mathematical model, were compared with K_PSO_ of **1** and inserted in Table S4 (Section S9, Supplementary Materials).

Generally, TPP-compounds are reported to be slower biocides (3–5 log_10_ CFU/mL reduction within 8–24 h) than QASs and QPSs because they act by a not classical membrane disruption, but bactericidal effect appears associated with mitochondria-like membrane targeting and possibly interference with bacterial energetics, analogous to their mitochondrial targeting in eukaryotic cells. On the contrary, QASs and QAPs exert a very rapid bactericidal effect, often within 1–4 h at concentrations ≥ MBC, due to a mechanism based on an immediate perturbation of the cytoplasmic membrane, specifically leading to membrane disruption via cationic–lipid interactions that compromise bacterial cell wall integrity. These salts show time-kill curves having a steep decline in CFU/mL within the first 2–3 h, despite a plateau or regrowth possibly occurring if some bacteria survive at sublethal exposure. Although this possible behavior is not observable in curves by Xiao as well as Saseendran et al. [105,106], the possibility of its occurrence was confirmed in our study [69]. In this regard, **1**’s performance in killing MRSA was like that of reported TPP-compounds [43,79,104], while it was significantly lower than that of QAMSs and QAPs.

##### Antibiofilm Experiments: Inhibition of BF Formation

The bacterial biofilm (BBF) is an organized community of bacteria that adheres to a living or non-living surface and surrounds itself with a polymeric extracellular matrix (EPS), composed primarily of polysaccharides, proteins, lipids, and extracellular DNA (*e*DNA) [107]. Collectively, BBF is a sort of three-dimensional “microbial city” where microorganisms are numerous and cooperate with each other; protect each other with a physical and chemical barrier; and communicate through quorum sensing (QS), a signaling system that coordinates collective behavior [107]. Therefore, bacteria hindered in the BF and so well organized are difficult to be or are no longer reachable by antibiotics which fail, causing the widespread of severe or even intractable infections [107]. BFs are involved in over 60% of chronic wound infections, which can be colonized by a single or several bacterial species [108]. The major Gram-positive bacteria involved in BF formation are *S. aureus* and *S. epidermidis* [109,110]. The formation of BF by these bacteria represents the main virulence factor of microorganisms, reducing the effectiveness of antibiotics by up to 1000 times and host immunity [111], thus promoting chronic, persistent and recurrent infections (bacteremia and nosocomial sepsis, endocarditis (*S. aureus*), neonatal infections) associated with implanted or indwelling medical devices [109,110,112,113]. Venous and urinary catheters, orthopedic prostheses, heart valves, pacemakers, cerebrospinal shunts, and peritoneal dialysis devices represent the most typical and clinically relevant sites, where intractable and lethal biomaterial-associated infections (BAIs) can develop due to BF, thus worsening the patient’s condition, leading to the need for device removal [114,115]. In BAIs, the development of the infection depends on the type of implant and the length of time the implant is in the patient. The adhesion of pathogens to permanent medical devices is favored by fibronectin and fibrinogen, which act as adhesion mediators for staphylococci [116].

In this worrying scenario, there is an urgent need to develop innovative ABF strategies, specifically targeting the early adhesion and maturation processes of *S. aureus* and *S. epidermidis*, where current antimicrobial and anti-adhesive interventions frequently fail to effectively control these sessile communities.

To this end, compound **1** and vancomycin, selected as the reference antibiotic not tolerated by bacterial isolates used, were assayed in BF-inhibition experiments, carried out as described in Section 2.4.3, with results shown in Figure 3, Figure 4 and Figure 5 and in Figures S26 and S27 in Section S10 (Supplementary Materials). In the following discussion of results, *S. aureus* isolates were referred to using SA and related isolate numbers, while *S. epidermidis* using SE. On this premise, Stepanović (2007) and O’Toole (2011) et al. [117,118] proposed the classification of BF producers reported in Table S5 (Section S10, Supplementary Materials), according to which if an OD > 1.5–2.0 is observed, the isolate tested is a very strong BF producer. Among *S. aureus* and *S. epidermidis* BF producers herein considered, all were very strong BF producers, except for *S. aureus* 18 and 189, which were moderate. *S. aureus* B (OD_570_ = 2.6), *S. epidermidis* 22 (OD_570_ = 3.6) and *S. aureus* B (OD_570_ = 2.9) were the major BF producers (Figure 3). Despite this, when administered at 2.5 and 5 × MIC, **1** was capable of remarkably (*p* < 0.0001) reducing BF formation by all strains (Figure 3), lowering OD_570_ values to 0.1–0.2, without any statistically significant differences among 2.5 and 5 × MIC, except in the case of *S. epidermidis* 147 (OD_570_ at 2.5 × MIC = 0.6, OD_570_ at 5 × MIC = 0.2). Collectively, BF inhibition at these concentrations was of 76–95%, being, in several cases, the major inhibition caused by **1** administered at 2.5 × MIC, rather than that derived by 5 × MIC. However, in three cases out of eight, the simple MIC administration of **1** was capable of significantly inhibiting (*p* < 0.0001) BF formation by *S. aureus* ATCC 29213, as well as *S. epidermidis* 22 and 147, reducing their OD_570_ by 4, 7.2 and 1.25-fold, which means a BF inhibition percentage (%) of 78, 87, and 19%. A lower inhibition of 7% was caused by **1** in the BF production by *S. epidermidis* 64. To date, antimicrobial peptides (AMPs) represent the most promising agents in counteracting chronic infections caused by the BF produced by MDR superbugs [75]. New AMPs have been recently developed, which demonstrated potent broad-spectrum sterilizing activity against a panel of Gram-positive and Gram-negative BFs [75]. In this regard, Andreeva et al. developed a library of several TPP-conjugate compounds and investigated the potential ability of lead compounds for the demonstrated MICs and MBCs (**4d**, **4e**, **4f**, **5d**, **5e**, and **5f**), both to inhibit the formation and to destroy the mature BF by not MRSA *S. aureus*, using crystal violet (CV) dye. From the data shown by authors, which are only percentages, not showing the OD values of untreated and treated bacteria, it is impossible to understand if the BF producers they used are strong, mediocre or weak (which should be a mandatory data to correctly weight the observed inhibition); most of our results (%) concerning MRSA were like those of Andreeva et al. Specifically, except for compound **4c**, which at MIC inhibited BF formation by almost 100%, four compounds by authors caused inhibition <30% and compound **4f** inhibited the BF by about 85% [75]. Collectively, administration of 4–6 × MIC was necessary for all compounds by Andreeva et al., to reach an inhibition in the range 80–100%. Here, **1** at MIC reduced *S. aureus* ATCC 29213 by 78%, while at 2.5–5 × MIC reduced BF formation by 83–97%. Additionally, **1** caused the 87, 7 and 19% reduction in BF produced by *S. epidermidis* 22, 64 and 147, respectively, at MIC. The same authors similarly reported the inhibition (%) of formation of BF by not MRSA *S. aureus* caused by two lead compounds (**4j** and **4m**) of another library of TPP-conjugates [76]. At concentrations of 2 × MIC (**4j**) and 4 × MIC (**4m**), the inhibition of *S. aureus* BF was more than 80% [76]. Further, **1** caused the 87% BF inhibition (ATCC) at MIC and the 83–96% inhibition at 2.5 × MIC, thus outperforming both **4j** and **4m**. Both **1** and vancomycin, when administered at MIC concentration, did not inhibit BF formed by *S. epidermidis* 25 and 22. Vancomycin, on the contrary to **1**, inhibited BF formation by *S. aureus* 189 and AB, while **1** was a better performant than vancomycin in inhibiting BF produced by *S. aureus* ATCC 29213, *S. epidermidis* 22, 64 and 147. Both vancomycin and **1** remarkably inhibited BF formation by all bacteria tested at 2.5 and 5 × MIC. Collectively, the trend observed was that despite all bacteria being sensitive to vancomycin, this antibiotic functioned better than **1** against *S. aureus*, while **1** performed better against *S. epidermidis*. As for the most recent papers on BF inhibition, to investigate the intimate molecular mechanism supporting the capability of **1** to inhibit BF by *Staphylococci* used here was not in the scope of this already articulated study. Notably, such additional experiments would require very expensive instrumentation and highly equipped core facilities and laboratories (BSL 2) [119,120,121,122,123,124,125]. Anyway, based on the literature reports, it can be assumed that its ABF capacity could depend on its high positive ζ-p of +38 [71]. Moreover, the use of vancomycin as the reference antibiotic has evidenced that both vancomycin and **1** did not function at sub-MIC values (not reported results), requiring, in most cases, concentrations over the MIC to inhibit BF formation. Therefore, it could be rational to hypothesize for **1**, in relation to its ABF properties, a molecular mechanism like that of vancomycin. In this regard, it has been reported that, despite it being rather complex, the ABF mechanism of vancomycin is based on few aspects. Vancomycin binds to peptidoglycan precursors, specifically to dipeptides D-ala-D-ala, in the reachable outer layers of young BF [126,127,128], thus preventing wall elongation [129,130]. Anyway, vancomycin, being a relatively large molecule, as **1**, has difficulty penetrating the BF’s polysaccharide matrix, thus functioning only at high concentrations (as observed) [131,132] or when the BF is still young [128,133,134], thus weakening the overall structure, reducing bacterial adhesion to the substrate (catheters, implants) and making the bacteria more vulnerable to other agents, such as gentamicin [135]. Delivering vancomycin by proper carriers can enhance its ABF properties [136,137]. Several studies show that vancomycin is most effective before the BF is fully mature, by preventing bacterial division, impeding the production of the extracellular matrix and reducing the bacteria’s ability to consolidate on the surface [133,138]. Assuming for **1** a similar behavior, it can also be assumed that, as for vancomycin, for already developed BFs, combinations with other antibiotics or alternative strategies (enzymes, matrix-disrupting agents, etc.) will be necessary [139,140].

### 3.3. Cytotoxicity Experiments

As observable in Figure S28, viability of both 3T3 and HaCaT cell populations was close to 100% and over, up to the high concentration tested. Specifically, no significant difference in viability of 3T3 cells (%) versus control existed at all concentrations tested, while the significant differences in viability of HaCaT cells (%) versus control were detected at 0.4, 4.3 and 22 µg/mL regarding their proliferation. Selectivity of **1** for bacteria in relation to its cytotoxicity against HaCaT and 3T3 cells was assessed as described in Section 2.5. Selectivity index values (SIs) are essential to predict the therapeutic potential and possible clinical translation of a new compound, which revealed certain antibacterial effects.

According to data reported in Table S7 and Figure 6, **1** was safe for both HaCaT and 3T3 (SI = 3.4–27.1 and 10.3–82.1) cells. Specifically, **1** was significantly safer and more indicative for treating infections by MRSA (SI = 20.5–82.1 and 6.8–27.1), rather than those by MRSE (SI = 3.4) and VRE (SI = 10.3) clinical isolates. Cytotoxicity observed for **1** is lower than that observed for several TPP^+^-containing QPSs already reported [44]. Recently, Nunes et al. synthesized a series of quaternary heteronym salts, including seven QPSs (**1a**–**1g**) [44], and essayed them against MRSA and human HEK293 cells [49]. Except for the less cytotoxic compound **1b** (SI > 128), all compounds by Nunes were more cytotoxic versus HEK293 in relation to their activity against ATCC MRSA than **1** versus 3T3 cells in relation to its activity against all MRSA clinical isolates used here by 2.9–2625-times [44]. In a previous study, Nunes tested their compounds **1a**–**1g** against *S. aureus* CECT 976, sensitive to erythromycin, tetracycline, ciprofloxacin, and ampicillin, observing from the lowest MIC value, MICs = 2, 4, 8, 32 and >64 µg/mL [43]. Subsequently, the better performant compound **1e** was essayed against strains found resistant to tetracycline, ciprofloxacin and erythromycin [43]. MICs of **1e** were 1, 2 and 1 µg/mL, while its IC_50_ against hepatocytes HepG2 was about 5.4 µg/mL, thus establishing SI values of 5.5, 2.25 and 5.5. Therefore, selectivity of **1** for MRSA and *S. aureus* ATCC 20213 in relation to its cytotoxicity to eukaryotic cells was higher than that of **1e** for MRSA XU212, SA1199B and MSSA RN4220 by 1.2–29.8-times.

## 4. Materials and Methods

### 4.1. Compounds ***1**–**4***

Compounds **1**, **3** and **4** were in the form of quaternized phosphonium salts (QPSs). They encompassed the triphenyl benzyl (**1**) or the diphenyl-benzyl alkyl (**3** and **4**) phosphonium groups. The synthetic procedure to synthesize them and the phosphine compound **2**, as well as methods followed to carry out their complete characterization, are detailed in a previous paper [26].

### 4.2. Optical Microscopy

The experimental details of optical microscopy analyses are reported in our recently published paper [26].

### 4.3. Dynamic Light Scattering Analysis (DLS)

The mean diameter (Z-average), polydispersity index (PDI), and zeta potential (ζ-p) of compounds **2**, **3** and **4**, as well as the ζ-p of **1**, were measured at 25 °C using a Malvern Nano ZS90 light scattering apparatus (Malvern Instruments Ltd., Worcestershire, UK) at a scattering angle of 90°, as previously described [141]. The apparent equivalent hydrodynamic radii of samples were calculated using the Stokes–Einstein equation. Since results showed more than one-dimensional family, analyses were acquired both by intensity and number (%). Results by number (%) were presented and discussed. Aliquots of compound dispersions were withdrawn, having a count rate (kcps) sufficient for the analysis. The results from these experiments were expressed as mean ± SD of three measurements of ten runs per sample.

### 4.4. Microbiologic Experiments

#### 4.4.1. Clinically Relevant Superbugs Used in This Study

A total of twenty-three strains belonging to a collection of MDR Gram-positive and Gram-negative species of the University of Genova, kindly gifted by S. Martino Hospital for research, were used in this study. All were isolated from human specimens for diagnosis purposes and identified using VITEKR 2 (Biomerieux, Firenze, Italy) or matrix-assisted laser desorption-ionization time-of-flight (MALDI-TOF) mass spectrometric technique (Biomerieux, Firenze, Italy). The isolates included sixteen Gram-positive and seven Gram-negative bacteria of different genera. Among bacteria of Gram-positive species, eight were enterococci (four *E. faecalis* and four *E. faecium*), while eight were staphylococci (four *S. aureus* and fours *S. epidermidis*). All enterococci were MDR isolates variously resistant to vancomycin or to teicoplanin (VRE), while all staphylococci, besides the *S. aureus* ATCC 29213 strain, were MDR strains with resistance to methicillin (MRSA and MRSE) and, in some cases, also to linezolid. Gram-negative species included five non-fermenting strains of *P. aeruginosa* isolated from patients with cystic fibrosis with resistance to carbapenem and two strains of *Enterobacteriaceae*, consisting of one *E. coli* and one *K. pneumoniae* that were resistant to carbapenems by producing class A *K. pneumoniae* carbapenemase.

#### 4.4.2. Determination of MICs

To investigate the antibacterial activity of compounds **1**–**4** on the described pathogens, their minimal inhibitory concentrations (MICs) were determined by following the microdilution procedure detailed by the European Committee on Antimicrobial Susceptibility Testing (EUCAST) [142] and reported in our previous works [37].

#### 4.4.3. Time-Kill Curves

Time-kill curve assays for compound **1** were performed on three representative isolates of *S. aureus* (strains 18 (MRSA), 189 (MRSA) and ATCC 29213) as previously reported [69,143,144]. A mid-logarithmic phase culture was diluted in Mueller–Hinton (MH) broth (Merck, Darmstadt, Germany) (10 mL) containing 4 × MIC of the selected compounds to give a final inoculum of 1.0 × 10^5^ CFU/mL. The same inoculum was added to separate MH tubes as a growth control. Samples were incubated at 37 °C with constant shaking for 24 h. Aliquots of 0.20 mL from each tube were removed at 0, 1, 2, 4, 6, and 24 h, diluted appropriately with a 0.9% sodium chloride solution to avoid carryover of samples being tested, plated onto MH plates, and incubated for 24 h at 37 °C. Growth controls were run in parallel. The percentage of surviving bacterial cells was determined for each sampling time by comparing colony counts with those of standard dilutions of the growth control. Results have been expressed as log_10_ values of viable cell numbers (CFU/mL) of surviving bacterial cells over a 24 h period. A bactericidal effect was defined as a 3 log_10_ decrease of CFU/mL (99.9% killing) of the initial inoculum. All time-kill curve experiments were performed in triplicate.

#### 4.4.4. Detection of BF Production Using the Microliter Plate Method

BF production was detected using the microliter plate method and quantified spectrophotometrically using a method based on that reported by Crémet et al. [145]. To produce BFs, stationary-phase bacterial cultures of four *S. aureus* and *S. epidermidis* strains, known to be higher producers of BF (optical density at 570 nm, OD_570_ = 0.73–3.36), were diluted 1:100 aseptically to the wells of a 96-well polystyrene tissue culture plate (Corning, Milan, Italy) containing tryptic soy broth medium supplemented with 0.25% glucose and were incubated at 37 °C for 24 h. To evaluate the effect of compound **1** and vancomycin, used as the reference antibiotic, on BF synthesis, each compound was added to the growth medium at selected concentrations (MIC, 2.5 and 5 × MIC). After 24 h of exposure, media were discarded and each well was washed three times with phosphate-buffered saline to remove non-adherent cells. Plates were air-dried in an inverted position. Adherent microorganisms were stained with 0.1% crystal violet (CV) *w*/*v* in water. Excess stains were rinsed off with running tap water and the plates were air-dried. Adherent bacterial films were quantified spectrophotometrically by determining the OD_570_. Each isolate was tested in triplicate. The results were derived from three separate experiments and OD_570_ values were expressed as mean ± standard deviation (S.D.). The OD_570_ value obtained for each strain without any added compound was used as the control (CTR). The percentages of residual BF formed in the presence of different concentrations of **1** and vancomycin were calculated employing the ratio between the values of OD_570_ with and without the compounds, adopting the following formula: [(OD_570_ with drug/OD_570_ without drug) × 100]. The BF percentage inhibition by two compounds was also calculated using the formula 100 − [(OD_570_ with drug/OD_570_ without drug) × 100].

### 4.5. Cytotoxicity of Compound ***1*** on Eukaryotic Cells

Cytotoxic effects of **1** were essayed for 24 h on human keratinocytes (HaCaT) and murine embryonic fibroblasts (3T3) as described in our recent paper [26].

### 4.6. Statistical Analysis

Statistical significance was obtained using GraphPad PRISM software 8.0.1 by the analysis of variance (Two-way ANOVA) corrected for multiple comparisons using statistical Tukey hypothesis testing. The statistical difference of multiple comparisons was reported for each isolate, for each concentration and for each compound tested using symbols. Symbols used have been specified in this text and in Supplementary Materials (Section S6). Specifically, adjusted *p* values for multiplicity comparisons were reported for each comparison. No symbol was reported when *p* > 0.5. One symbol was used for *p* < 0.1, two, *p* < 0.01, three, *p* < 0.001 and four, *p* < 0.0001.

## 5. Conclusions

With this study, we have revolutionized, updated and completed the microbiologic, hemolytic and cytotoxic behavior of triphenyl benzyl phosphonium bromide (**1**), previously essayed by other authors exclusively to assess its MICs, with questionable results, since opposite to our findings, and in contrast with existing data for similar compounds. Compound **1** attracted our attention, first because it is a QPS compound, with already reported anticancer effects, and so is probably endowed with antibacterial properties. In fact, it is universally recognized that QPSs exert both antibacterial and antitumor effects, due to their positive charge delocalized on aromatic rings and lipophilicity. On bacteria, lipophilic cationic charge allows TPPs^+^ QPSs to strongly adhere by electrostatic interactions to their internal membrane, having a very negative ΔΨ, significantly higher than that of eukaryotic cells, thus concentrating selectively, up to 100–500× compared to the extracellular environment and human cells. Upon adhesion, they can cause the collapse of membrane potential and integrity, irreversible membrane destabilization and pore formation. Depending on their structure and the presence of proper alkyl chains, they can also enter bacteria cells, triggering oxidative stress (ROS, lipids and protein peroxidation), apoptosis-like mechanisms, and energy breakdown (ATP↓), leading to cell death. Repeated experiments confirmed that **1** is fully inactive (MICs > 128 µg/mL) against Gram-negative species, including *P. aeruginosa*, *E. coli* and *K. pneumoniae*, mainly due to the presence of the additional outer membrane, which is unique to Gram-negative bacteria. It contains lipopolysaccharide (LPS), which is extremely impermeable to hydrophobic and highly charged molecules, and functions as a chemical barrier that prevents the entry of antibiotics, antimicrobial peptides, and disinfectants. Collectively, Gram-negative bacteria are more difficult to kill because they combine physical barriers (LPS), chemical barriers, and active defense mechanisms, and also include a thinner peptidoglycan, selective porins limiting entries, efflux pumps active in expulsions, periplasmic enzymes for antibiotic degradation, etc., thus resulting in a multi-layered fortress. Despite Gram-positive bacteria having a simpler and more vulnerable structure, the emergence of MDR and biofilm-producing phenotypes has invalidated the effectiveness of most available antibiotics, making their infections lethal.

In this context, **1** represents a new, very promising bactericidal weapon to counteract Gram-positive MRSA, MRSE and VRE (*E. faecium* and *E. faecalis*). MICs in the range 8–64 µg/mL were determined for **1**, which was, for the first time, discovered to be bactericidal against MRSA clinical isolates and MSSA ATCC 29213 strains, within 11 h from inoculum, without any regrowth in the subsequent hours up to 24 h. A >5 log_10_ reduction in the initial inoculum was observed after 24 h exposure, at the end of the experiment. Additionally, for the first time, **1** was essayed in ABF experiments, which established its capability to fully inhibit BF formation by eight Staphylococcal clinical isolates among the strongest BF producers of our collection (OD_570_ up to >3), at concentrations depending on strains. Experiments on RBCs, as well as on HaCaT and 3T3 cells, found **1** softly hemolytic and non-cytotoxic versus eukaryotic cells, different from the most very promising antibacterial QPSs recently reported. Collectively, these in vitro results pave the way for further experiments in vivo using **1** on proper animal models, for their confirmation and translation to clinical experimentations, in sight of a future development of **1** as a novel antibacterial therapeutic to treat infections sustained by both planktonic and sessile MDR Gram-positive superbugs.

## Figures and Tables

**Table 1:** Average hydrodynamic diameter (Z_AVE_, nm) obtained by records acquired by number (%) at the highest kcps; DPI and average zeta-potential (ζ-p, mV) obtained by records acquired at the highest kcps.

Sample	Z_AVE_ (nm), kcps	PDI	ζ-p (mV), kcps
1	N.D.	N.D.	38.10 ± 6.15, 143.2.1
2	604.9 ± 218.2, 12.7	0.370	4.19 ± 3.26, 1.1
3	156.9 ± 54.4, 14.2	0.438	15.7 ± 5.07, 31.9
4	140.4 ± 22.9, 24.2	0.473	13.4 ± 7.63, 93.8

N.D. = Not determined.

**Table 2:** MICs of **1** against 16 MDR clinical isolates of Gram-positive species and 5 *P. aeruginosa* obtained from experiments conducted at least in triplicate. The modal value has been expressed as µg/mL.

Compounds	1 (433.3) ^1^	Oxacillin	Vancomycin/Teicoplanin
Gram-Positive Strains	MIC (µg/mL)	MIC (µg/mL)	MIC (µg/mL)
*S. aureus* 18 ^MRSA^	32	512	0.5 ^(V)^
*S. aureus* ATCC 29213 ^MSSA^	32	128	0.5 ^(V)^
*S. aureus* 189 ^MRSA^	16	0.5	0.5 ^(V)^
*S. aureus* B ^MRSA^	8	512	0.25 ^(V)^
*S. epidermidis* 22 ^MRSE^	16	128	0.5 ^(V)^
*S. epidermidis* 25 ^MRSE^	64	256	0.5 ^(V)^
*S. epidermidis* 64 ^MRSE^	64	128	0.5 ^(V)^
*S. epidermidis* 147 ^MRSE^	64	64	1 ^(V)^
*E. faecalis* 1 ^VRE,^*^,^**	64	N. R.	256 ^(V)^; 64 ^(T)^
*E. faecalis* 439 ^VRE,^*	64	N. R.	256 ^(V)^; 64 ^(T)^
*E. faecalis* 365 ^VRE,^*^,^**	64	N. R.	32 ^(V)^; 1 ^(T)^
*E. faecalis* 451 ^VRE,^*	64	N. R.	128 ^(V)^; 32 ^(T)^
*E. faecium* 152 ^VRE,^*^,^**	64	N. R.	128 ^(V)^; 64 ^(T)^
*E. faecium* 183 ^VRE,^*^,^**	64	N. R.	256 ^(V)^; 64 ^(T)^
*E. faecium* 185 ^VRE,^*^,^**	64	N. R.	256 ^(V)^; 32 ^(T)^
*E. faecium* 364 ^VRE,^*	64	N. R.	64 ^(V)^; 005 ^(T)^
**Gram-negative Strains**	**MIC (µg/mL)**	**MIC (µg/mL)**	**MIC (µg/mL)**
*P. aeruginosa* 259 ***^,CF,CR^	>128	>128	N. R.
*P. aeruginosa* 229 ***^,CF^	>128	>128	N. R.
*P. aeruginosa* 247 ***	>128	>128	N. R.
*P. aeruginosa* 256 ***	>128	>128	N. R.
*P. aeruginosa* 268 ***	>128	>128	N. R.

^1^ MW of compounds; MSSA = methicillin- (oxacillin) sensitive; VRE = vancomycin-resistant enterococci; MRSA = methicillin-resistant *S. aureus*; MRSE = methicillin-resistant *S. epidermidis*; * denotes resistance also to teicoplanin; ** denotes resistance also to linezolid; *** indicates resistance to carbapenems; CF = from patients with cystic fibrosis; CR = resistant to colistin; N.R. = not reported. All *P. aeruginosa* were MDR-resistant; V = vancomycin; T = teicoplanin.

## Data Availability

The original contributions presented in this study are included in the article/Supplementary Materials. Further inquiries can be directed to the corresponding authors.

## Supplementary Materials

The following supporting information can be downloaded at: https://www.mdpi.com/article/10.3390/ph19060829/s1, The supporting information to this article has been provided as Supplementary Materials file including Sections S1–S12.
