# Supplementary material for: Non-Cytotoxic Benzyl Triphenyl Phosphonium Bromide Is Bactericidal on MRSA and Fully Inhibits Biofilm Formation by MRSA and MRSE
Source: Pharmaceuticals (Basel). 2026 May 26;19(6):829. doi: 10.3390/ph19060829 (PMC13306021; doi:10.3390/ph19060829)
Supplement: Supplementary file 1 [file pharmaceuticals-19-00829-s001.zip › pharmaceuticals-4321386-supplementary.pdf]

pharmaceuticals

MDPI

Supplementary Materials

Non-Cytotoxic Benzyl Triphenyl Phosphonium Bromide Is Bactericidal on MRSA and Fully Inhibits Biofilm Formation by MRSA and MRSE

Silvana Alfei [1,*], Gabriella Piatti [2], Guendalina Zuccari [1,3,*], Caterina Reggio [3] and Anna Maria Schito [2,*]

[1] Department of Pharmacy (DIFAR), University of Genoa, Viale Cembrano, 4, 16148 Genoa, Italy, guendalina.zuccari@unige.it
[2] Department of Surgical Sciences and Integrated Diagnostics (DISC), University of Genoa, Viale Benedetto XV, 6, 16132 Genova, Italy, gabriella.piatti@unige.it
[3] Laboratory of Experimental Therapies in Oncology, IRCCS Istituto Giannina Gaslini, Via G. Gaslini 5, 16147 Genoa, Italy; caterinareggio@gaslini.org (C.R.)
* Correspondence: alfei@difar.unige.it; Tel.: +39 010 355 2296 (S.A.); amschito@unige.it (A.M.S.)

Section S1. Synthesis of Compounds **1-4**.

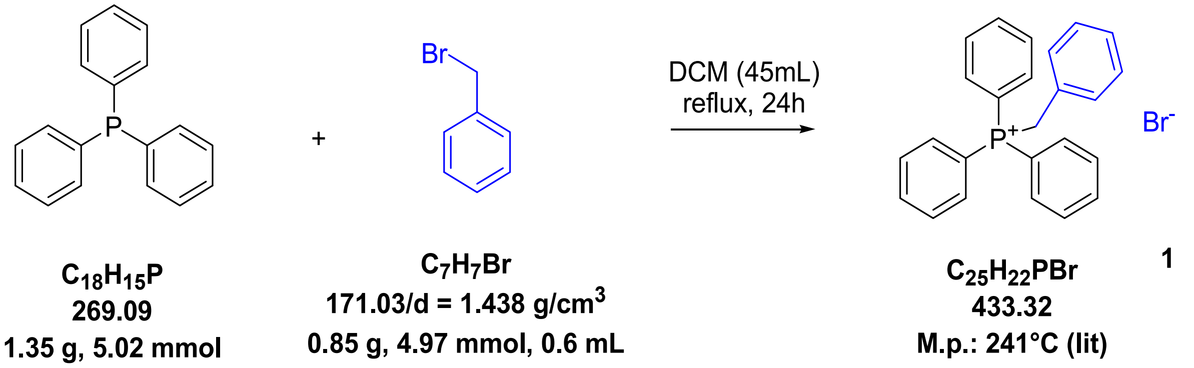

(A)

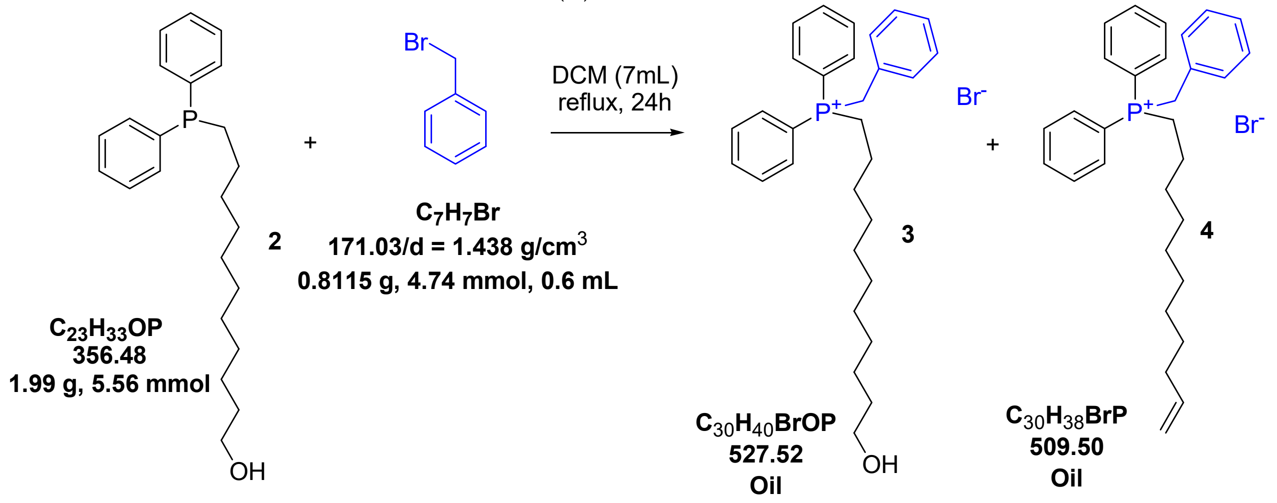

(B)

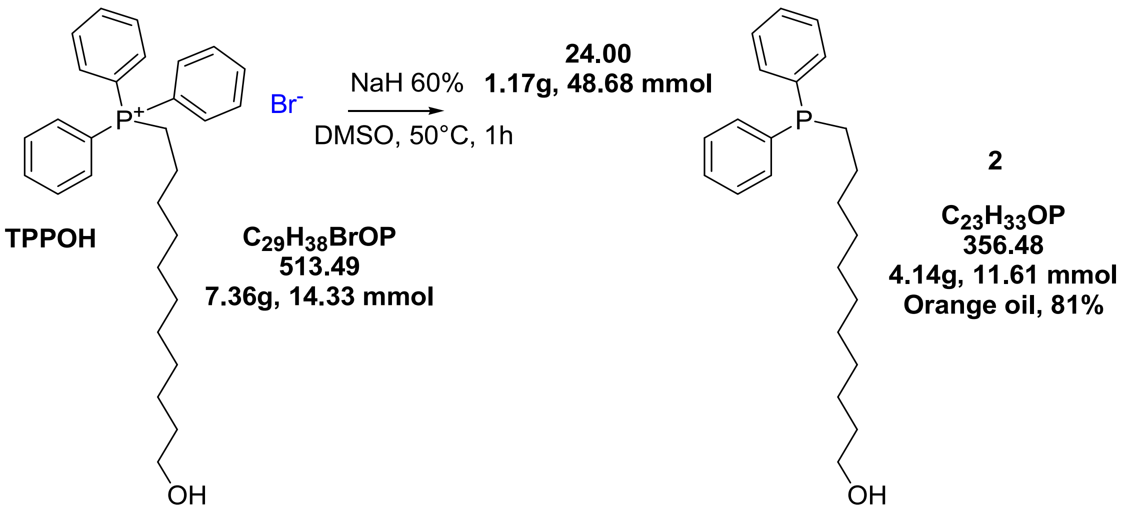

(C)

Scheme S1A–C. (A) Synthetic procedure to prepare compound **1** [63]; (B) synthetic procedure to achieve compounds **3** and **4**; (C) synthetic procedure followed to prepare precursor **2**. DCM = dichloromethane.

Section S2. Copies of ^{1}H, ^{13}C NMR and ^{31}P NMR spectra of Most Significant Compounds of This Study

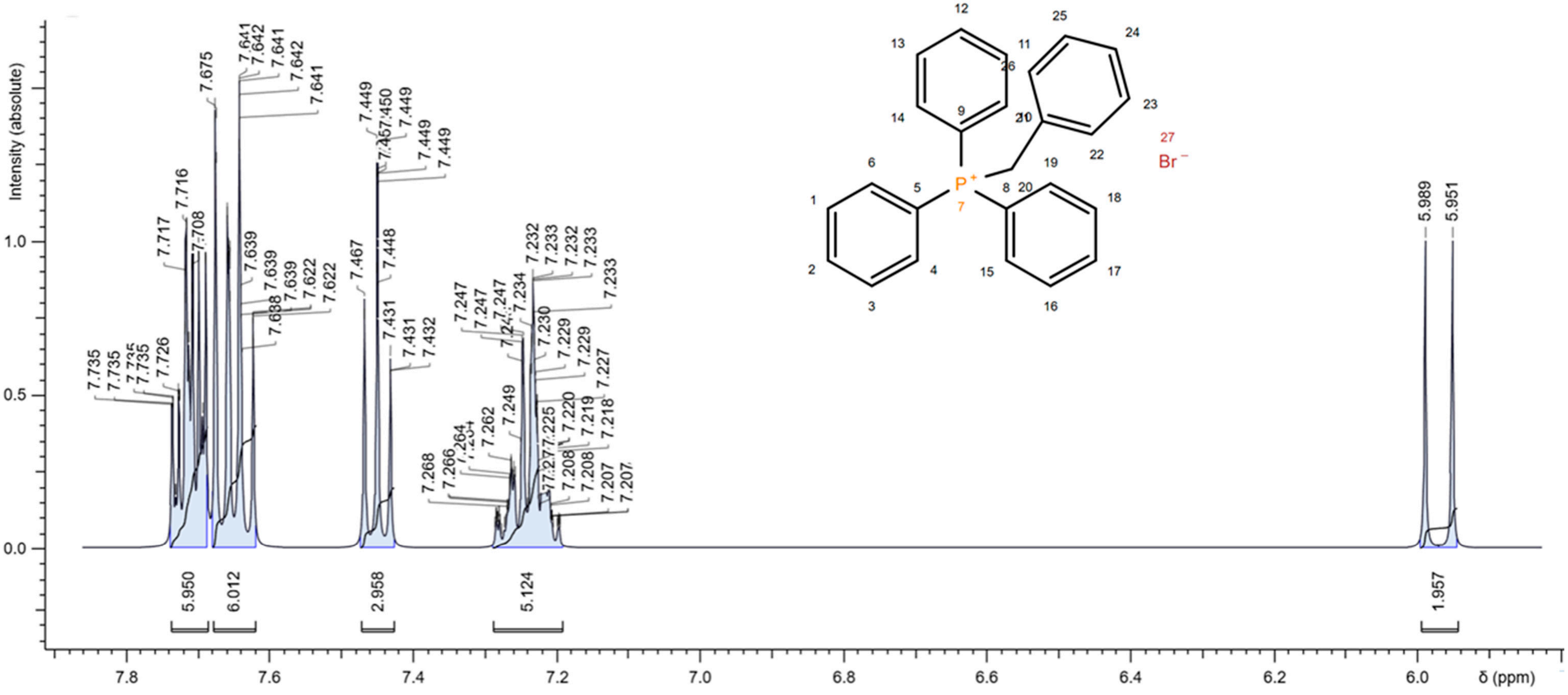

Figure S1. ^{1}H NMR spectrum (400 MHz, $CHCl_3$) of compound **1**.

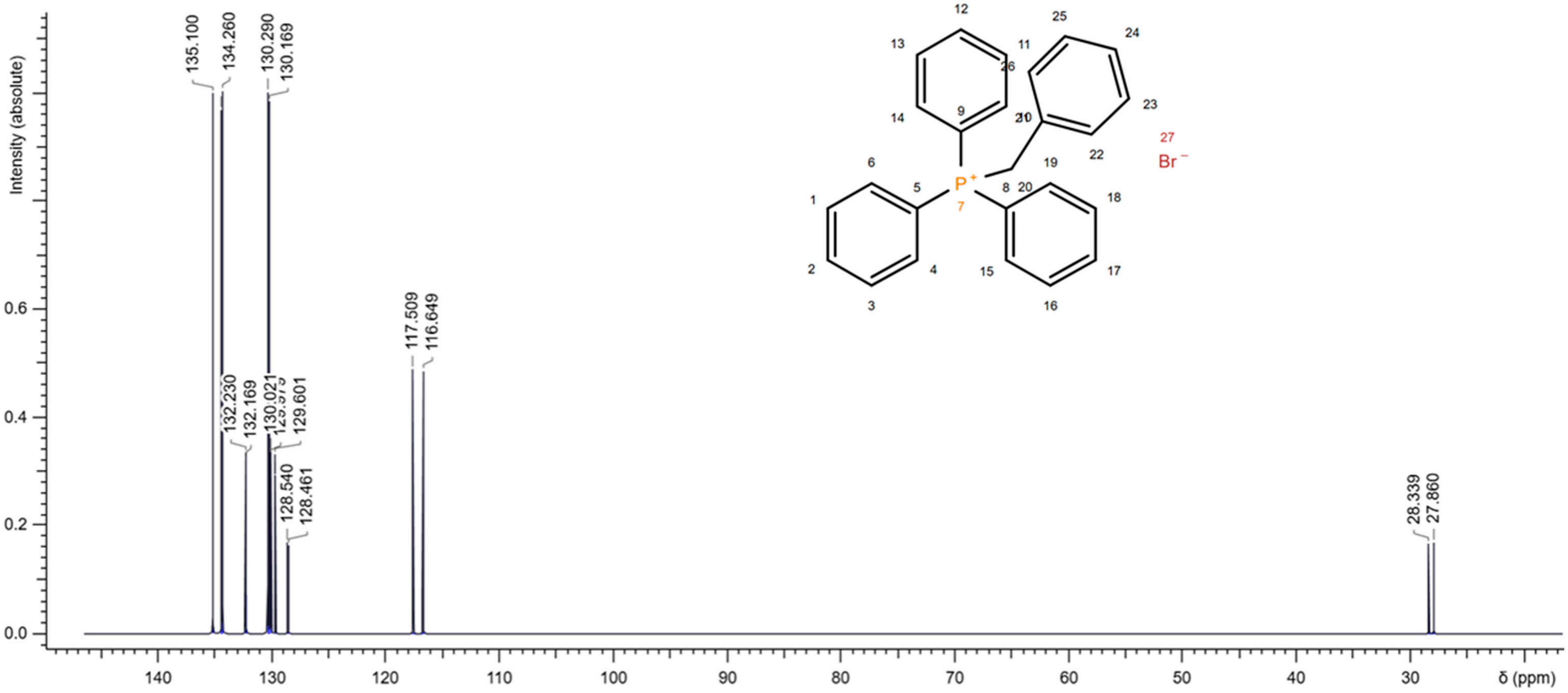

Figure S2. ^{13}C NMR spectrum (100 MHz, $CHCl_3$) of compound **1**.

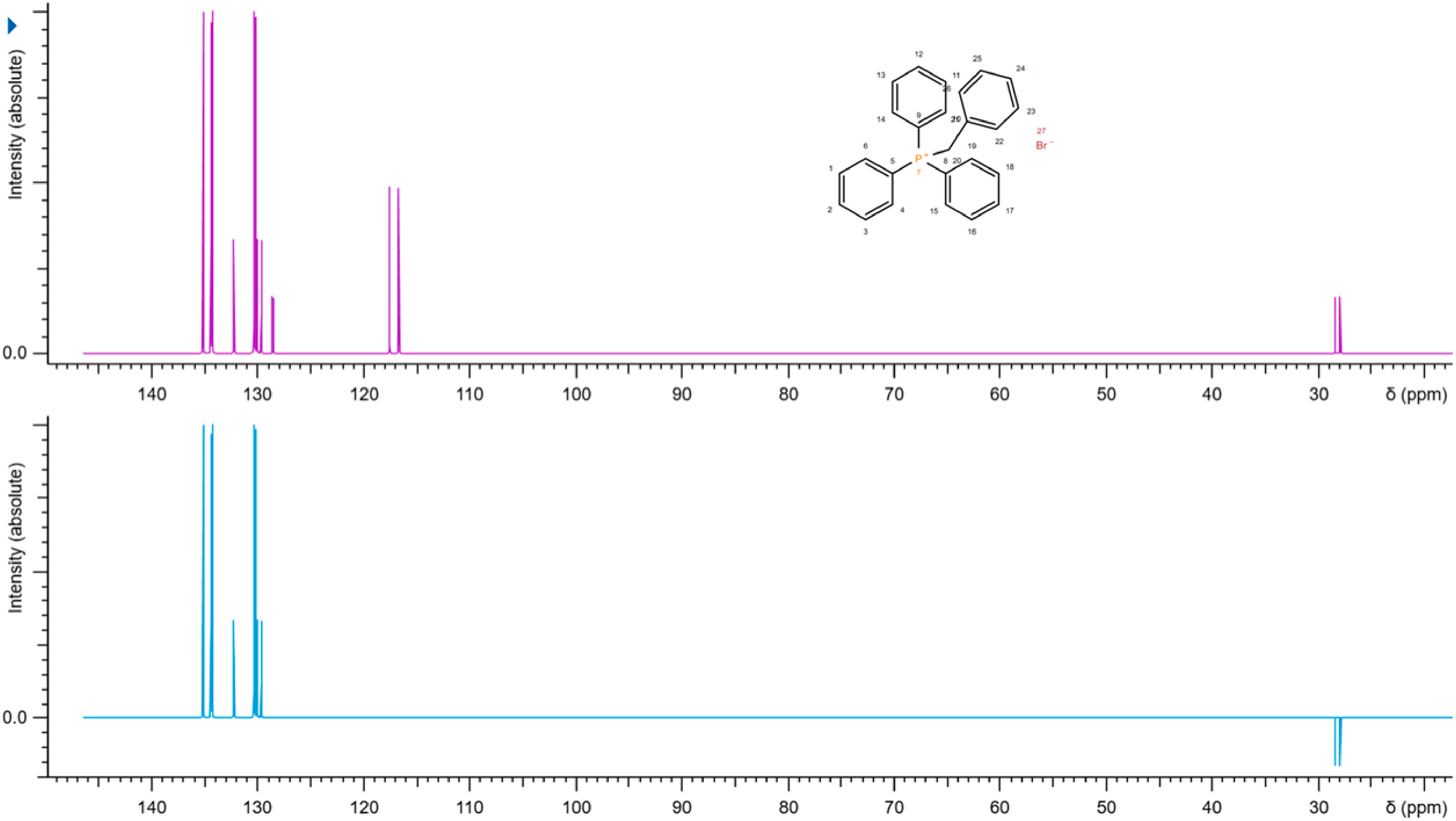

Figure S3. ^{13}C NMR spectrum (100 MHz, $CHCl_3$) and DEPT135 experiment of compound **1**.

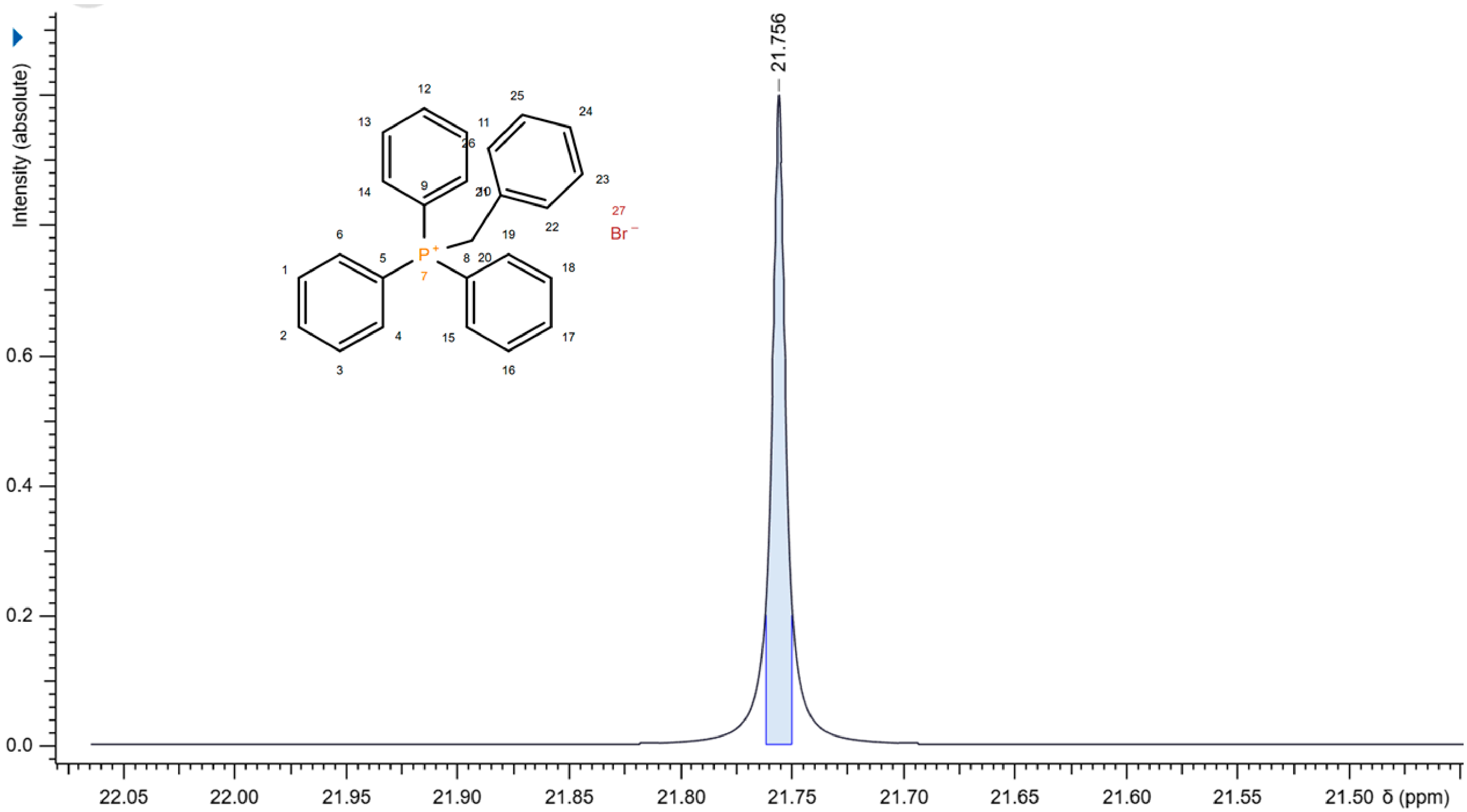

Figure S4. ^{31}P NMR spectrum (161 MHz, $CHCl_3$) of compound **1**.

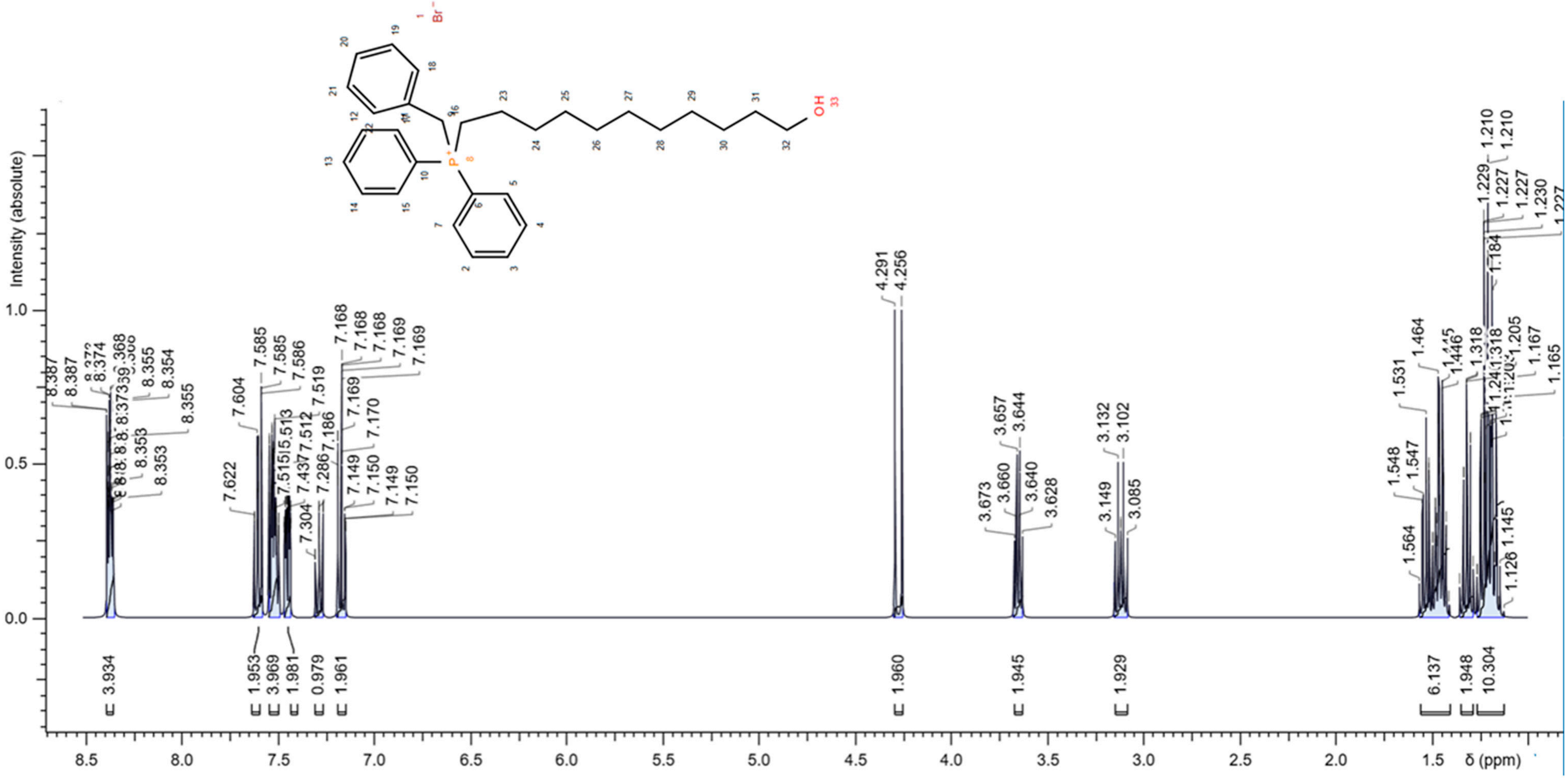

Figure S5. ^{1}H NMR spectrum (400 MHz, $CHCl_3$) of compound **2**.

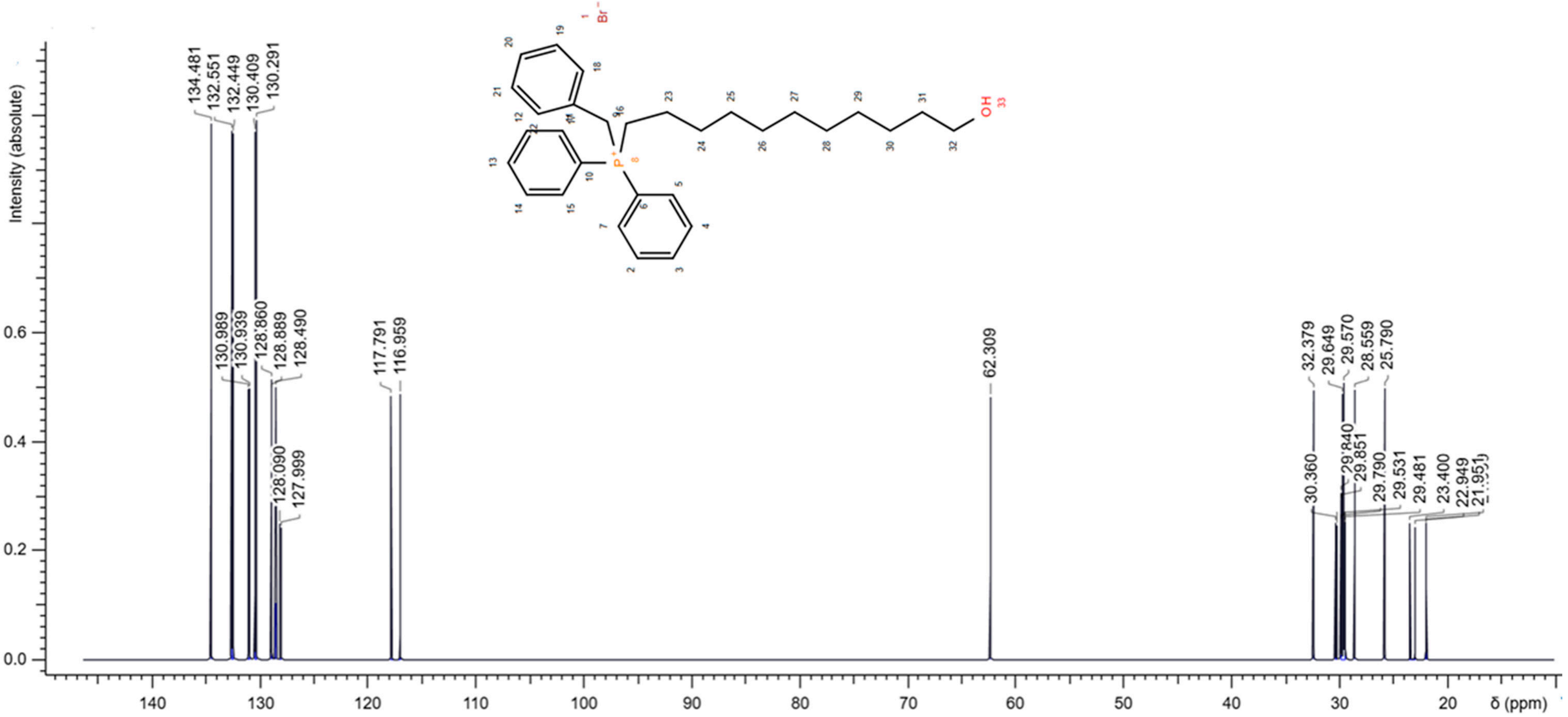

Figure S6. ^{13}C NMR spectrum (100 MHz, $CHCl_3$) of compound **2**.

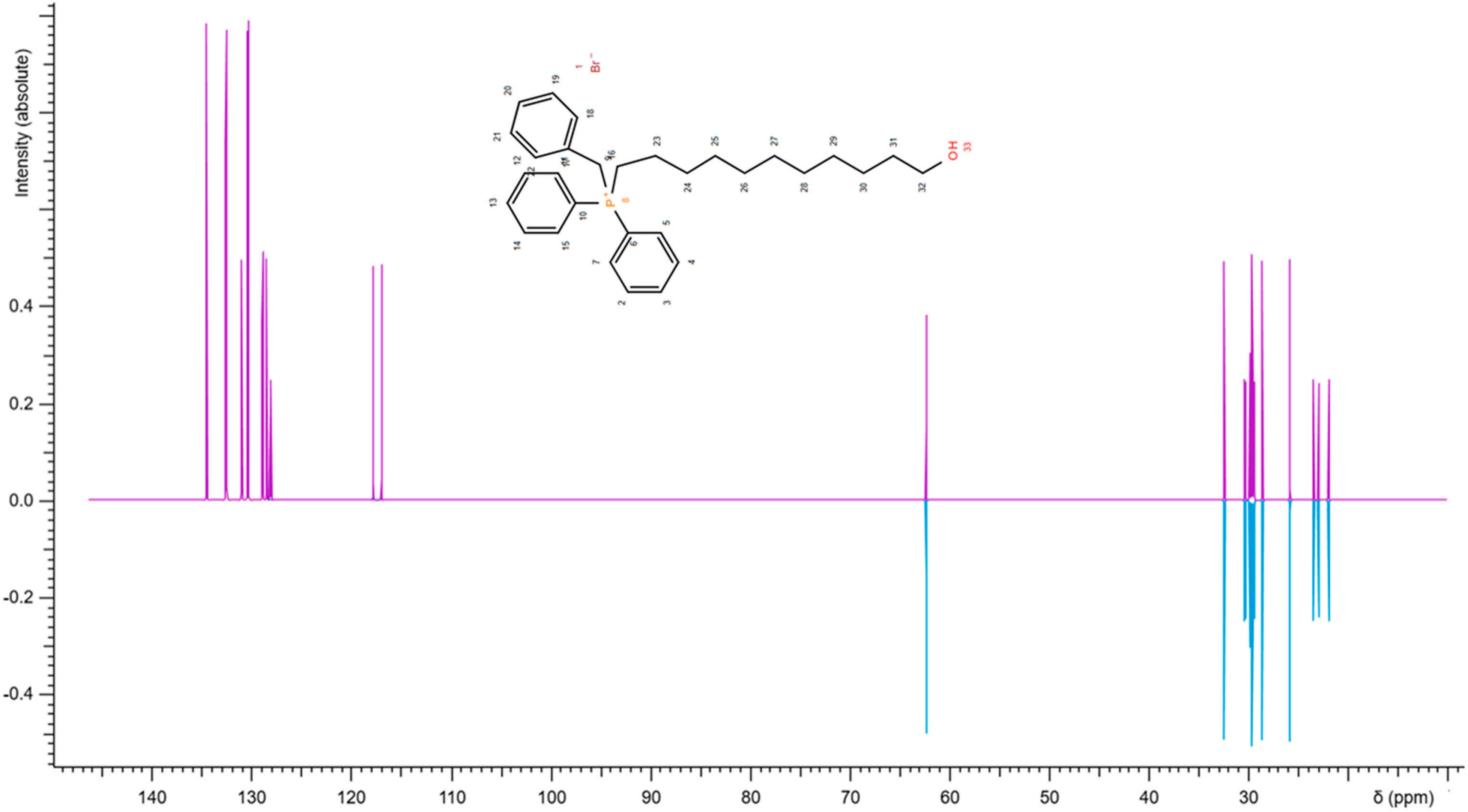

Figure S7. ^{13}C NMR spectrum (100 MHz, $CHCl_3$) and DEPT135 experiment of compound **2**.

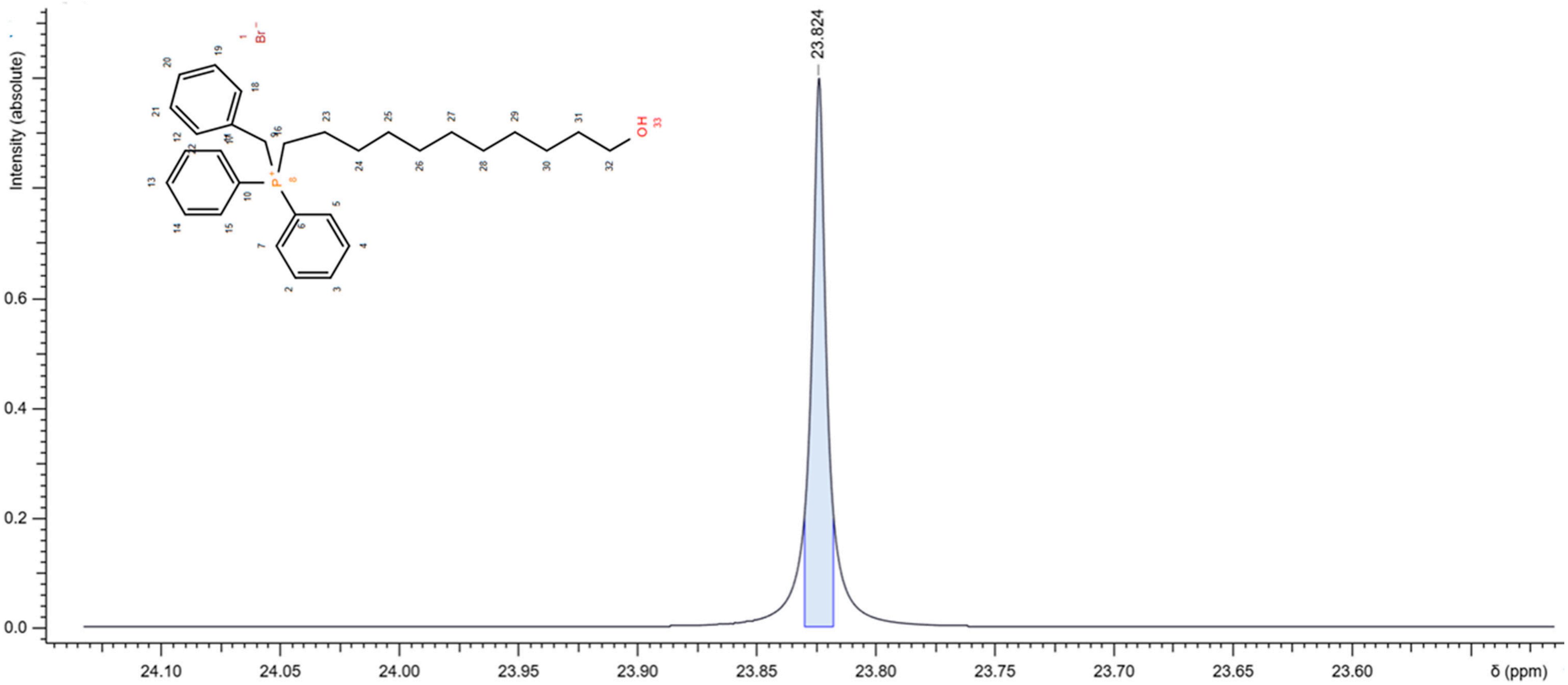

Figure S8. ^{31}P NMR spectrum (161 MHz, $CHCl_3$) of compound **2**.

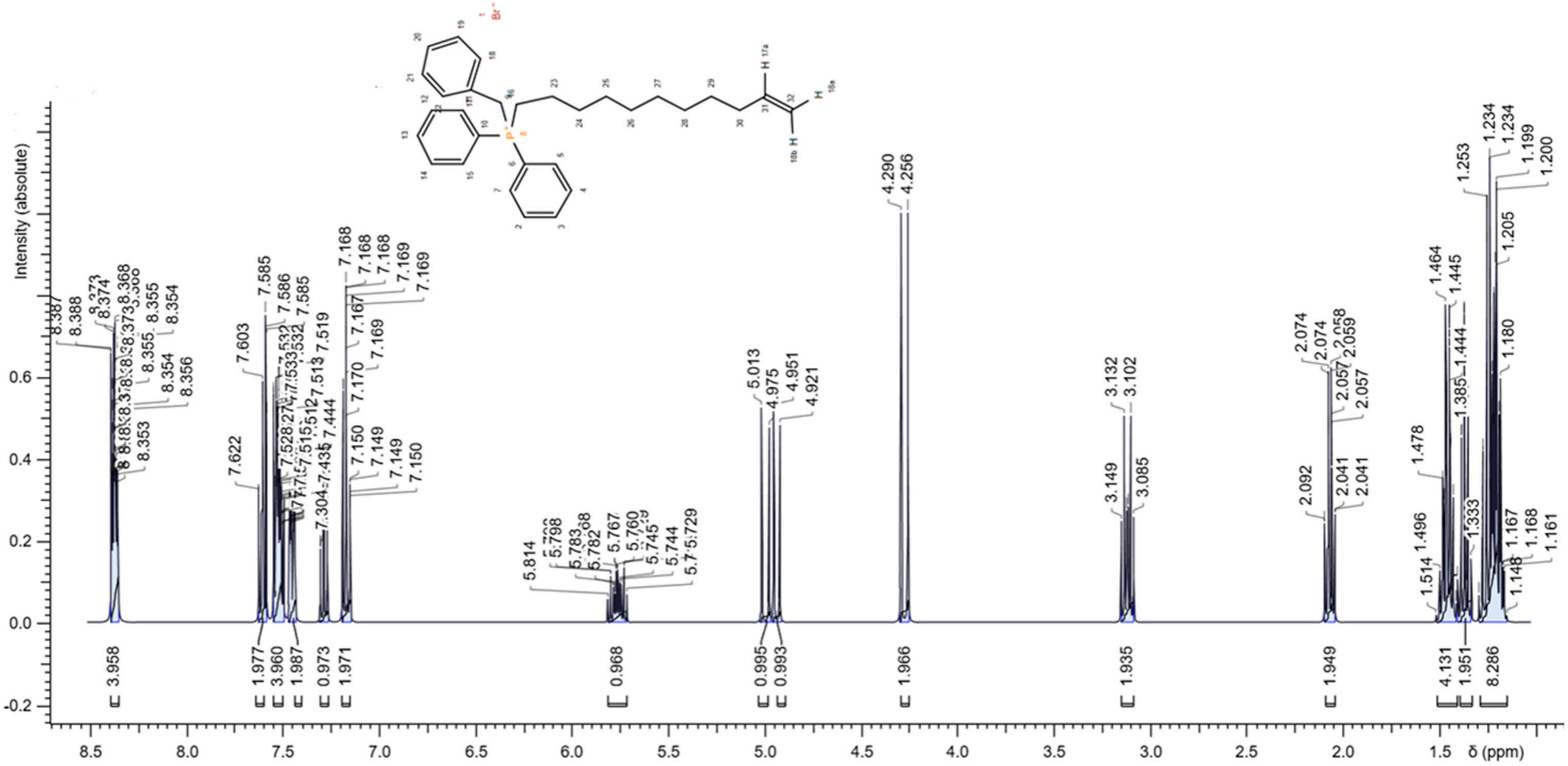

Figure S9. ^{1}H NMR spectrum (400 MHz, $CHCl_3$) of compound **3**.

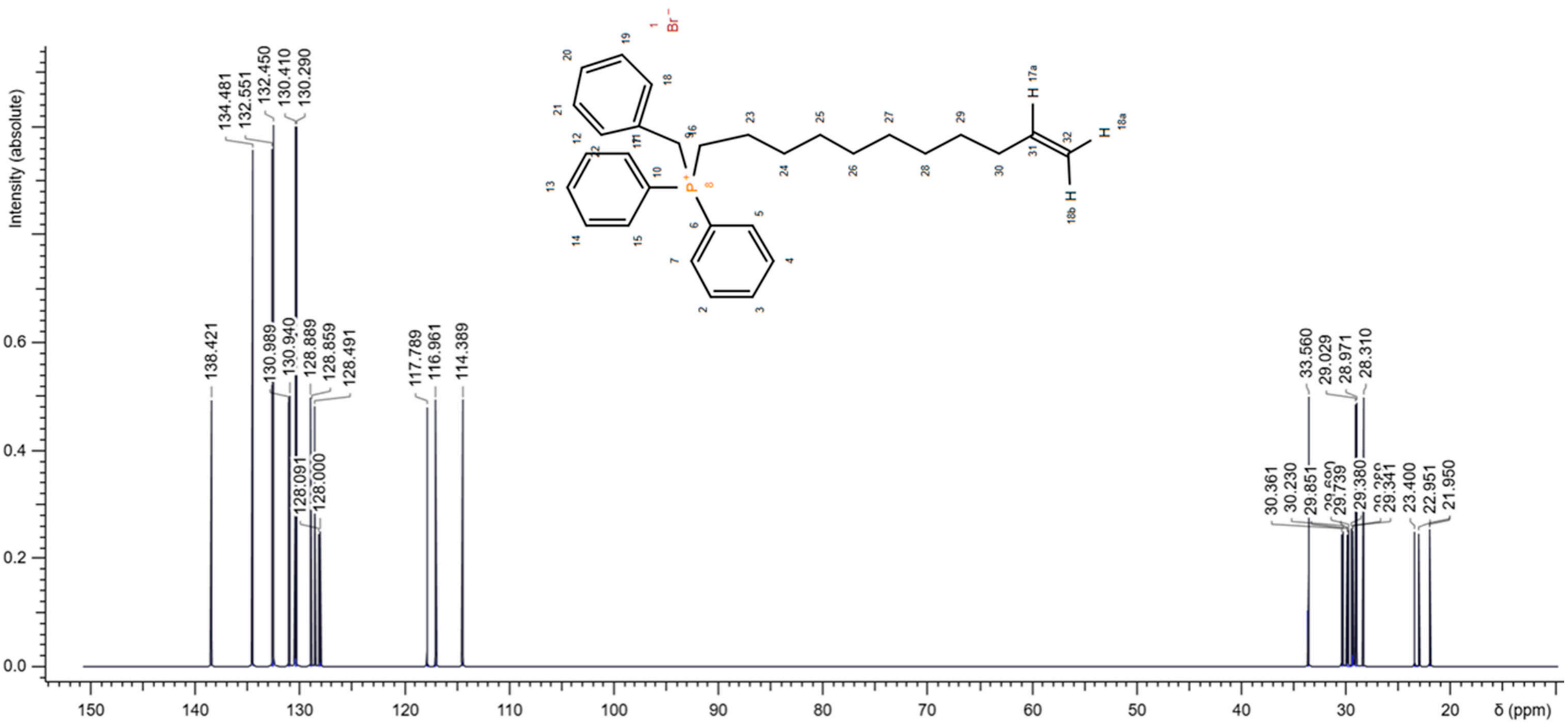

Figure S10. ¹³C NMR spectrum (100 MHz, $CHCl_3$) of compound **3**.

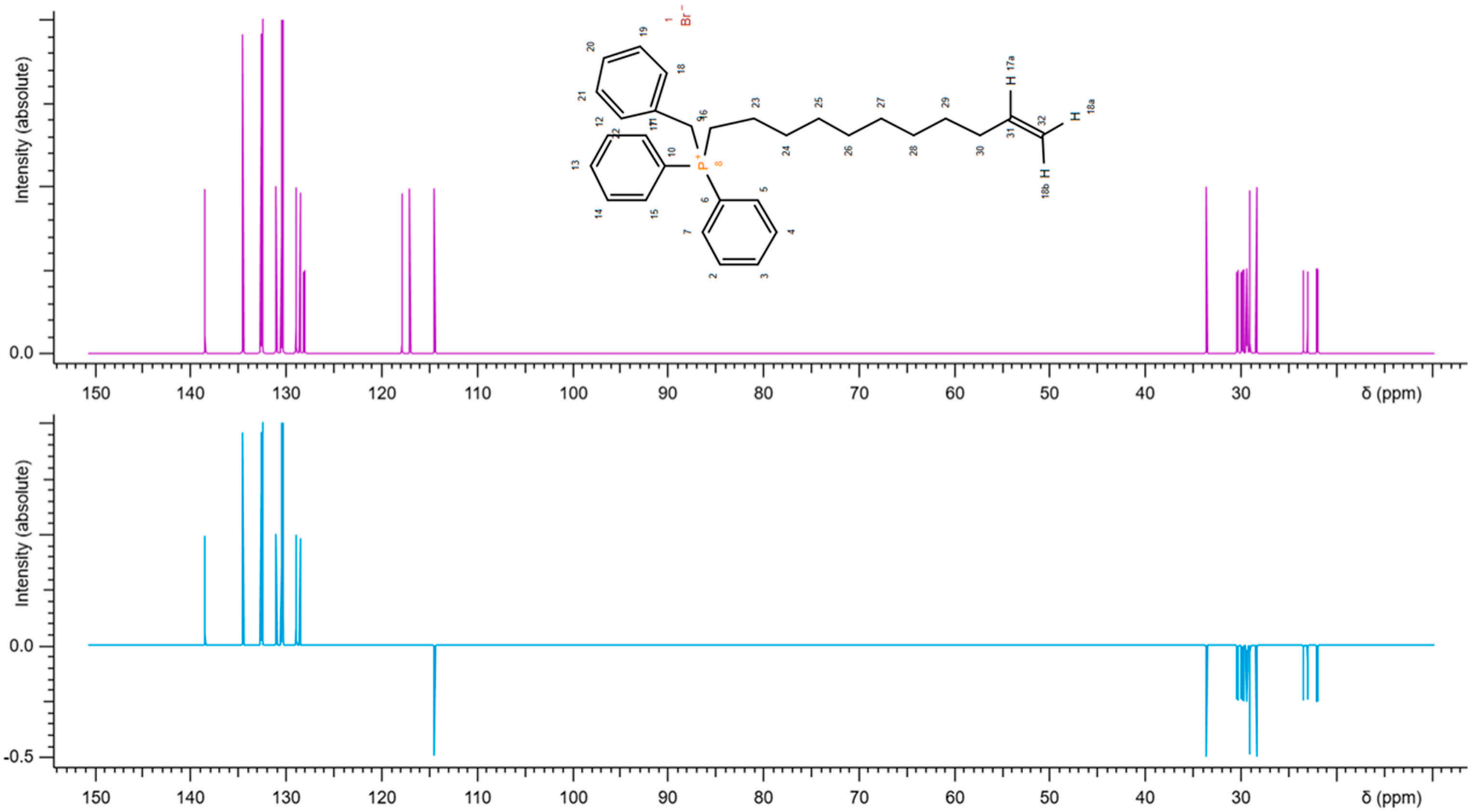

Figure S11. ^{13}C NMR spectrum (100 MHz, $CHCl_3$) and DEPT135 experiment of compound **3**.

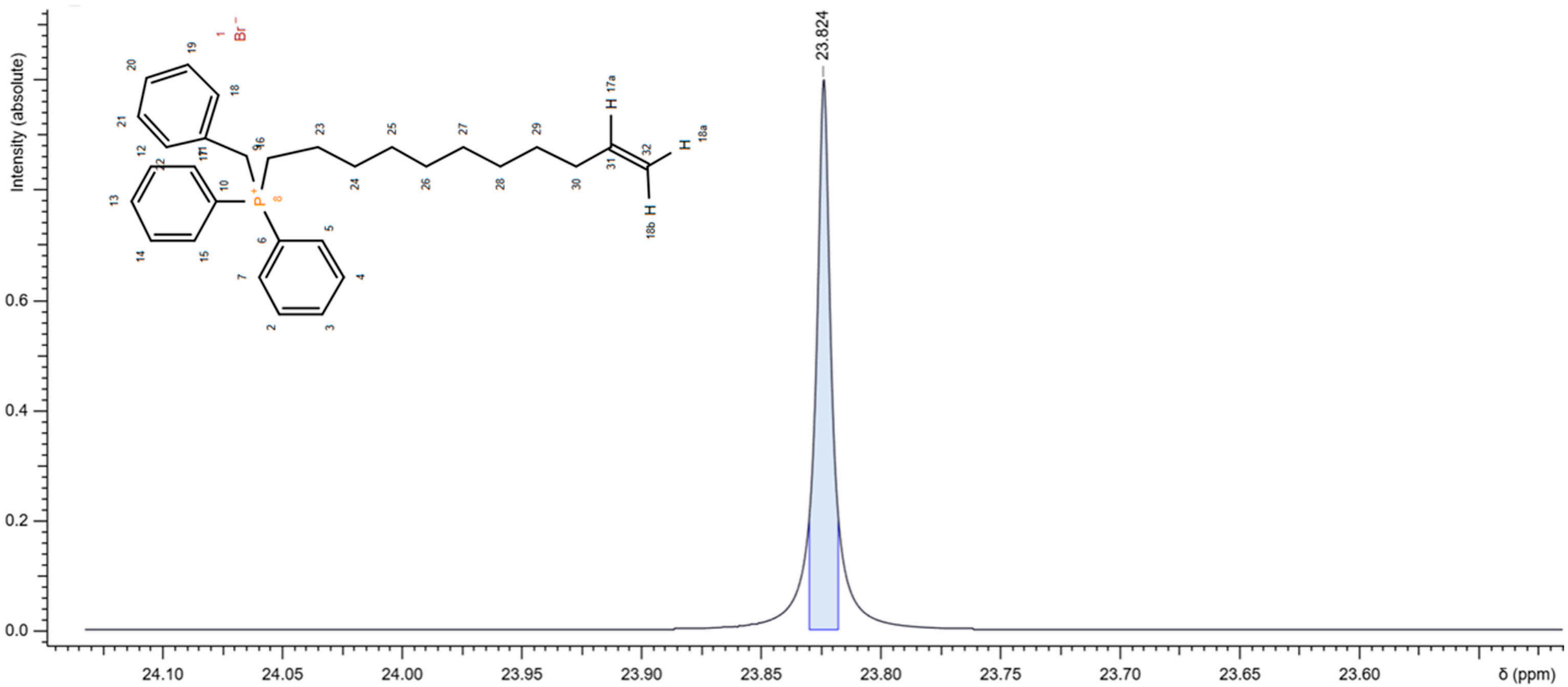

Figure S12. ^{31}P NMR spectrum (161 MHz, $CHCl_3$) of compound **3**.

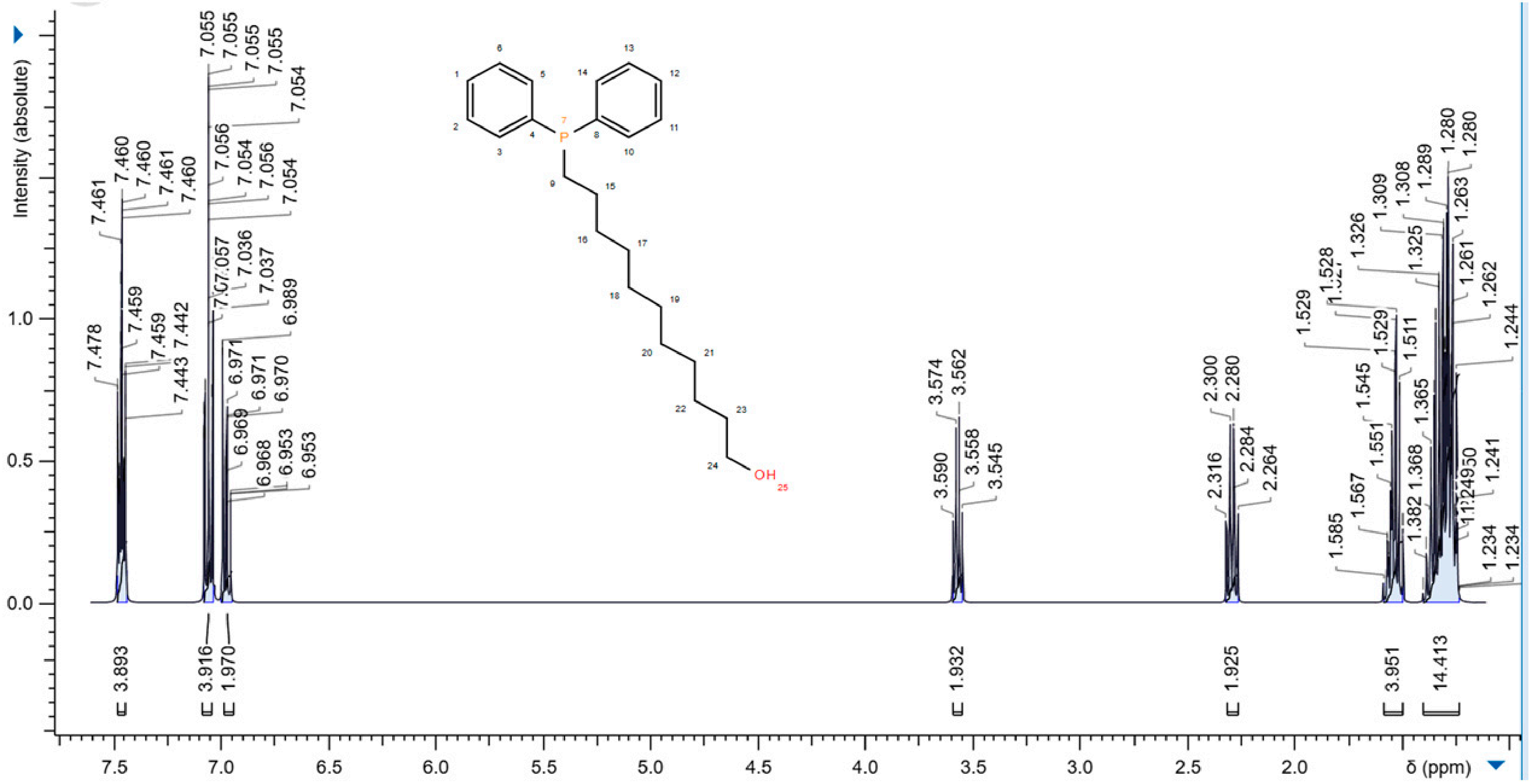

Figure S13. ^{1}H NMR spectrum (400 MHz, $CHCl_3$) of compound **4**.

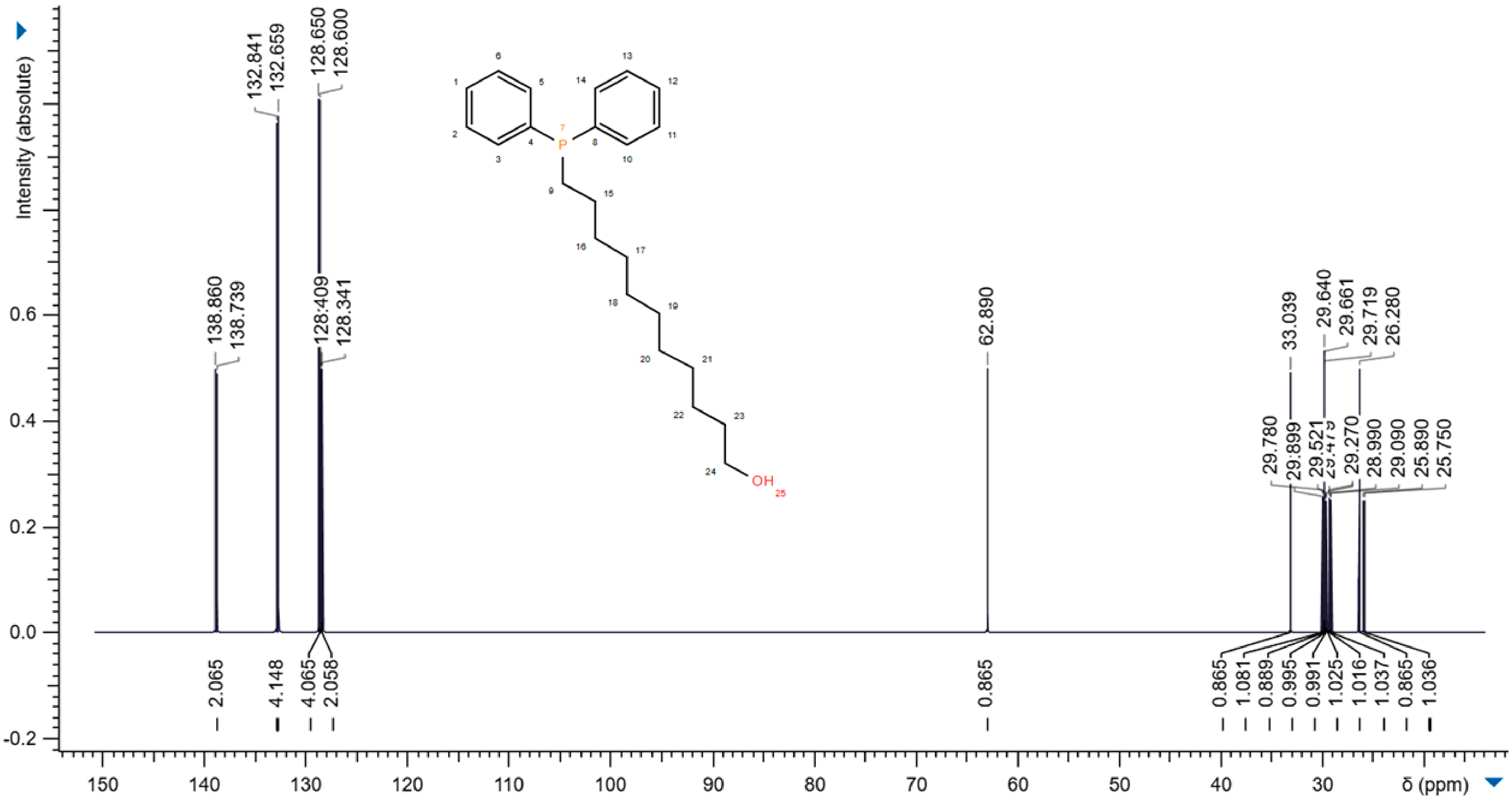

Figure S14. ^{13}C NMR spectrum (100 MHz, $CHCl_3$) of compound **4**.

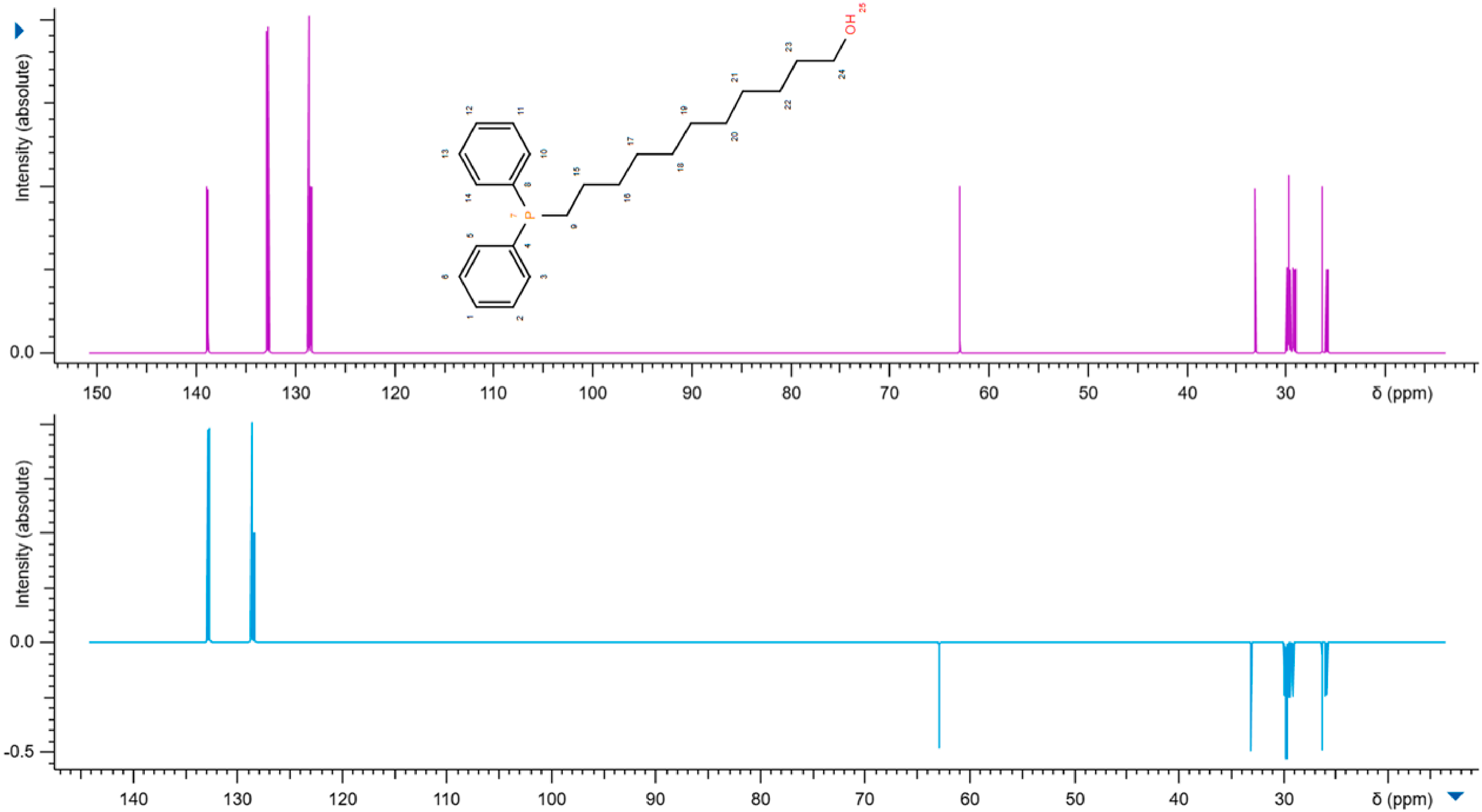

Figure S15. ^{13}C NMR spectrum (100 MHz, $CHCl_3$) and DEPT135 experiment of compound **4**.

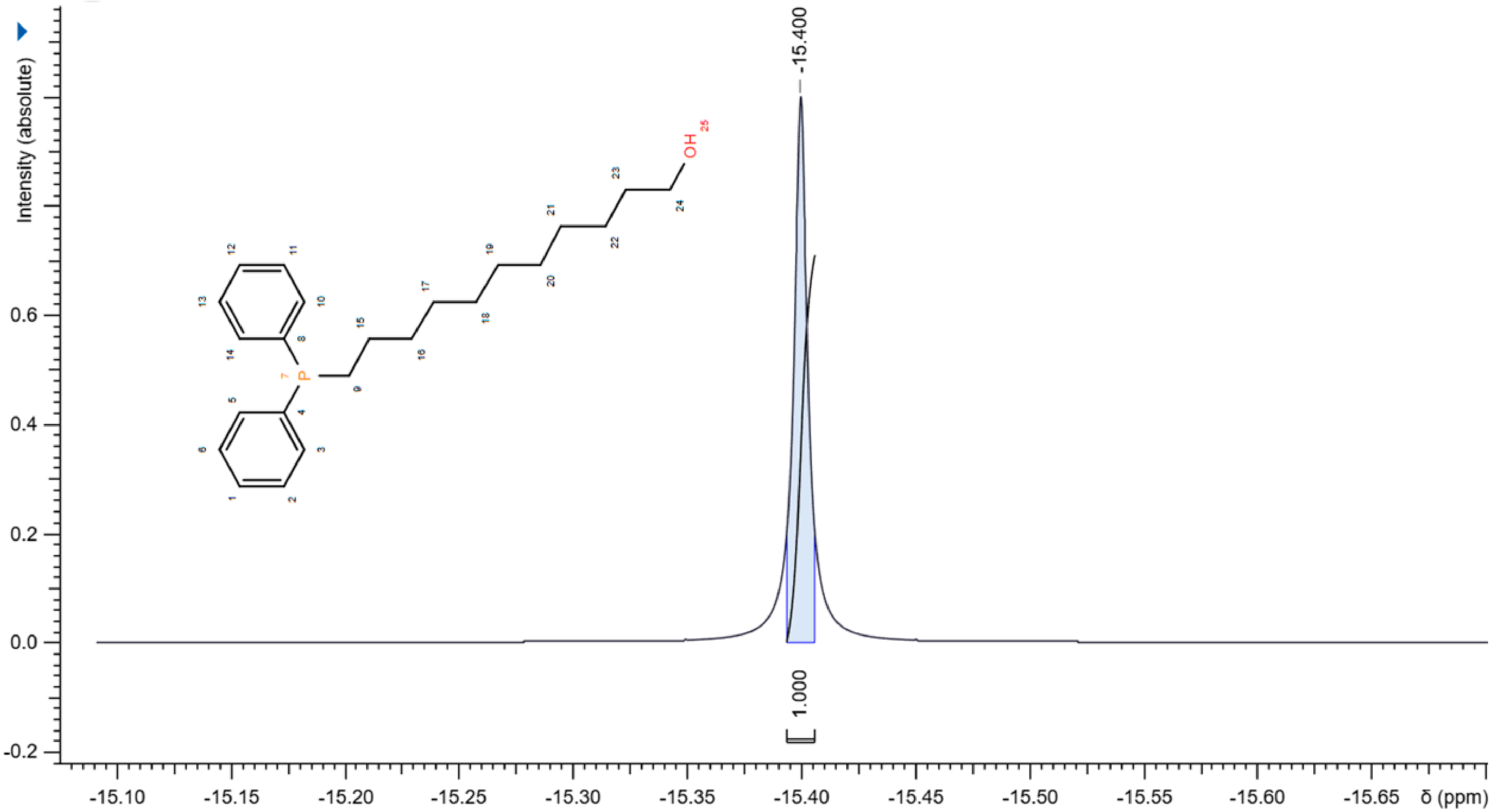

Figure S16. ^{31}P NMR spectrum (161 MHz, $CHCl_3$) of compound **4**.

Section S3. ATR-FTIR of Compounds **1-4**.

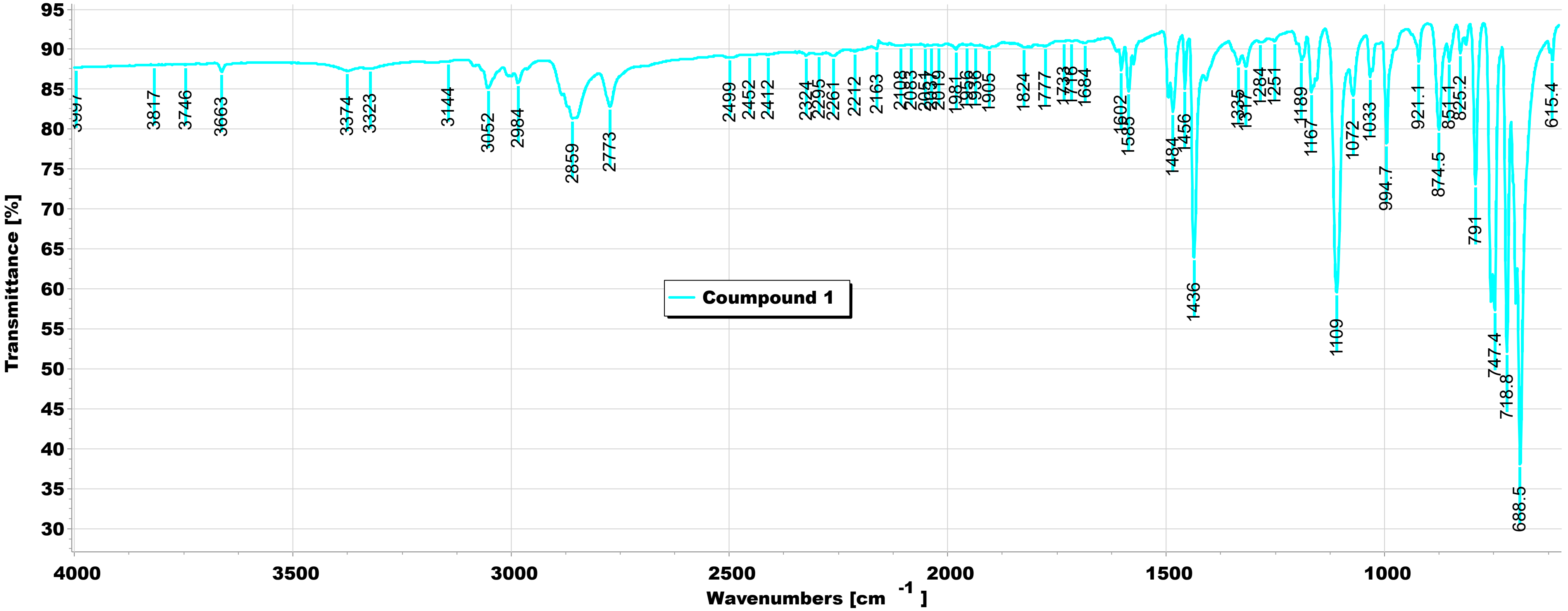

Figure S17. ATR-FTIR spectrum of **1**.

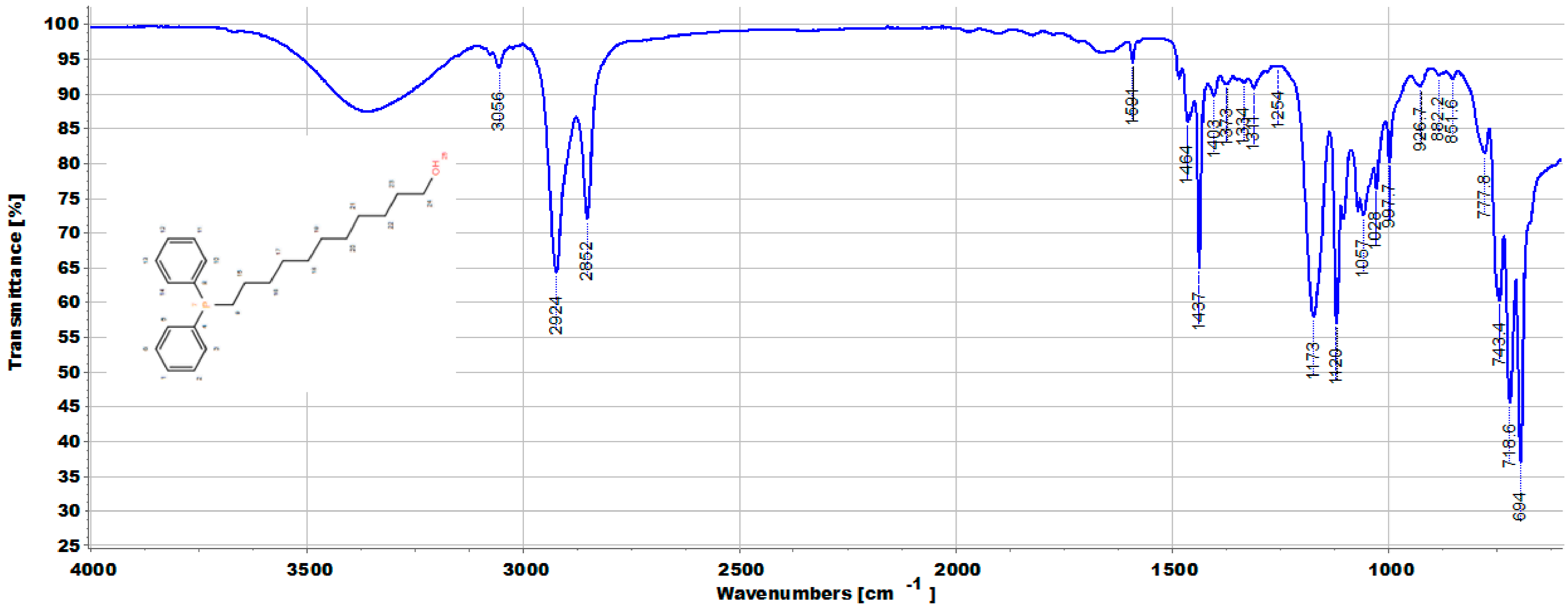

Figure S18. ATR-FTIR spectrum of **2**.

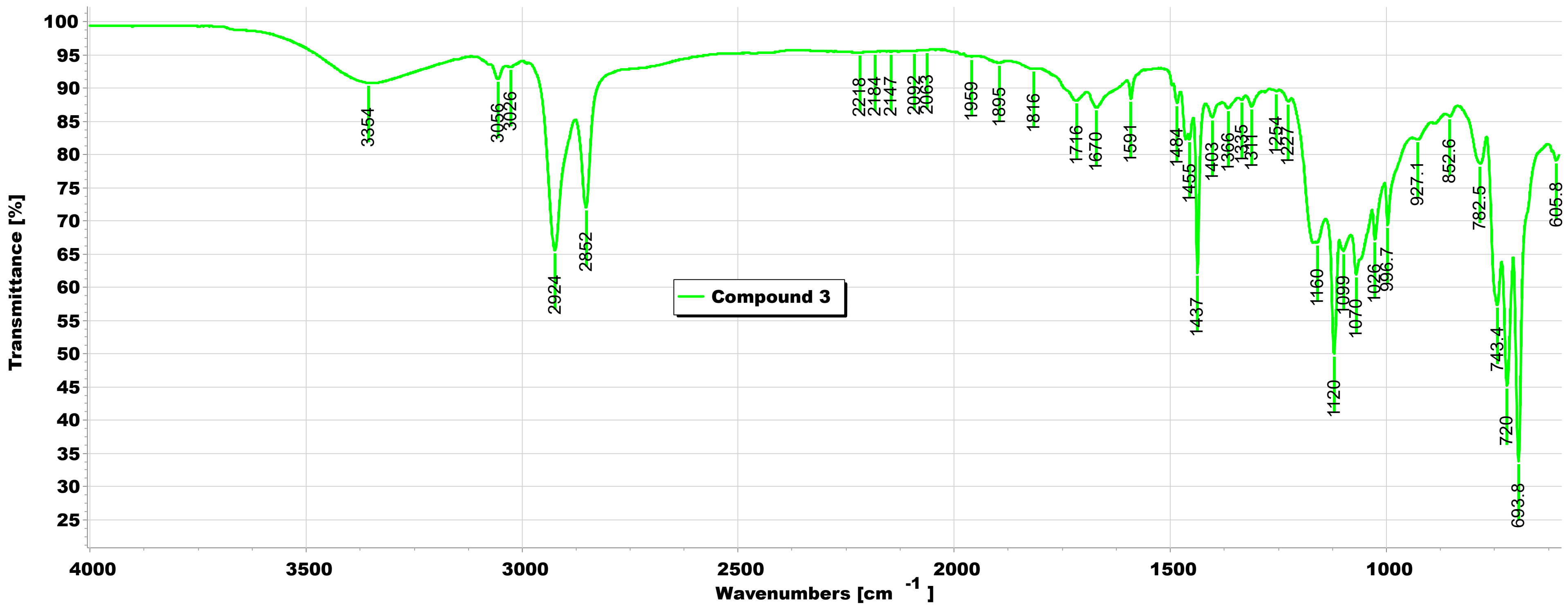

Figure S19. ATR-FTIR spectrum of **3**.

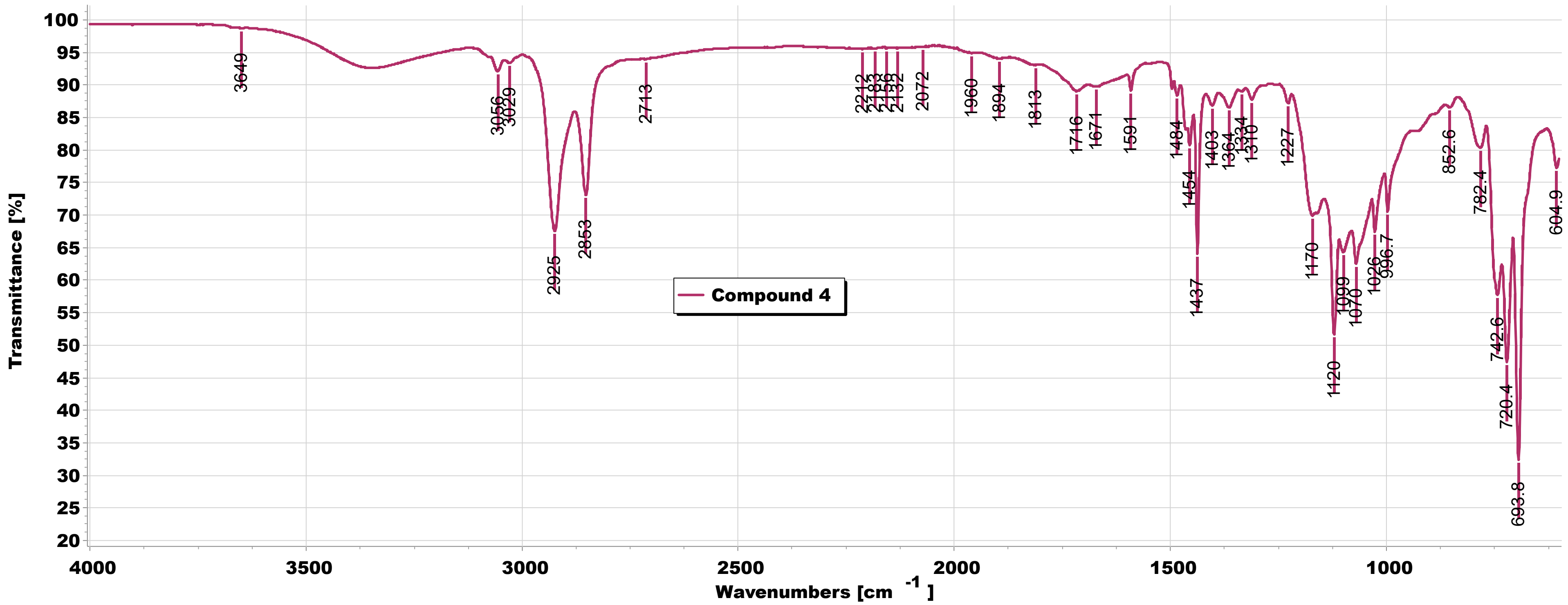

Figure S20. ATR-FTIR spectrum of 4.

Section S4. UV-Vis Spectra of Compounds **1-4**

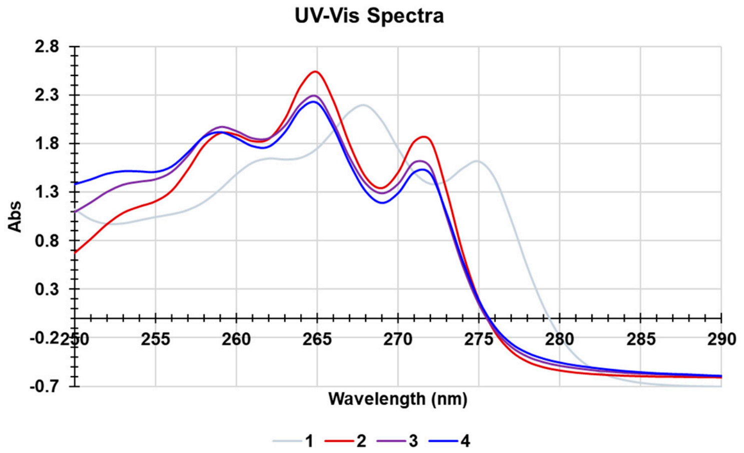

(a)

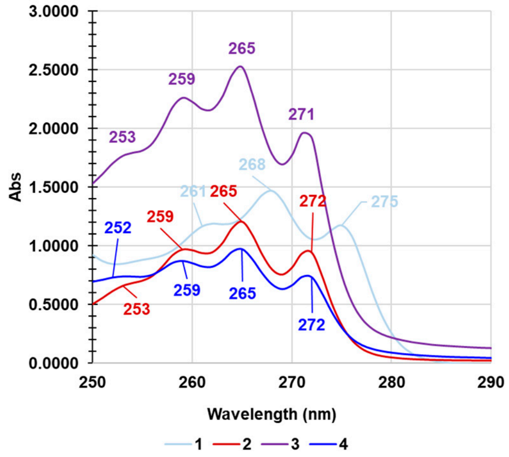

(b)

Figure S21. UV-Vis spectra of compounds **1-4** achieved reporting in graph the standardized csv data provided by the spectrometer using Microsoft 365 Excel software (a) and the not standardized one with peak labels added (b).

Section S5. In Solution Optic Microscopy Analyses on Compounds **1-4**

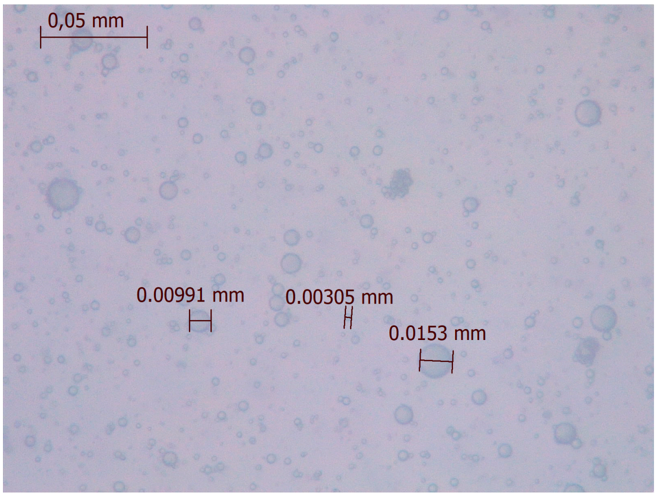

(A)

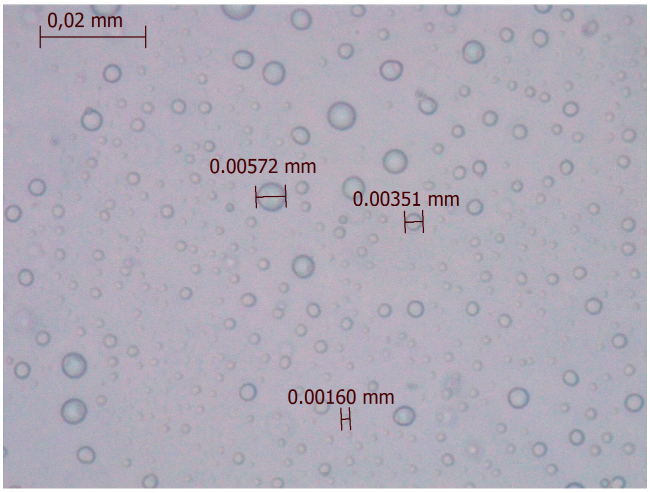

(B)

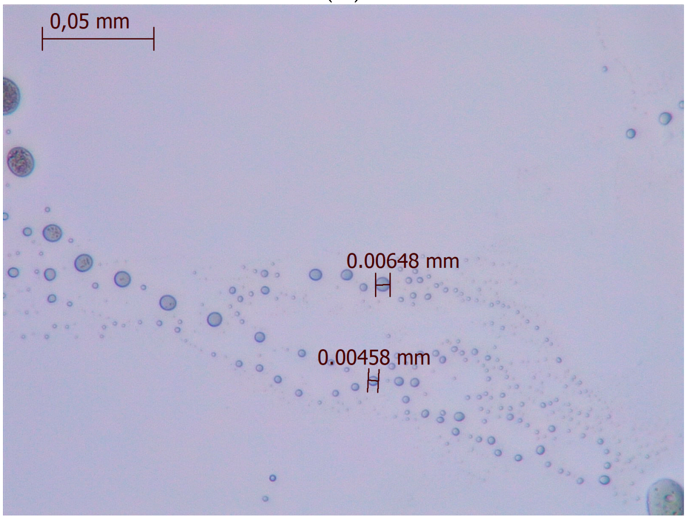

(C)

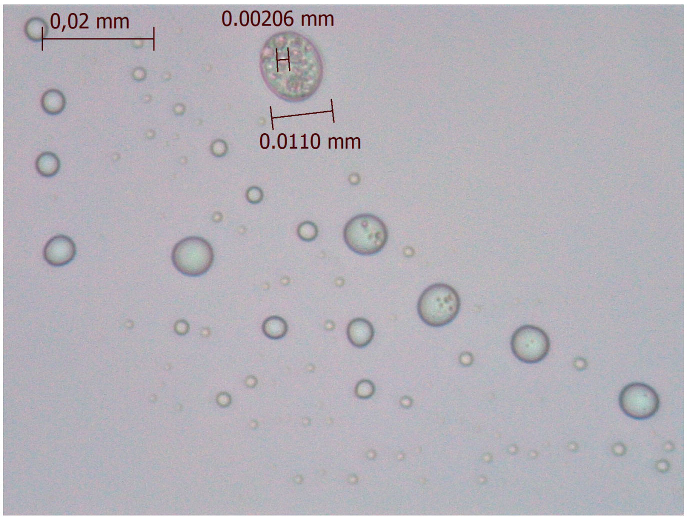

(D)

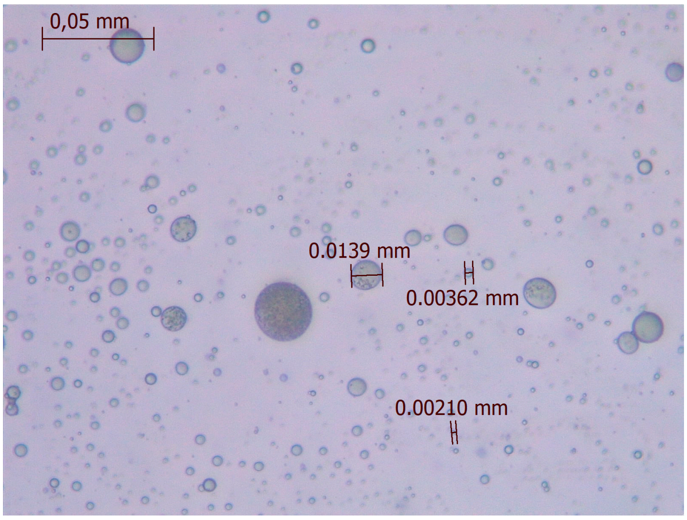

(E)

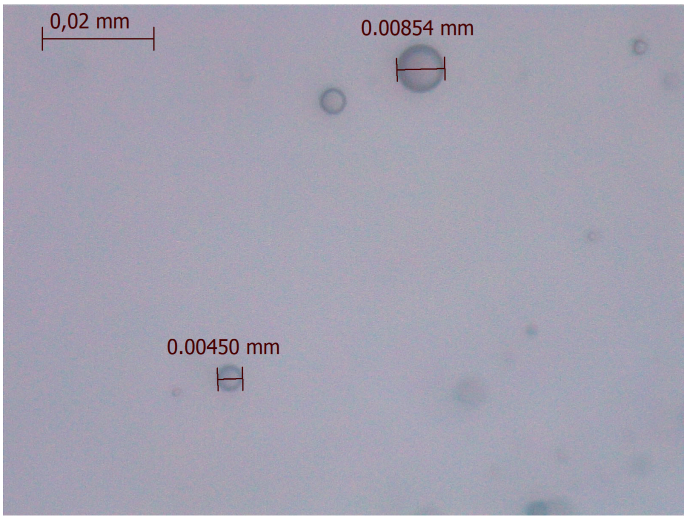

(F)

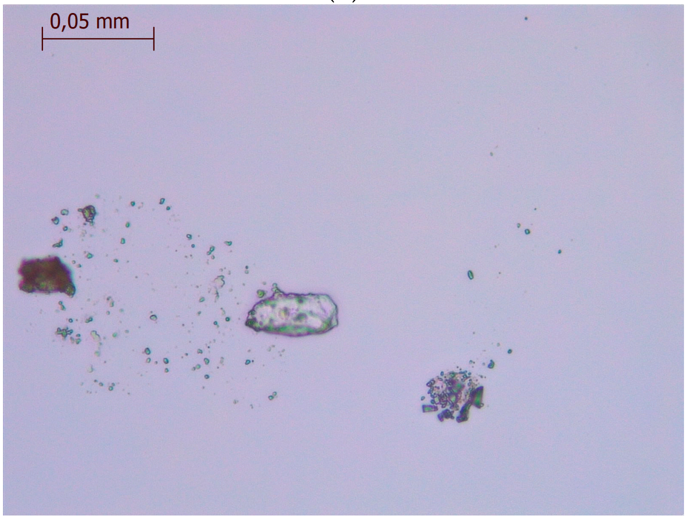

(G)

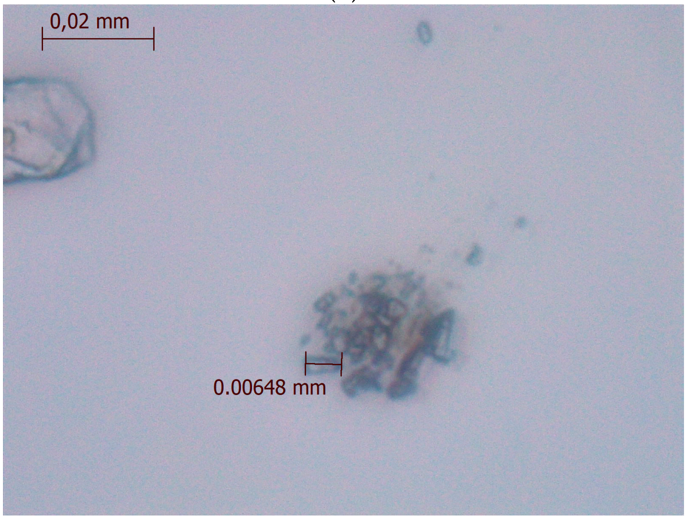

(H)

Figure S22A–H**.** Vesicular aggregates of **2** (A), **3** (C) and **4** (E), as well as aggregates of **1** (G) in water solution observed with a 40× objective. Smaller spherical vesicles of same compound were better evidenced using the 100× objective (B, D, F, H).

Section S6. DLS Analyses

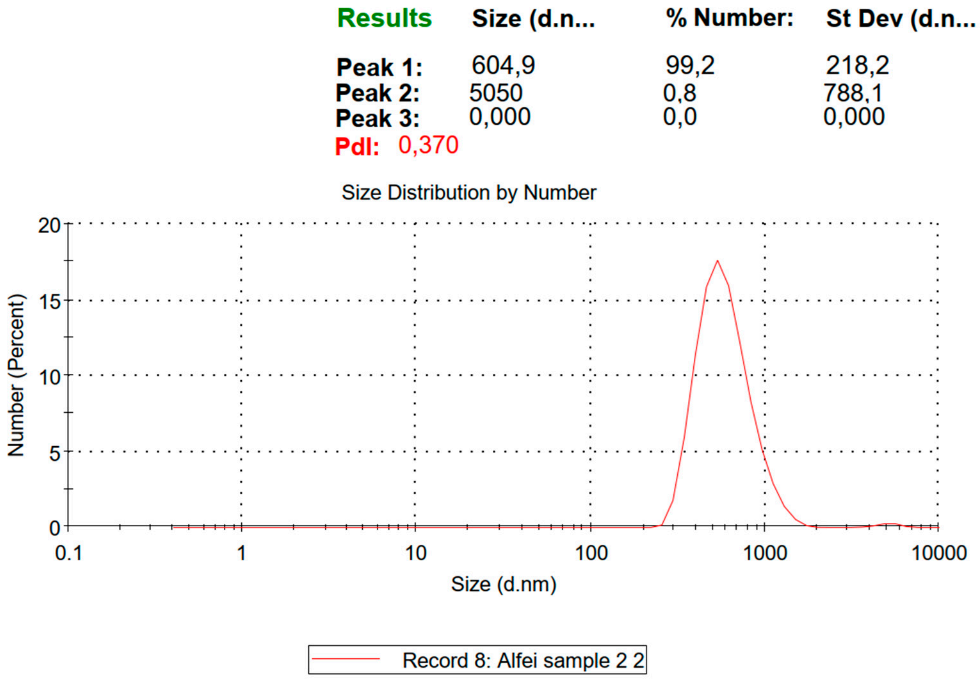

(A)

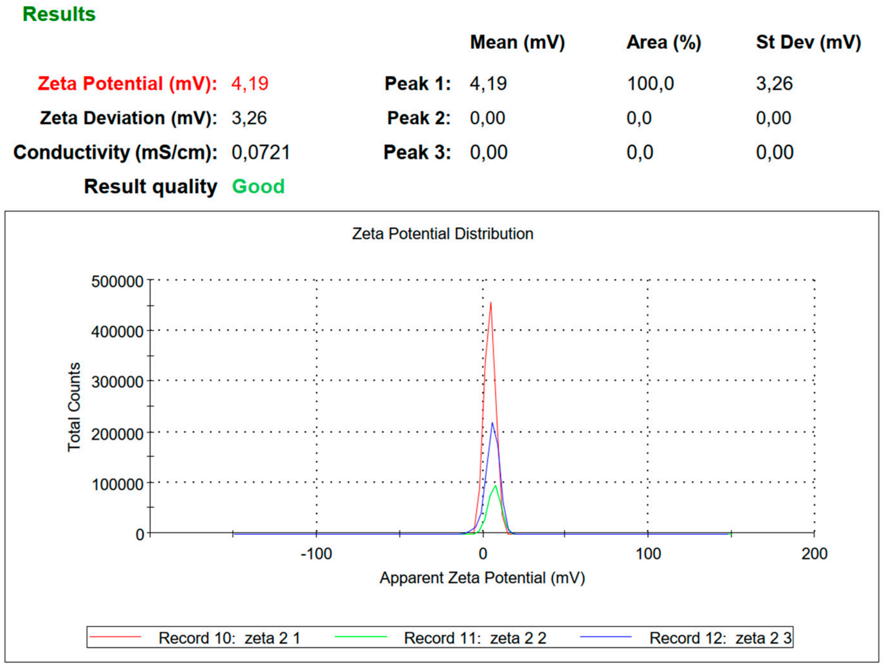

(B)

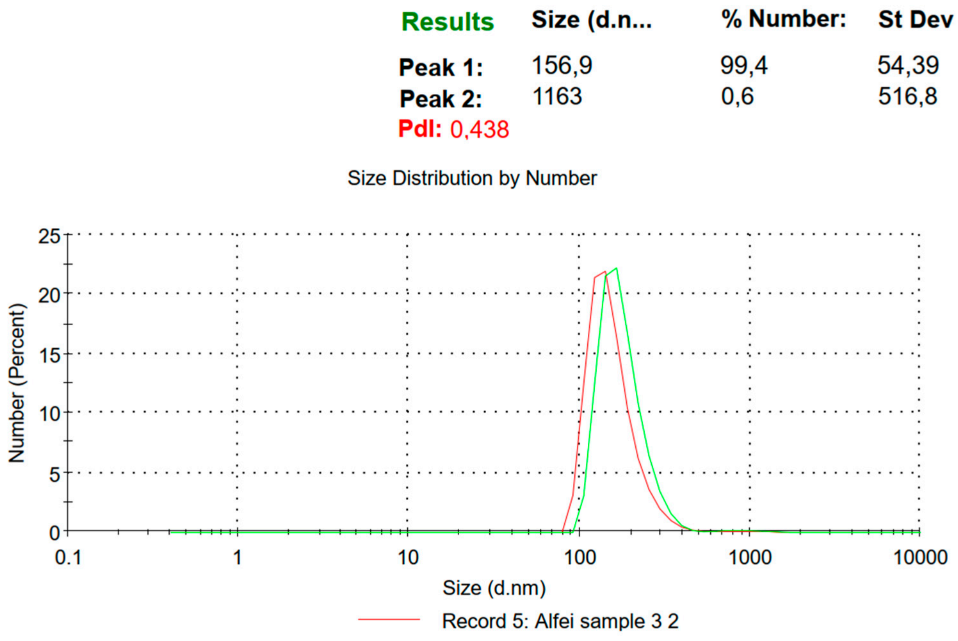
Results
Size (d.n...
% Number:
St Dev (d.n...
Peak 1: 156,9 99,4 54,39
Peak 2: 1163 0,6 516,8
PdI: 0,438
Size Distribution by Number
Number (Percent)
Size (d.nm)
Record 5: Alfei sample 3 2

(C)

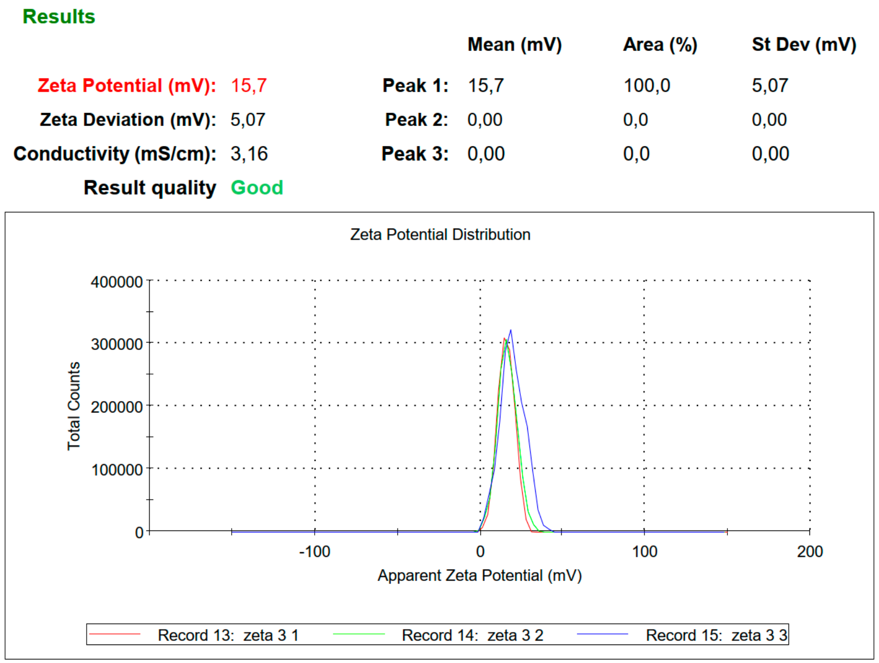
Results
Mean (mV)
Area (%)
St Dev (mV)
Zeta Potential (mV): 15,7
Peak 1: 15,7 100,0 5,07
Zeta Deviation (mV): 5,07
Peak 2: 0,00 0,0 0,00
Conductivity (mS/cm): 3,16
Peak 3: 0,00 0,0 0,00
Result quality Good
Zeta Potential Distribution
Total Counts
Apparent Zeta Potential (mV)
Record 13: zeta 3 1
Record 14: zeta 3 2
Record 15: zeta 3 3

(D)

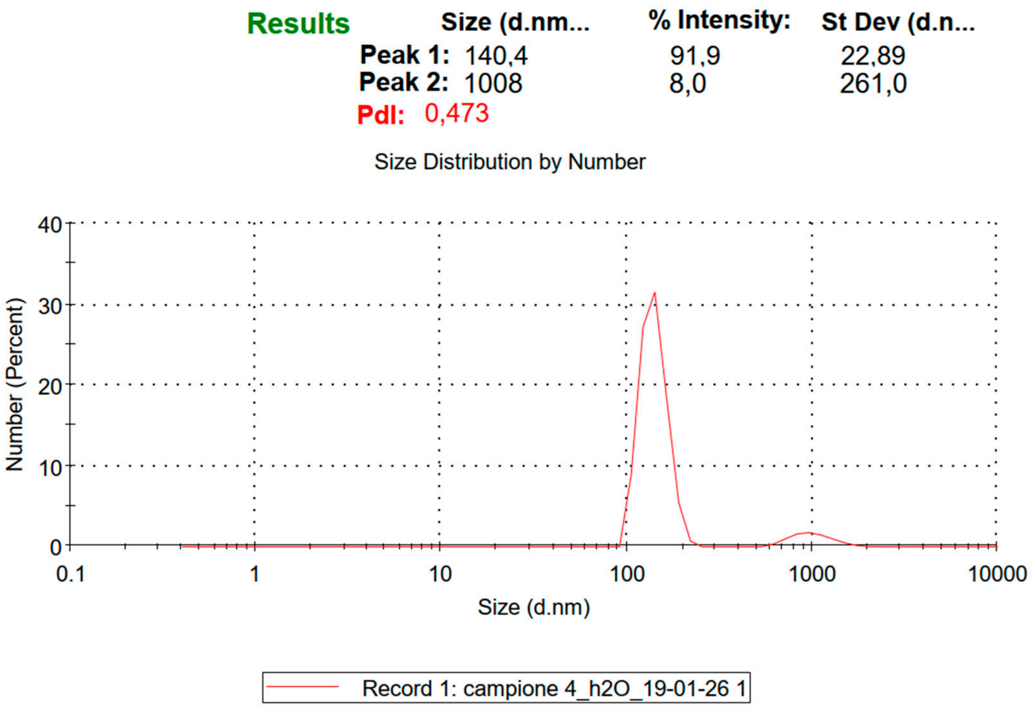

(E)

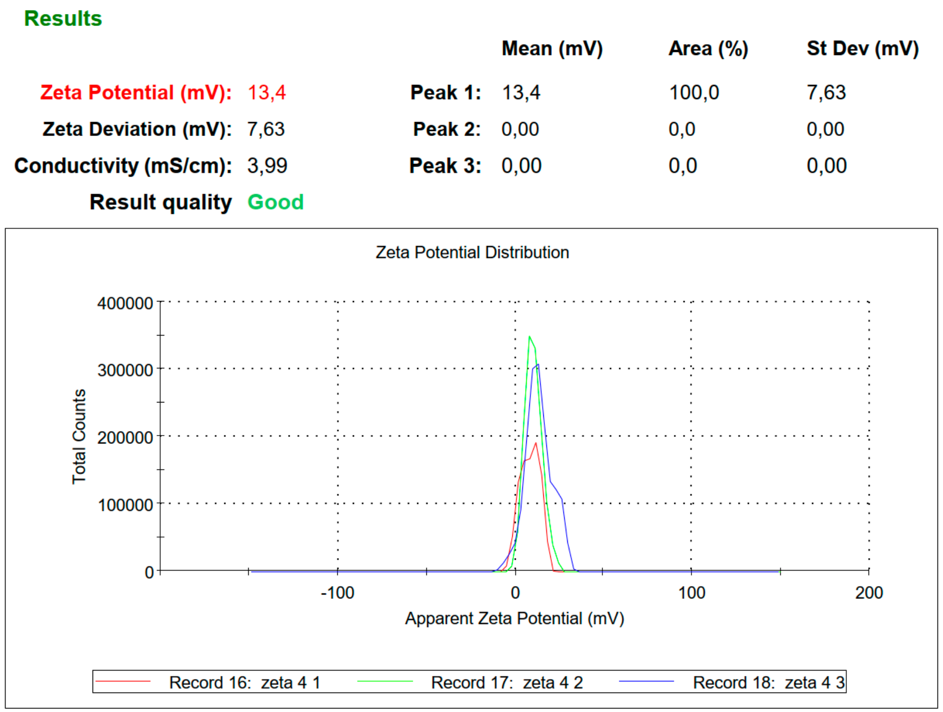

(F)

Figure S23A–F. (A, C, D) Average hydrodynamic diameter (Z-AVE, nm) distributions, obtained by records acquired by number (%) at the highest kcps. (B, D, F) Average zeta-potential (ζ-p, mV) distributions obtained by the three records.

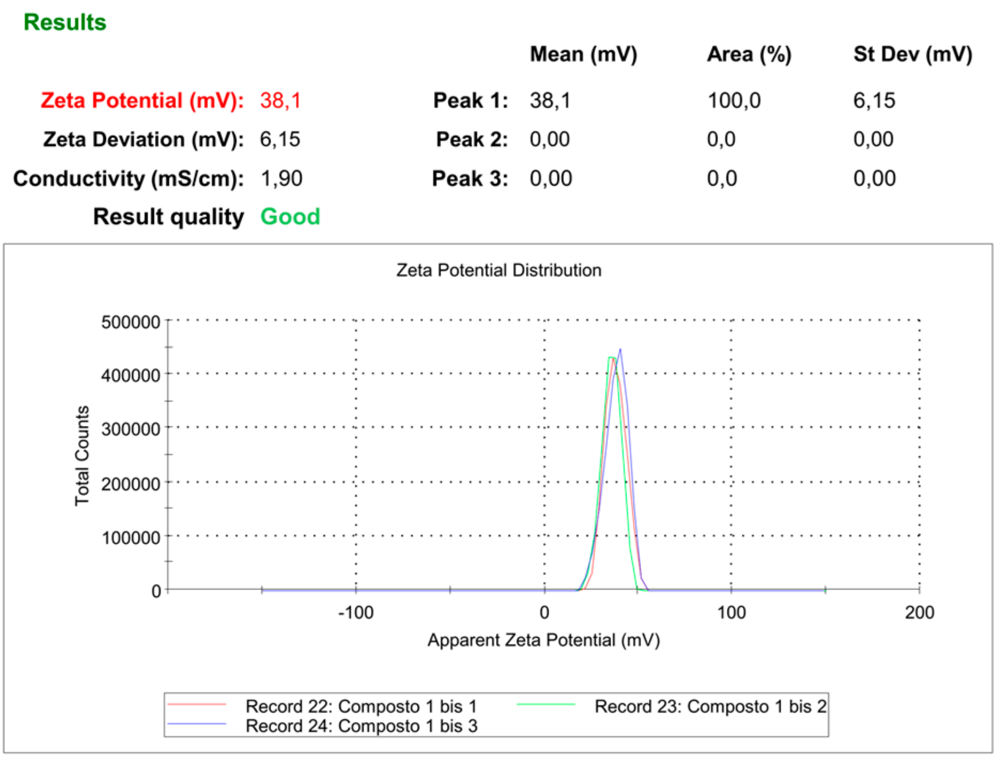

Figure S24. Three records of the Zeta-potential (ζ-p, mV) distributions of **1** (143.3 kcps), acquired at 25°C in water.

Section S7. Antibacterial Effects of Compounds **1-4**

Table S7.1. MICs of compounds **1-4** against MDR clinical isolates of a narrow selection of Gram-positive and Gram-negative species obtained from experiments conducted at least in triplicate. The modal value was considered as MIC, and results have been reported expressed as μg/mL.

**Strains**	**1** (433.3)[1]	**2** (356.5)[1]	**3** (527.5)[1]	**4** (509.5)[1]	**V/T**	**OXA**
		**MIC μg/mL**				
S. aureus ATCC 29213 ^{MSSA}	16	N.L.	N.L.	N.L.	0.5$^{(V)}$	0.5
S. aureus B ^{MRSA}	8	N.L.	N.L.	N.L.	0.25$^{(V)}$	512
S. epidermidis 22 ^{MRSE}	16	N.L.	N.L.	N.L.	0.5$^{(V)}$	128
E. faecalis 1$^{\text{VRE, *}}$	64	N.L.	N.L.	N.L.	256$^{(V)}$; 64$^{(T)}$	N.R.
E. faecium 152 $^{\text{VRE, *}}$	64	N.L.	N.L.	N.L.	128$^{(V)}$; 64$^{(T)}$	N.R.
P. aeruginosa 259 $^{\text{**, CF, CR}}$	> 128	N.L.	N.L.	N.L.	N.R.	128
E. coli $^{\text{**, ***}}$	> 128	N.L.	N.L.	N.L.	N.R.	128
K. pneumoniae $^{\text{**, ***}}$	> 128	N.L.	N.L.	N.L.	N.R.	256

[1] MW of compounds; MSSA = methicillin-sensitive; VRE = vancomycin-resistant enterococci; MRSA = methicillin resistant *S. aureus*; MRSE = methicillin resistant *S. epidermidis*; * denotes resistance also to teicoplanin; ** indicates resistance to carbapenems; CF= from patients with cystic fibrosis; CR = resistant to colistin; *** indicates resistance to carbapenems by producing class A *K. pneumoniae* carbapenemase ; N.L. = not legible; V = vancomycin; T = teicoplanin; OXA = oxacillin; N.R. = not reported.

Section S8. Structural Consideration for Compounds **1-4**

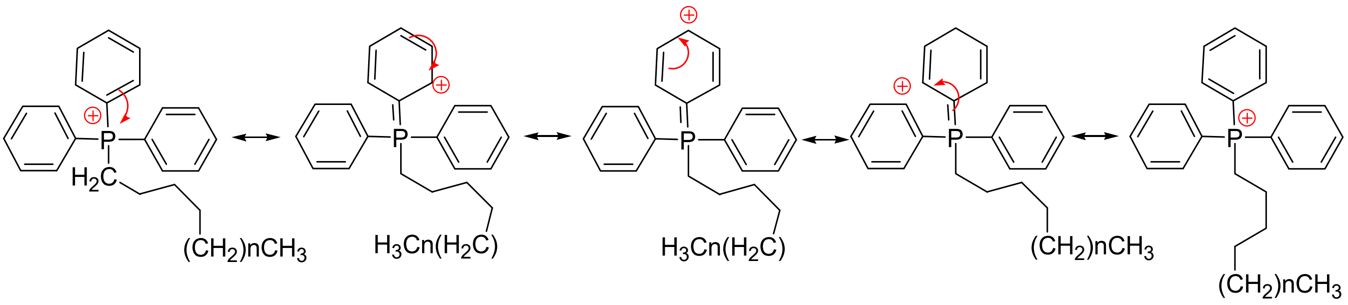

4 (for each ring) x 3 (number of rings) possible limit resonance formula leading to 12 possible cationic structures with enlarged cationic radii

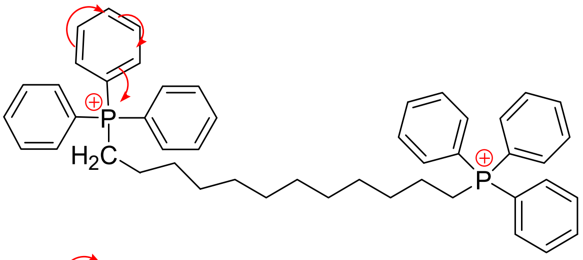

For both cationic heads, 4 x 3 possible limit resonance formula leding to 12 possible cationic structures for both cationic heads, with enlarged cationic radii

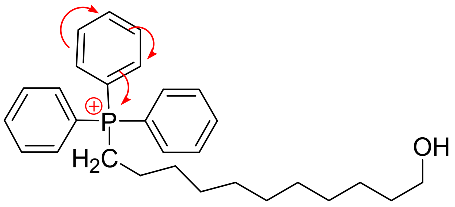

4 x 3 possible limit resonance formula leding to 12 possible cationic stuctures with enlarged cationic radii

(A)

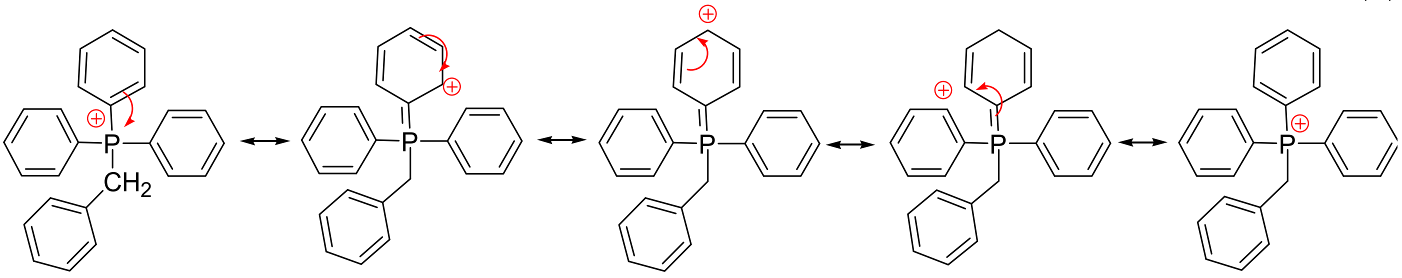

4 (for each ring) x 3 (number of rings) possible limit resonance formula leading to 12 possible cationic structures with enlarged cationic radii

(B)

4 x 2 possible limit resonance formula providing 8 possible cationic cationic structures with enlarges cationic radii

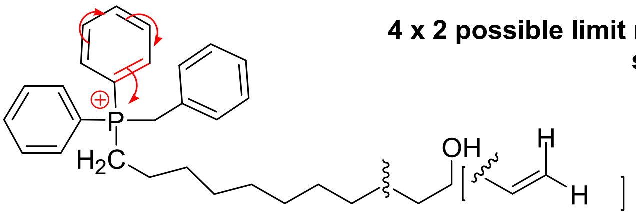

(C)

Scheme S2A–C. Possible inductive and resonance delocalization and stabilization of cationic charge on phosphorous atom in phenyl phosphonium salts.

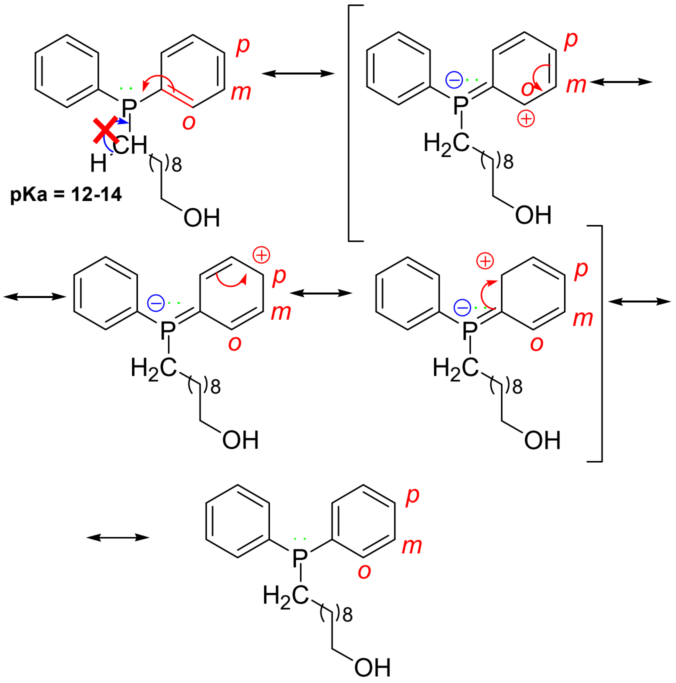

Scheme S3. Possible π-resonance for compound **2**, and its three-limit resonance formula possible for each phenyl ring, having large cationic radii.

Section S9. Kinetics Study for Time-Kill Curves

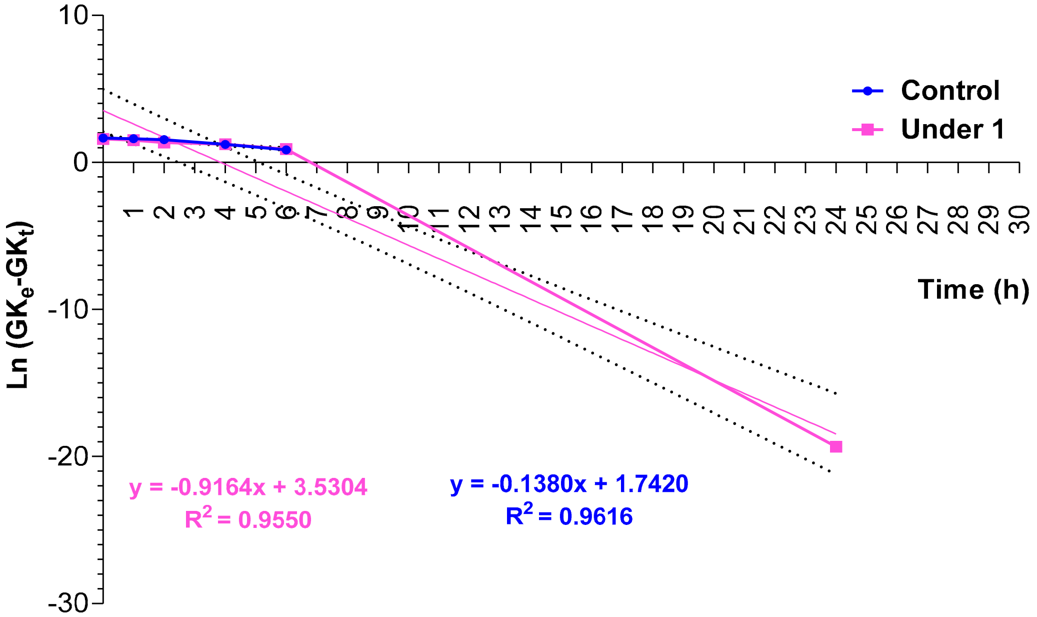

(A)

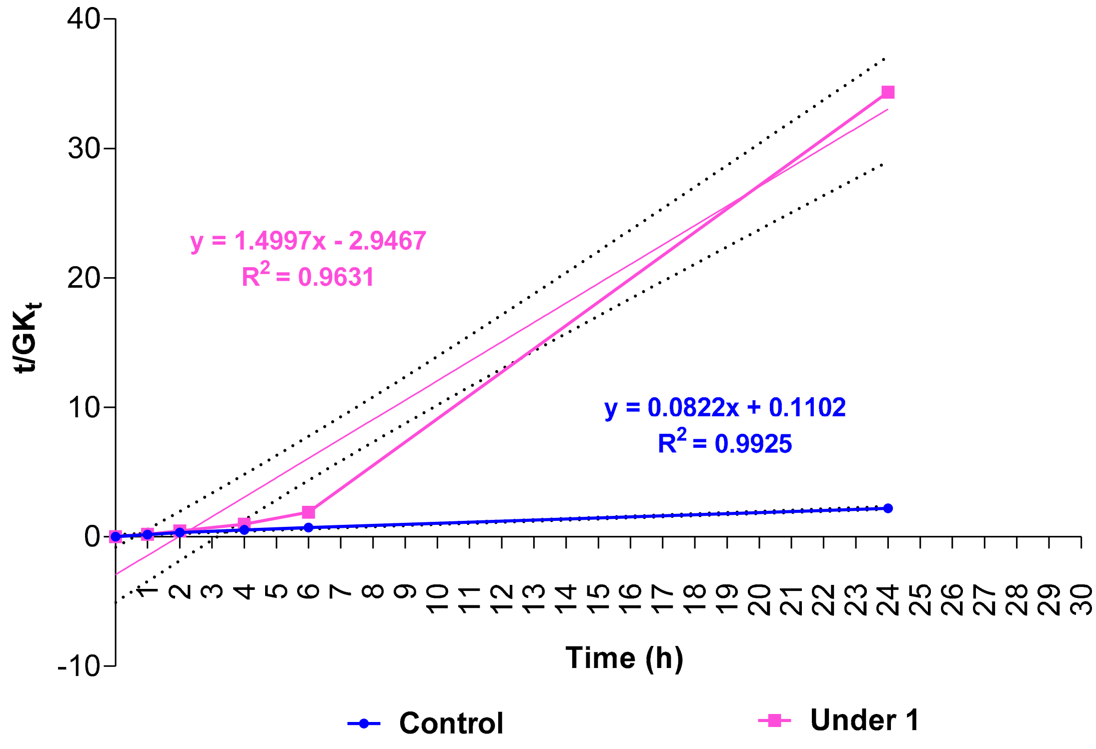

(B)

Figure S25. PFO (A) and PSO (B) models fitting the time-kill data (blue lines with round indicators for control and pink lines with square indicators for bacteria under treatment with **1**) with related linear regression lines (thinner lines). Punctuated curves indicate the confidence intervals (95%) around the regression lines.

Table S1. Values of coefficients of determinations (R^2) obtained for PFO and PSO kinetic models considered.

Kinetic Model	Control	1
PFO	0.9616	0.9550
PSO	**0.9925**	**0.9631**

Table S2. Comparison of experimental number of bacterial colonies (in the control and under treatment with **1**) at the end of time-kill experiment with *GKe* form the kinetic model and values of K_{PSO} provided by it.

Parameter	CTR *	CTR (EXP)	1	1 (EXP)
GKe (Log_{10}CFU/mL)	11.34 (*Ge*)	10.91	0.6668 (*Ke*)	0.6990
K_{PSO} **	0.0706	N.A.	0.5089	N.A.

GKe = Log_{10} (CFU/mL) at the end of time-kill process (*Ge* means bacteria grown in the control, while *Ke* means bacteria killed when treated with **1**); CTR = control; EXP = experimental; * Computed from the slopes of the equations in Figure 5; ** computed from the intercepts of equations in Figure 5.

Table S3. Comparison of K_{PSO} values of **1** with those reported for other TPP-salts and QASs, as well as those of P7 and P5.

Compounds	Mathematical Model	K_{PSO}	Time (h)	Refs.
TPP- salts	PSO	0.5681	24	[47,82,109]
QASs		714.9	8	[110,111]

P7 (QAPs)	1.25	24	[72]
P5 (QAPs)	3.07	24	[73]
**1**	0.5089	24	This work

TPP = triphenyl phosphonium; QASs = quaternary ammonium salts; QAPs = quaternary ammonium polymers.

Section S10. Biofilm Inhibition Experiments with **1** and Vancomycin

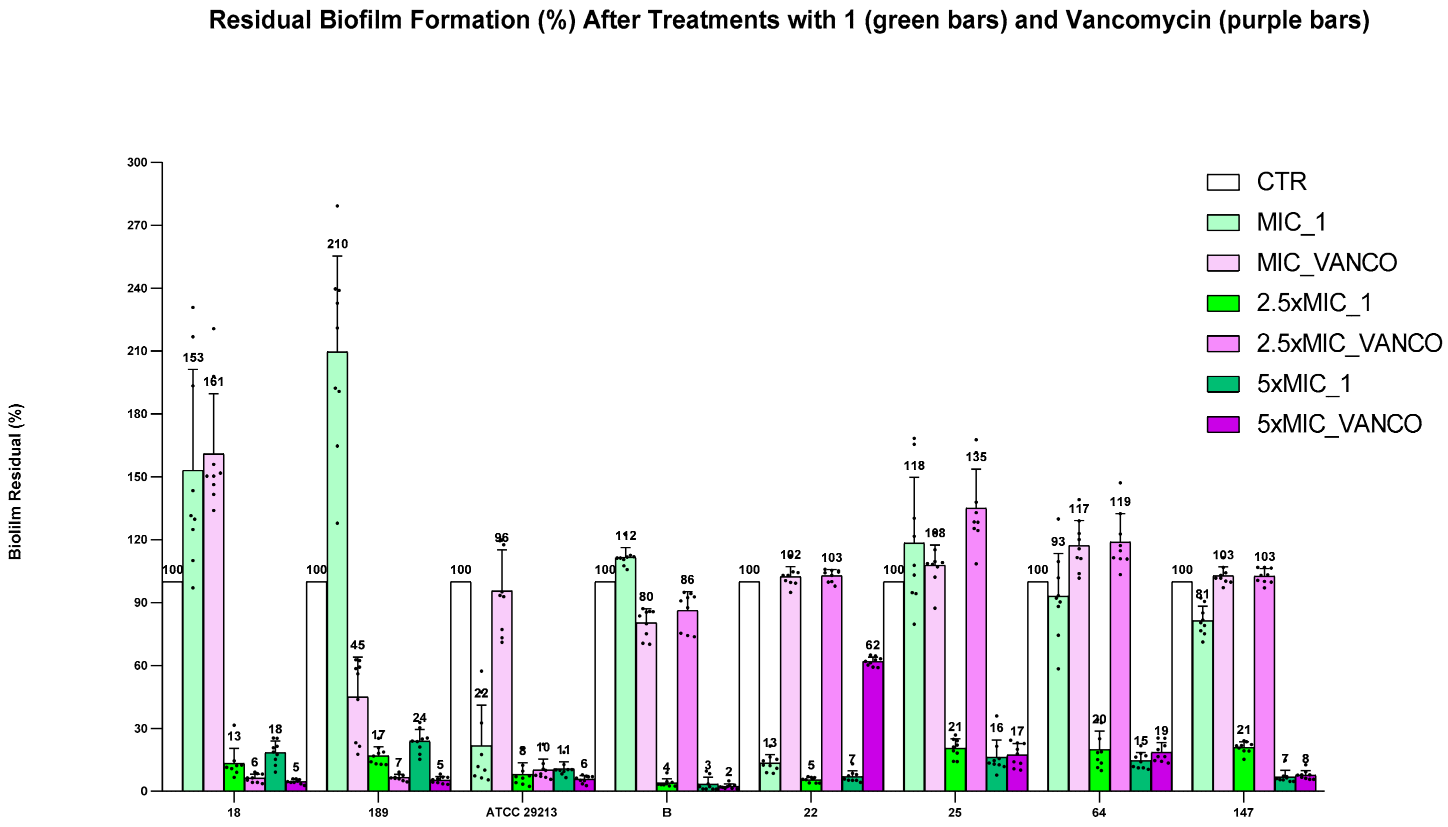

Figure S26. Residual biofilm formation respect to controls (withe bars, 100%) after treatment of *Staphylococcus aureus* (18, 189, ATCC 29213 and B) and *S. epidermidis* (22, 25, 64 and 147) species sensitive to vancomycin, with compound **1** (dispar numbers green bars) and vancomycin used as reference antibiotic (pair numbers purple bars) administered to bacteria at MIC, 2.5 × MIC and 5 × MIC (as in the legend). Statistical significance (not reported in graph) was obtained using GraphPad PRISM software 8.0.1 by the analysis of variance (Two-ways ANOVA) corrected for multiple comparisons using statistical Tukey hypothesis testing.

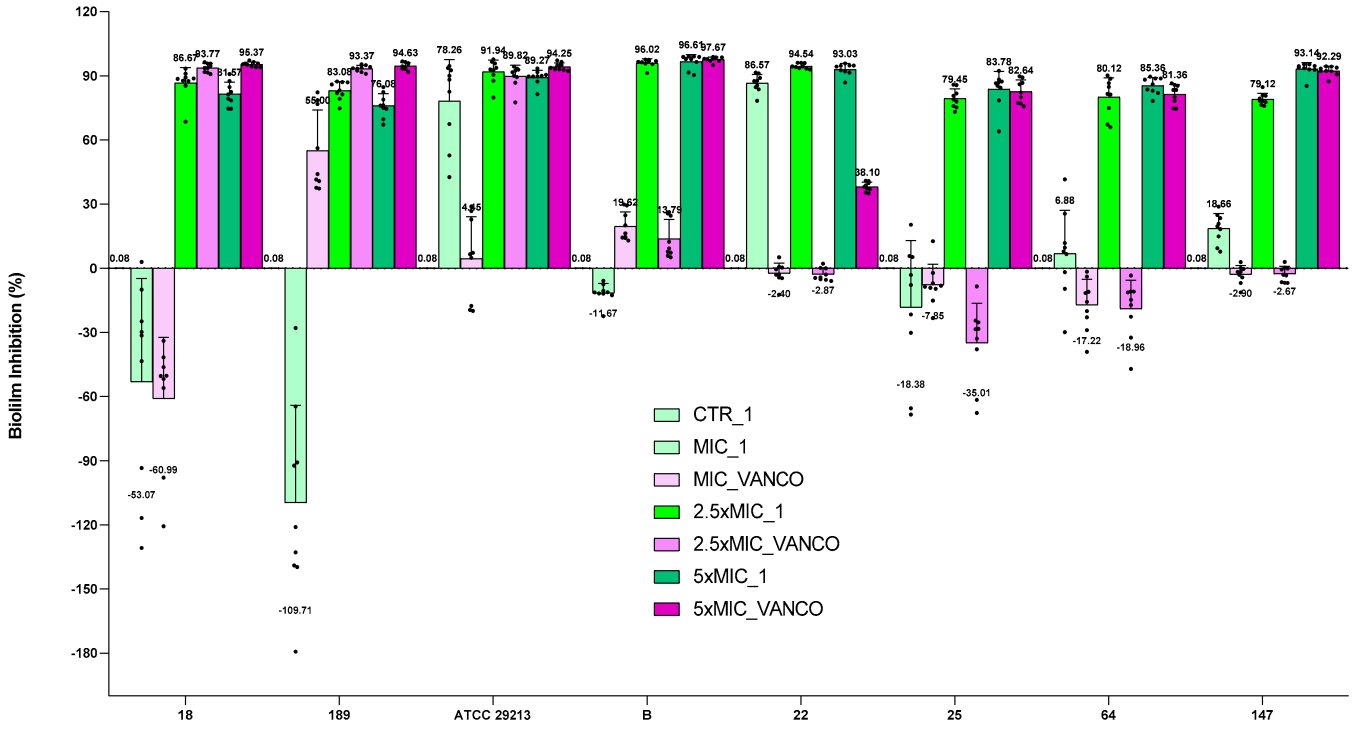

Figure S27. Biofilm inhibition percentage (%) of *Staphylococcus aureus* (18, 189, ATCC and B) and *S. epidermidis* (22, 25, 64 and 147) species sensitive to vancomycin, after no treatments (CTR, white bars, 0.08%) and after treatment with compound **1** (dispar numbers green bars) and vancomycin used as reference antibiotic (pair numbers purple bars) administered to bacteria at MIC, 2.5 × MIC and 5 × MIC (as in the legend). Statistical significance (not reported in graph) was obtained using GraphPad PRISM software 8.0.1 by the analysis of variance (Two-ways ANOVA) corrected for multiple comparisons using statistical Tukey hypothesis testing.

Table S4. Classification of BF production based on OD [123].

OD (Optical Density)	BF classification
< 0.1	Not adhering (No producer)
0.1 – 0.5	Weak BF producer
0.5 – 1.0	Moderate BF producer
> 1.0	Strong BF producer

Section S11. Cytotoxicity of Compound **1**.

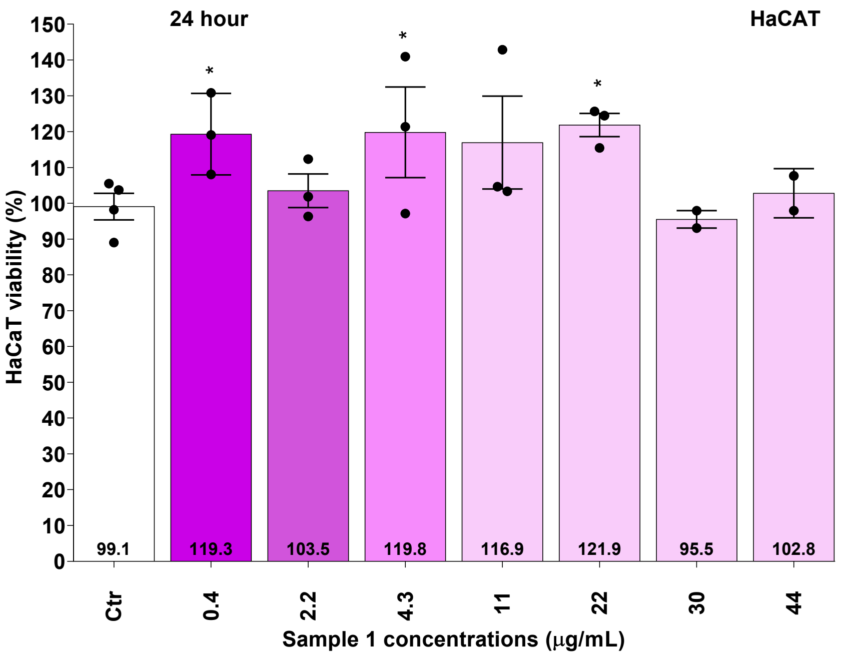

(A)

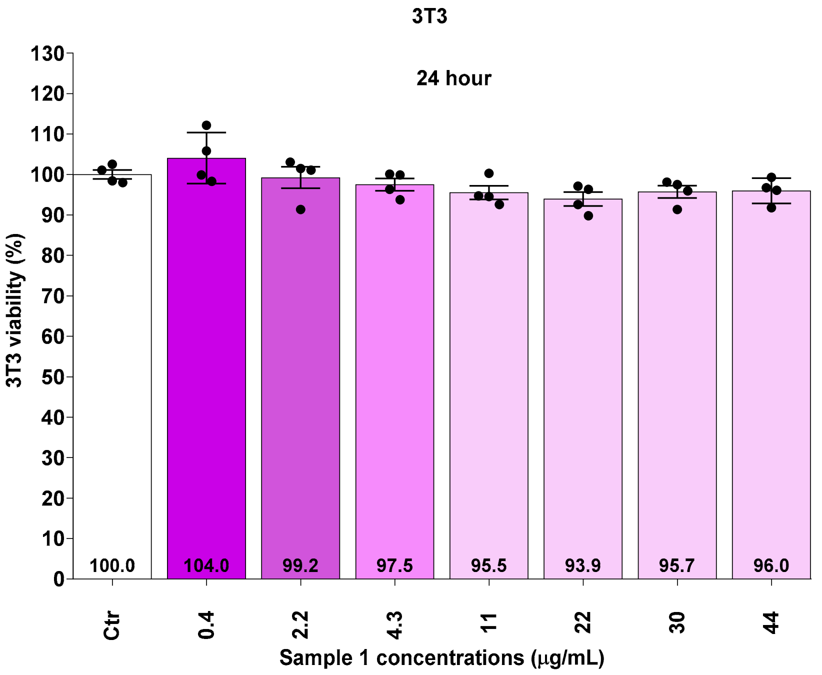

(B)

Figure S28. Cell viability was evaluated in HaCaT (A) and 3T3 (B) cells when exposed to increasing concentrations of **1** (0.4-44 μg/mL, 1–100 μM) for 24 hours. Bar graphs summarize quantitative data of the means ± S.D. of four independent experiments (black spheres) run in triplicate. Significance refers exclusively to control (*). Specifically, $p > 0.05$ no symbols; $p < 0.05$ * (one-way ANOVA followed by Dunnet's multi-comparisons test). Numbers at bottom within bars indicate viable cells (%).

Table S5. IC_{50} of **1** when tested on HaCaT and 3T3 cells in 24 hours treatments as for bacteria to determine MICs.

Compound (MW)	Cells	IC_{50} (µg/mL)
**1**	HaCaT	216.7±V.W. *
	3T3	656.9± V.W. *

V.W. = very wide; * extrapolation.

Section S12. Selectivity of Compound **1** for *Staphylococci*

Table S6. MICs of **1** against Gram-positive MDR clinical isolates used in this study and its selectivity index values (SIs) in relation to its cytotoxicity against HaCaT and 3T3 cells.

	**1** (433.3) [1]		
**Gram-positive Strains**	**MIC (µg/mL)**	**HaCaT**	**3T3**
S. aureus 18 MRSA	32	**6.77**	**20.53**
S. aureus ATCC 29213	32	**6.77**	**20.53**
S. aureus 189 MRSA	16	**13.54**	**41.06**
S. aureus B MRSA	8	**27.09**	**82.11**
S. epidermidis 22 MRSE	16	**13.54**	**41.06**
S. epidermidis 25 MRSE	64	**3.39**	**10.26**
S. epidermidis 64 MRSE	64	**3.39**	**10.26**
S. epidermidis 147 MRSE	64	**3.39**	**10.26**
E. faecalis 1 VRE *,**	64	**3.39**	**10.26**
E. faecalis 439 VRE *	64	**3.39**	**10.26**
E. faecalis 365 VRE *,**	64	**3.39**	**10.26**
E. faecalis 451VRE *	64	**3.39**	**10.26**
E. faecium 152 VRE *,**	64	**3.39**	**10.26**
E. faecium 183 VRE *,**	64	**3.39**	**10.26**
E. faecium 185 VRE *,**	64	**3.39**	**10.26**
E. faecium 364 VRE *	64	**3.39**	**10.26**

[1] MW of **1**; VRE = vancomycin-resistant enterococci; MRSA = methicillin resistant *S. aureus*; MRSE = methicillin resistant *S. epidermidis*; * denotes resistance also to teicoplanin; ** denotes resistance also to linezolid. In red and bold, numerical values of SI >> 1 indicating substantial selectivity of **1** for bacterial and potential clinical development.

Disclaimer/Publisher's Note: The statements, opinions and data contained in all publications are solely those of the individual author(s) and contributor(s) and not of MDPI and/or the editor(s). MDPI and/or the editor(s) disclaim responsibility for any injury to people or property resulting from any ideas, methods, instructions or products referred to in the contenType of the Paper (Article, Review, Communication, etc.)
